# PPM1D activity promotes cellular transformation by preventing senescence and cell death

**DOI:** 10.1038/s41388-024-03149-3

**Published:** 2024-09-05

**Authors:** Miroslav Stoyanov, Andra S. Martinikova, Katerina Matejkova, Klara Horackova, Petra Zemankova, Kamila Burdova, Zuzana Zemanova, Petra Kleiblova, Zdenek Kleibl, Libor Macurek

**Affiliations:** 1 Cancer Cell Biology, Institute of Molecular Genetics of the Czech Academy of Sciences, Prague, Czech Republic; 2 Department of Cell Biology, Faculty of Science, Charles University, Prague, Czech Republic; 3 Institute of Medical Biochemistry and Laboratory Diagnostics, First Faculty of Medicine, Charles University and General University Hospital in Prague, Prague, Czech Republic; 4 Department of Genetics and Microbiology, Faculty of Science, Charles University, Prague, Czech Republic; 5 Institute of Biology and Medical Genetics, First Faculty of Medicine, Charles University and General University Hospital in Prague, Prague, Czech Republic

**Keywords:** Checkpoint signalling, Oncogenes

## Abstract

Cell cycle checkpoints, oncogene-induced senescence and programmed cell death represent intrinsic barriers to tumorigenesis. Protein phosphatase magnesium-dependent 1 (PPM1D) is a negative regulator of the tumour suppressor p53 and has been implicated in termination of the DNA damage response. Here, we addressed the consequences of increased PPM1D activity resulting from the gain-of-function truncating mutations in exon 6 of the *PPM1D*. We show that while control cells permanently exit the cell cycle and reside in senescence in the presence of DNA damage caused by ionising radiation or replication stress induced by the active RAS oncogene, RPE1-hTERT and BJ-hTERT cells carrying the truncated PPM1D continue proliferation in the presence of DNA damage, form micronuclei and accumulate genomic rearrangements revealed by karyotyping. Further, we show that increased PPM1D activity promotes cell growth in the soft agar and formation of tumours in xenograft models. Finally, expression profiling of the transformed clones revealed dysregulation of several oncogenic and tumour suppressor pathways. Our data support the oncogenic potential of PPM1D in the context of exposure to ionising radiation and oncogene-induced replication stress.

## Introduction

Genome instability is one of the major drivers of tumorigenesis [1]. In the presence of DNA damage, integrity of the genome is protected by arresting the cell cycle progression and by efficient DNA repair [2]. Sustained DNA damage promotes permanent cell cycle exit (called senescence) that acts as an intrinsic barrier against malignant transformation [3–6]. Excessive DNA damage may be caused by environmental factors such as ionising radiation, but commonly occurs also upon activation of oncogenes that trigger replication stress [7, 8]. For instance, overexpression of *CCNE1* oncogene leads to premature G1/S transition, promotes firing of the replication origins, increases the number of conflicts between transcription and replication (TRC), slows-down progression of the replication fork and results in accumulation of the chromosome segregation errors in mitosis [9–11]. Similarly, expression of HRAS^G12V^ oncogene (hereafter referred to as HRASV12) initially leads to accelerated proliferation, increased TRCs, depletion of deoxynucleotide triphosphates, production of reactive oxygen species (ROS) and eventually triggers oncogene-induced senescence (OIS) [12–14]. Tumour suppressor p53 is a downstream effector of DNA damage and oxidative stress response pathways and a master regulator of senescence [15]. Inactivating mutations in *TP53* belong to the most common genetic changes in human solid tumours and also cancers that retain wild-type p53 show defects in regulation of p53 function.

Protein phosphatase magnesium dependent 1 (PPM1D) acts as an efficient negative regulator of p53 pathway by directly dephosphorylating p53-S15, by interfering with p53-p300 interaction leading to decrease of p53-K382 acetylation, by targeting MDM2 and by inactivating its upstream activator ATM kinase [16–20]. By supressing p53 pathway, PPM1D promotes recovery from the cell cycle checkpoint arrest and inversely its inhibition stimulates senescence [21–23]. In addition, PPM1D acts at chromatin flanking the DNA breaks and contributes to control of DNA repair [24–26]. Amplification of *PPM1D* locus is common in breast cancer (over 10% of cases) and is observed mainly in tumours that retain wild-type p53 [27]. In addition, non-sense mutations in exon 6 of the *PPM1D* leading to production of enzymatically active, C-terminally truncated PPM1D protein have been reported in various cancer types including colon cancer and glioma [28–30]. Truncated PPM1D is stabilised at protein level and its activity partially supresses p53 [31]. We and others have previously shown that increased PPM1D activity resulting from the high protein level of the C-terminally truncated PPM1D provides cells with proliferation advantage upon exposure to various forms of genotoxic stress, including ionising radiation, etoposide and cytarabine [29, 31, 32]. Similarly, intestinal and hematopoietic stem cells carrying the truncated *PPM1D* allele showed increased survival after genotoxic stress and promoted APC-driven tumour growth in the intestine and the irradiation-induced acute myeloid leukaemia (AML), respectively [29, 33]. Nevertheless, the precise mechanism of the oncogenic behaviour of PPM1D has yet not been fully addressed.

Here, we used our established diploid cell line models carrying the truncated PPM1D and investigated the long-term consequences on cellular proliferation after exposure to ionising radiation and after induction of *RAS* oncogene. Whereas the control cells arrested in the checkpoint, cells carrying the truncated PPM1D progressed through the cell cycle and accumulated micronuclei. Importantly, these cells formed colonies in the soft agar and tumours in the xenograft models confirming the ability of active PPM1D to transform the cells. Cytogenetic analysis and whole exome sequencing (WES) revealed an accumulation of genetic rearrangements in the surviving cells with truncated PPM1D. Similarly, expression of active RAS induced senescence and cell death in BJ fibroblasts, whereas presence of the truncated PPM1D prevented the cell cycle exit and cell death and promoted cell transformation. In summary, we provide experimental evidence supporting the oncogenic function of PPM1D phosphatase.

## Results

### Cells carrying the truncated PPM1D form micronuclei after ionising radiation

**Fig. 1:**
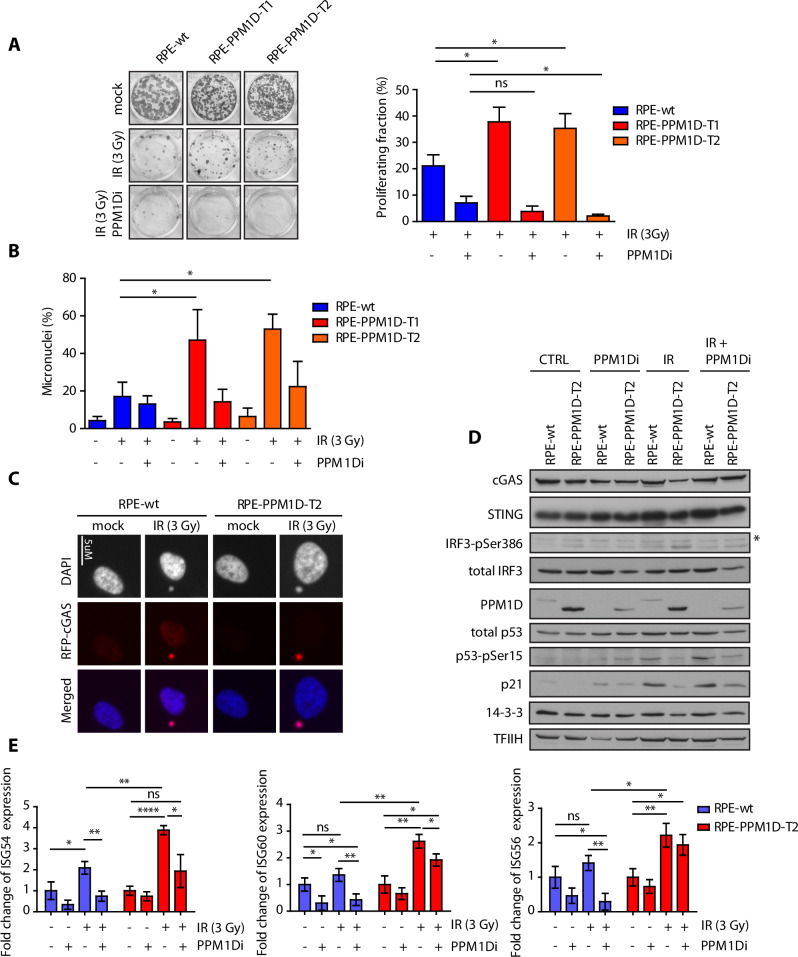
Cells carrying the truncated PPM1D form micronuclei after exposure to ionising radiation. **A** Parental RPE, RPE-PPM1D-T1 and RPE-PPM1D-T2 cells were mock treated or irradiated (3 Gy) in the presence or absence of PPM1Di and further cultured for 10 d. Surviving fraction was calculated by normalising the colony number to the non-treated control for each genotype. Error bars indicate SD. Statistical significance was determined by Student’s *t* test (*p ≤ 0.05, n = 3). **B** Parental RPE, RPE-PPM1D-T1 and RPE-PPM1D-T2 cells were mock treated or irradiated (3 Gy) in presence or absence of PPM1Di and fixed after 48 h. Cells were then stained with DAPI and percentage of cells containing micronuclei was determined microscopically. More than 200 cells per condition were quantified in each experiment (n = 3), error bars indicate SD. Statistical significance was determined by Student’s *t* test (*p ≤ 0.05) **C** Parental RPE and RPE-PPM1D-T2 cells were stably transfected RFP-cGAS and were fixed 48 h after mock treatment or irradiation (3 Gy). Note accumulation of RFP-cGAS in MNs in cells exposed to IR. **D** Parental RPE and RPE-PPM1D-T2 stably transfected with RFP-cGAS were mock treated or irradiated (3 Gy) in presence or absence of PPM1Di. Whole cell lysates were collected after 48 h and analysed by immunoblotting. Asterisk indicates a non-specific reactivity. Signal of IRF3-pSer386 was quantified in ImageJ from 3 independent repeats and was normalised to the loading control (TFIIH) and to the non-treated condition. Statistical significance was determined by Student’s *t* test (*p ≤ 0.05, n = 3, error bars indicate SD). **E** RNA was collected from cells grown as in (**D**) and expression of indicated genes was analysed by qPCR. Statistical significance was calculated by Student’s *t* test (*p ≤ 0.05, **p ≤ 0.01, ****p ≤ 0.0001).

We have previously shown that cells carrying the truncating mutation in exon 6 of the *PPM1D* fail to arrest in the G1 and G2 checkpoints and continue progression through the cell cycle despite the presence of DNA damage [29, 31]. Here, we aimed to investigate the long-term consequences of increased PPM1D activity on proliferation under conditions of genotoxic stress. Using two independent clones of diploid RPE1-hTERT cells carrying the truncated PPM1D, we observed that they formed significantly more colonies 10 days after exposure to ionising radiation (Fig. 1A). This proliferation advantage of RPE1-PPM1D-T1 and RPE1-PPM1D-T2 cells was lost upon inhibition of PPM1D activity (Fig. 1A). In addition, we noticed that about a half of RPE1-PPM1D-T1 and RPE1-PPM1D-T2 cells contained micronuclei (MN) 48 h after exposure to IR and that inhibition of PPM1D decreased the fraction of cells with micronuclei (Fig. 1B). This observation is consistent with the mitotic defects occurring in the cells that proliferate in the presence of unrepaired DNA lesions. It is well established that the permeable nuclear envelope of MNs allows contact of the DNA with the cytosol leading to activation of cyclic GMP-AMP synthase (cGAS) and triggering the cGAS/STING signalling pathway [34–36]. Interferon regulatory factor 3 (IRF3) is a downstream effector of cGAS/STING pathway that promotes expression of type I interferons and inflammatory cytokines [36, 37]. In RPE1 cells, we could not detect endogenous cGAS but expression of cGAS-RFP allowed visualisation of the MNs in RPE1-PPM1D-T1 cells (Fig. 1C). In contrast to the parental RPE cells, RPE1-PPM1D-T2 cells showed increased level of IRF3 phosphorylated at pSer386, which indicates activation of the cGAS pathway after exposure to ionising radiation (Fig. 1D, Supplementary Figs. S1, S2A, B). Similarly, compared to the parental cells, RPE1-PPM1D-T2 cells showed increased expression of three Interferon-stimulated genes ISG54, ISG56 and ISG60 that are downstream targets of the cGAS pathway (Fig. 1E). Modification of IRF3 as well as the expression of ISGs was reduced upon inhibition of PPM1D indicating that PPM1D activity promotes formation of MNs and activation of the cGAS pathway (Fig. 1D, E). The observed suppression of cGAS pathway by PPM1D inhibition likely reflects the checkpoint arrest that prevents chromosomal missegregation in mitosis [38].

### PPM1D activity promotes cell transformation after exposure to ionising radiation

**Fig. 2:**
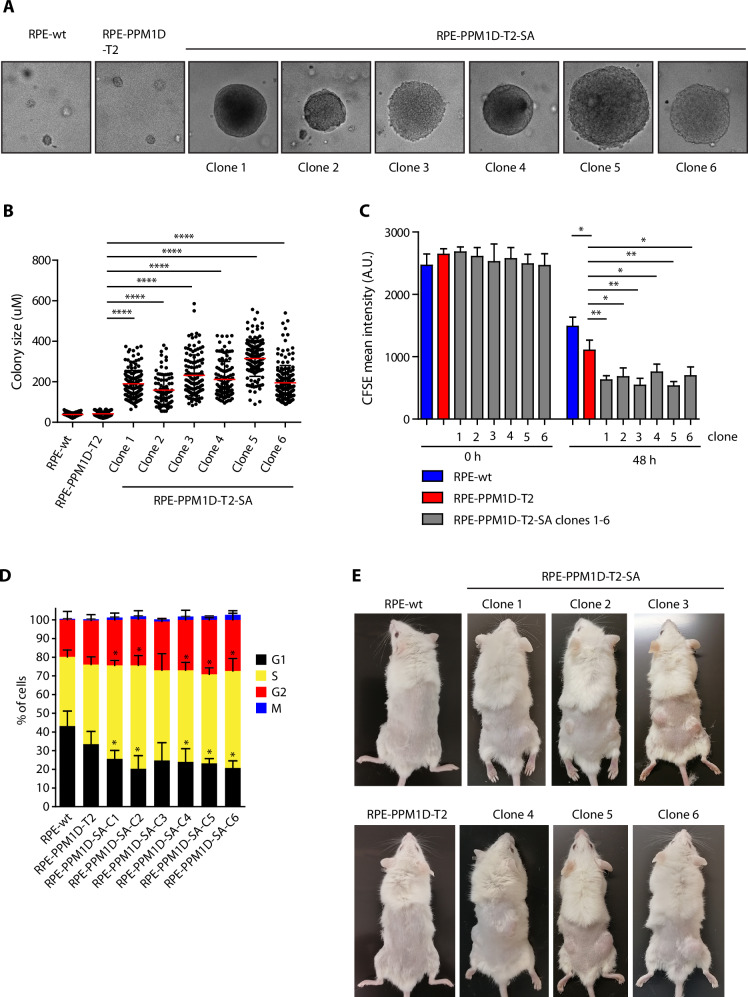
PPM1D activity promotes cell transformation after exposure to ionising radiation. **A** Parental RPE and RPE-PPM1D-T2 cells were mock treated or irradiated (3 Gy) and were grown in semi-solid media for 12 weeks. Six independent clones of RPE-PPM1D-T2-SA cells were collected. **B** Colony size of parental RPE cells, parental RPE-PPM1D-T2 cells and transformed RPE-PPM1D-T2 clones was acquired after propagating in the semisolid media for 2 weeks. Each dot represents a single colony, red line indicates mean colony size, bars show SD, n = 2. **C** Cell division was monitored by labelling of cells with CFSE. A zero time point was collected to determine the initial labelling and the rest of the samples were collected after 48 h. Cell were then collected and fixed and analysed by FACS. Plotted is the mean intensity of CFSE signal, error bars indicate SD, n = 3. Statistical significance was calculated using Student’s *t* test (*p ≤ 0.05, **p ≤ 0.01). **D** Cell cycle distribution was determined in parental RPE, parental RPE-PPM1D-T2 cells and transformed RPE-PPM1D-T2 clones using flow cytometry. Plotted are fractions of cells in G1, S, G2 and M phases of the cell cycle. Error bars indicate SDs, n = 3. **E** Nude mice were subcutaneously injected with parental RPE cells, parental RPE-PPM1D-T2 cells and transformed RPE-PPM1D-T2 clones and tumour growth was evaluated after 3 weeks.

Next, we aimed to address the long-term effect of ionising radiation in cells with partially inactivated cell cycle checkpoints due to the truncated PPM1D. To this end, we exposed parental RPE1-hTERT and RPE-PPM1D-T2 cells to 3 Gy of ionising radiation, seeded them in semisolid medium and monitored the clonal growth. Whereas all parental RPE1 cells died, several RPE-PPM1D-T2 clones occurred after one month of culture indicating that PPM1D promotes anchorage-independent cell growth (Fig. 2A). Altogether, we picked six RPE-PPM1D-T2 spheroid clones (hereafter referred to as RPE-PPM1D-T2-SA clones 1–6) from the soft agar cultures and confirmed their ability to grow in semisolid media in subsequent cultures (Fig. 2B). Interestingly, we noted that all transformed RPE-PPM1D-T2-SA clones showed faster population doubling compared to the nontransformed RPE-PPM1D-T2 cells and parental RPE cells, confirming that these cells gathered proliferation advantage during the culture in the soft agar (Fig. 2C). In addition, flow cytometry analysis revealed that a significantly lower fraction of the transformed RPE-PPM1D-T2-SA cells resided in G1 phase of the cell cycle compared to the parental RPE cells, while more S phase cells were observed (Fig. 2D). The cell cycle distribution of the nontransformed RPE-PPM1D-T2 cells was not statistically different from that of the parental RPE cells and the transformed cells (Fig. 2D). These data are consistent with shortening the G1 while extending duration of DNA replication in the transformed RPE-PPM1D-T2-SA cells and suggest that the trend to accelerate progression through the G1 due to the truncation of PPM1D is further enhanced by additional changes during cellular transformation. Finally, we found that all six RPE-PPM1D-T2-SA clones recovered from the soft agar (but not the RPE-PPM1D-T2 cells) formed tumours in xenograft models confirming a completion of the cell transformation (Fig. 2E). We conclude that PPM1D activity promoted cell transformation after exposure to ionising radiation.

### PPM1D activity promotes genomic rearrangements after exposure to ionising radiation

**Fig. 3:**
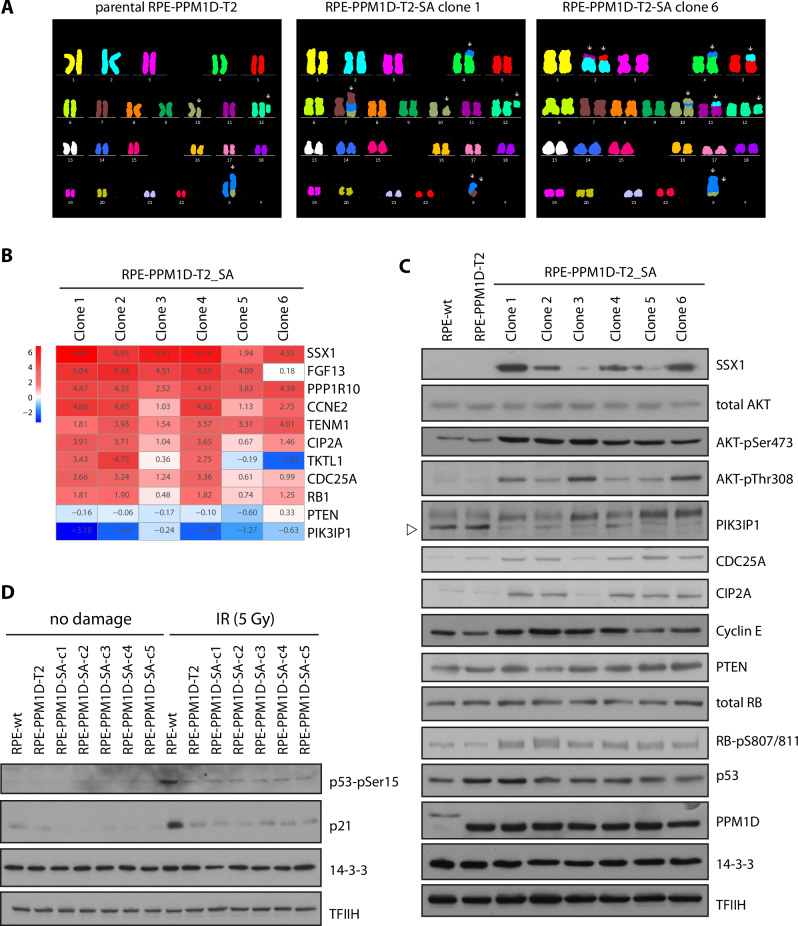
Genomic rearrangements and differential expression in cells with high PPM1D activity. **A** Parental RPE-PPM1D-T2 cells and transformed RPE-PPM1D-T2-SA (clones 1 and 6) were arrested in mitosis, fixed and probed by M-FISH. Karyotyping of the remaining clones is shown in Suppl. Figure 2A. **B** Selected genes differentially expressed in parental RPE-PPM1D-T2 and transformed RPE-PPM1D-T2-SA cells. The heat map shows the differential expression normalised to nontransformed parental RPE-PPM1D-T2 cells. **C** Asynchronously growing parental RPE, RPE-PPM1D-T2 and transformed RPE-PPM1D-T2-SA clones 1–6 were lysed and expression of selected proteins was determined by immunoblotting. TFIIH and 14-3-3 were used as loading controls. Arrowhead indicates the position of PIK3IP protein. **D** Parental RPE, RPE-PPM1D-T2 and transformed RPE-PPM1D-T2-SA clones 1–6 were exposed or not to a high dose of IR (5 Gy), collected after 6 h and whole cell lysates were analysed by immunoblotting.

Random integration of the DNA content of the MNs into the genomic DNA can cause massive genomic rearrangements called chromothripsis that has previously been implicated in tumourigenesis [39–43]. To analyse the genomic changes, we arrested the parental and transformed RPE-PPM1D-T2-SA clones in mitosis by colcemid and performed the cytogenetic analysis using multiplex fluorescence in situ hybridisation [44]. Parental RPE1 and non-transformed RPE-PPM1D-T2 cells are nearly diploid and exhibit a translocation of a duplicated chromosome 10 (10q21.2 to 10qter) onto the microdeleted telomere region of chromosome X [45]. In contrast, the transformed RPE-PPM1D-T2-SA clones showed aberrant karyotypes with several translocations and deletions. Most striking rearrangements occurred on the chromosome X that was translocated to various genomic loci in individual transformed RPE-PPM1D-T2-SA clones (Fig. 3A, Supplementary Fig. S3). Interestingly, the distinct spectrum of genomic rearrangements present in individual clones suggests that these evolved independently (Fig. 3A, Supplementary Fig. S3).

Next, we performed transcriptomic analysis of the parental and transformed RPE-PPM1D-T2 cells. RNA-seq analysis identified 8871, 8597, 402, 8659, 1253 and 6560 differentially expressed transcripts (with fold change FC > 3 and FC < 0.3, p_adj_ < 0.05) in RPE-PPM1D-T2-SA clones 1–6 compared to the non-transformed RPE-PPM1D-T2, respectively (Suppl. Fig. S4, Suppl. Table 1). Unsupervised clustering analysis revealed that clones 1, 2 and 4 showed similar expression pattern whereas clones 5, 6 and 3 showed more distinct expression profiles (Supplementary Fig. S5). When we compared the top scoring upregulated genes, we found that several X chromosomal genes (including SSX1, FGF13, TKTL1 and TENM1) were highly expressed in most of the RPE-PPM1D-T2-SA clones (Fig. 3B). Translocation t(X;18)(p11.2;q11.2) leading to the SSX1-SYT fusion has been implicated in development and invasiveness of synovial sarcoma [46, 47]. We did not detect the SSX1-SYT fusion protein in the transformed RPE-PPM1D-T2-SA clones (data not shown). Nevertheless, we confirmed high level of SSX1 protein in four of the transformed RPE-PPM1D-T2-SA clones (Fig. 3C). We hypothesise that increased expression of the X chromosomal genes is likely caused by translocation to the genomic loci with active chromatin. In addition, we noted increased expression of CCNE2 (cyclin E2) and CDC25A oncogenes in most of the transformed RPE-PPM1D-T2-SA clones (Fig. 3B, C). We have recently demonstrated that PPM1D activity further accelerates G1/S transition by supressing p53 pathway in cells overexpressing cyclin E and thus increases the level of replication stress suggesting that the increased levels of cyclin E in RPE-PPM1D-T2-SA clones likely explain the reduced fraction of the G1 cells observed in Fig. 2D [48]. Similarly, CDC25A phosphatase has been implicated in replication stress and breast cancer [49, 50]. Finally, we found that five of the transformed RPE-PPM1D-T2-SA clones expressed high level of CIP2A oncogene that was previously shown to promote anchorage-independent cell growth [50] (Fig. 3B, C).

Next, we searched for possible loss of the tumour suppressors in the transformed RPE-PPM1D-T2 cells. In particular, we focused on tumour suppressor protein p53, which is commonly mutated in human cancers and its loss promotes cell transformation upon genotoxic stress [51, 52]. Interestingly, we found that all six RPE-PPM1D-T2-SA clones expressed comparable levels of *CDKN1A* transcript and contained comparable levels of p21 protein after exposure to ionising radiation as the parental RPE-PPM1D-T2 cells indicating that they retained the ability to activate p53 pathway (Fig. 3D, Supplementary Fig. S6). The presence of the wild type p53 in the RPE-PPM1D-T2-SA clones was confirmed by sequencing of *TP53* from the genomic DNA (Supplementary Table 2). As p53 is a direct target of PPM1D, its function is partially supressed in the presence of truncated PPM1D and therefore there is likely no selection pressure for p53 mutation during cell transformation [19, 20, 31, 53]. These observations are consistent with the normal p53 status frequently observed in cancers with pathologically increased PPM1D levels [27, 54]. In addition, we found that *RB1* and *PTEN* tumour suppressors remained intact in all clones (Supplementary Table 2). Nevertheless, we observed decreased expression of phosphoinositide-3-kinase-interacting protein 1 (PIK3IP1) in most of the transformed RPE-PPM1D-T2-SA clones compared to the parental non-transformed cells. Suppression of PIK3IP1 expression has previously been implicated in activation of PI3K/AKT/mTOR pathway [55]. Indeed, increased AKT phosphorylation revealed activation of PI3K/AKT/mTOR pathway in all the transformed RPE-PPM1D-T2-SA cells (Fig. 3C). Altogether, several pathways previously implicated in anchorage independent cell growth and oncogenesis are upregulated in individual transformed RPE-PPM1D-T2-SA clones.

Finally, to search for other deregulated pathways in the transformed RPE-PPM1D-T2-SA cells, we performed the GSEA analysis and compared the individual clones with the parental RPE-PPM1D-T2 cells. This analysis revealed 1314, 1217, 115, 1488, 76, and 18 significantly different datasets in RPE-PPM1D-T2-SA clones 1–6, respectively (Supplementary Table 3). To reduce complexity of the redundant GO annotations, we used rrvgo tool, allowing to identify numerous similar significantly-enriched GO clusters (Supplementary Figs. S6–S8) in clones 1, 2, and 4 (including pathways related to DNA repair/replication processes and immune responses), contrasting with only few different GO clusters in the clones 3 and 5 and no significant cluster in the clone 6 (Supplementary Fig. S8). These results suggest that divergent malignant transformation trajectories occurred in at least two groups of clones, first including clones 1, 2, and 4 and the second consisting of clones 3, 5, and 6.

### PPM1D activity allows accumulation of genomic changes upon induction of replication stress

**Fig. 4:**
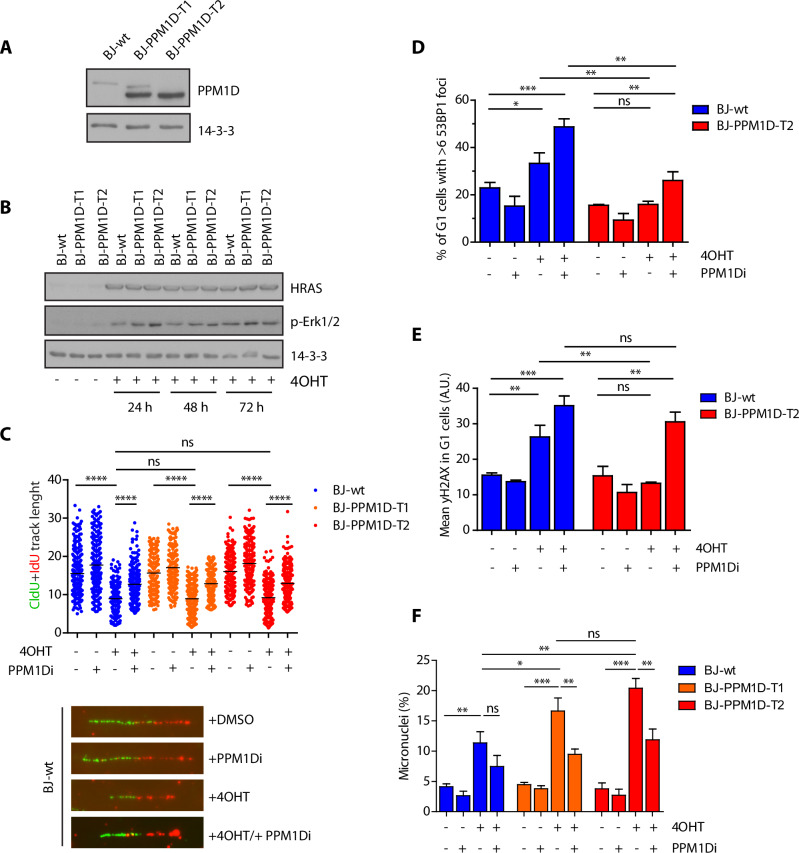
Impact of PPM1D activity on RAS-induced replication stress. **A** BJ-hTert-HRASV12^ER-TAM^ and two clones expanded after targeting the exon 6 of *PPM1D* by CRISPR/Cas9 were analysed by immunoblotting. Staining for actin was used as loading control. **B** BJ-hTert-HRASV12^ER-TAM^, BJ-hTert-HRASV12^ER-TAM^-PPM1D-T1 and BJ-hTert-HRASV12^ER-TAM^-PPM1D-T2 cells were induced with 4OHT for indicated times and whole cell lysates were analysed by immunoblotting. Staining for 14-3-3 protein was used as loading control. **C** BJ-hTert-HRASV12^ER-TAM^, BJ-hTert-HRASV12^ER-TAM^-PPM1D-T1 and BJ-hTert-HRASV12^ER-TAM^-PPM1D-T2 were induced with 4OHT for 2 d and were labelled with CldU and IdU 20 min. Plotted is the sum of the length (μm) of CldU and IdU labelled tracks, each dot represents one replication fork, n = 3. Black line indicates mean. Statistical significance was calculated by Student’s *t* test (****p ≤ 0.0001). **D** BJ-hTert-HRASV12^ER-TAM^ and BJ-hTert-HRASV12^ER-TAM^-PPM1D-T2 cells were induced with 4OHT for 5 d and formation of 53BP1 nuclear foci was evaluated by ScanR microscopy. Plotted is a fraction of cells with ≥6 53BP1 nuclear foci. More than 300 cells were quantified per experiment, bars indicate SD, n = 3. Statistical significance was calculated by Student’s *t* test (*p ≤ 0.05, **p ≤ 0.01, ***p ≤ 0.001). **E** Cells from (**D**) were probed for γH2AX and analyzed by ScanR microscopy. Plotted is mean nuclear signal of γH2AX in G1 cells. Statistical significance was calculated by Student’s *t* test (**p ≤ 0.01, ***p ≤ 0.001). **F** BJ-hTert-HRASV12^ER-TAM^, BJ-hTert-HRASV12^ER-TAM^-PPM1D-T1 and BJ-hTert-HRASV12^ER-TAM^-PPM1D-T2 induced with 4OHT for 5 d. Fraction of cells containing MN was determined microscopically. Bars indicate SD, more than 150 cells per condition were quantified in each experiment, n = 3. Statistical significance was calculated by Student’s *t* test (ns p > 0.05; *p ≤ 0.05, **p ≤ 0.01, ***p ≤ 0.001).

Data described above support the oncogenic role of PPM1D in context of genotoxic stress and are in agreement with our recent observation of IR-induced leukaemia in mice carrying the truncated *PPM1D* allele and with the observed high frequency of *PPM1D* mutations in patients suffering from therapy-induced haematological malignancies [32, 33, 56]. Next, we aimed to broaden the analysis of the truncating PPM1D mutations towards oncogene-induced replication stress, which is another physiologically relevant context for cancer development. To this end, we used BJ-hTert-HRASV12^ER-TAM^ cells with inducible expression of an active form of HRAS oncogene upon treatment with 4-hydroxy tamoxifen (4OHT) [12, 57]. Using CRISPR/Cas9 technology, we introduced a frameshift mutation in exon 6 of *PPM1D* and confirmed the expected protein stabilisation of the C-terminally truncated PPM1D in two independent clones (Fig. 4A). Upon induction with 4OHT, cells started expressing HRASV12 and reached a plateau after about two days (Fig. 4B). Induction of HRASV12 is known to induce replication stress caused by conflicts between replication and transcription [12]. In agreement with this, we found that induction of HRASV12 slowed down progression of the replication forks measured by a DNA fibre assay (Fig. 4C). Importantly, we did not observe any significant differences between the parental BJ-hTert-HRASV12^ER-TAM^ and BJ-hTert-HRASV12^ER-TAM^-PPM1D-T1 and -T2 cells suggesting that both cell types experienced a comparable level of replication stress (Fig. 4C). During mitosis, under-replicated regions are converted into DNA lesions that are protected by 53BP1 protein throughout the subsequent G1 phase of the cell cycle until they are eventually repaired in the S phase [58, 59]. Formation of 53BP1 nuclear foci in G1 cells therefore reflects the replication problems in the previous cell cycle. Interestingly, we observed that BJ-hTert-HRASV12^ER-TAM^-PPM1D-T2 cells treated with 4OHT showed lower fraction of cells with nuclear 53BP1 foci compared to the parental BJ-hTert-HRASV12^ER-TAM^ cells (Fig. 4D). Decreased formation of 53BP1 foci in BJ-hTert-HRASV12^ER-TAM^-PPM1D-T2 cells likely reflects PPM1D-mediated dephosphorylation of ATM and γH2AX that are both needed for recruitment of 53BP1 to the proximity of DNA lesions (Fig. 4E) [25, 60]. We hypothesised, that cells containing the high level of PPM1D activity may accumulate an increased amount of genomic changes upon replication stress due to decreased ability to recognise DNA lesions and to continued cell proliferation. Indeed, we observed that both BJ-hTert-HRASV12^ER-TAM^-PPM1D-T1 and -T2 clones accumulated more MNs compared to the parental BJ-hTert-HRASV12^ER-TAM^ cells and importantly, formation of the MNs was reduced by inhibition of PPM1D (Fig. 4F). We conclude that PPM1D activity promotes accumulation of genomic changes not only after ionising radiation but also under conditions of replication stress.

### PPM1D activity impairs oncogene induced senescence and promotes cell transformation

**Fig. 5:**
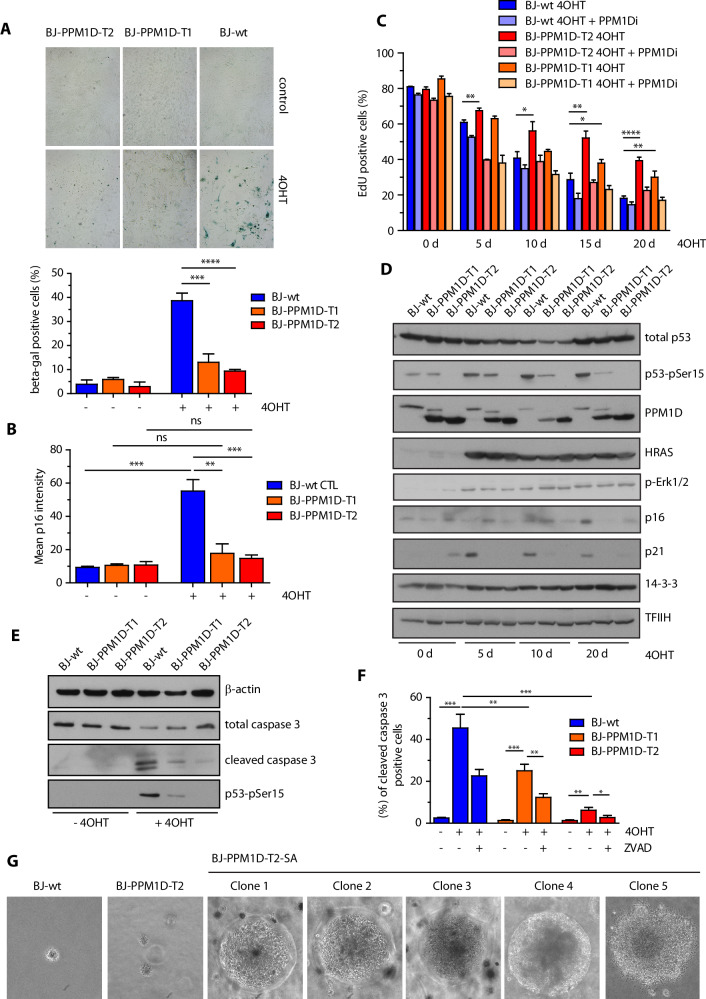
PPM1D activity impairs OIS and promotes cell transformation. **A** BJ-hTert-HRASV12^ER-TAM^, BJ-hTert-HRASV12^ER-TAM^-PPM1D-T1 and BJ-hTert-HRASV12^ER-TAM^-PPM1D-T2 cells were induced or not with 4OHT for 20 d and stained for β-galactosidase activity. Plotted is the fraction of β-gal positive cells, bars indicate SD, n = 3. Statistical significance was calculated by Student’s *t* test (***p ≤ 0.001, ****p ≤ 0.0001). **B** Cells grown as in A were fixed and stained for p16. Plotted is the mean nuclear p16 intensity, bars indicate SD. More than 300 cells of each condition were quantified per experiment, n = 3. Statistical significance was calculated by Student’s *t* test (**p ≤ 0.01, ***p ≤ 0.001). **C** Cells as in A were induced with 4OHT for 5 to 20 days were incubated with EdU 24 h prior fixation followed by CLICK-IT reaction. Fraction of EdU positive cells was analysed using FACS. Bars indicate SD, n = 3. Statistical significance was calculated by Student’s *t* test (*p ≤ 0.05, **p ≤ 0.01, ****p ≤ 0.0001). **D** Whole cell lysates from cells from (**C**) were analyzed by immunoblotting using indicated antibodies. **E** BJ-hTert-HRASV12^ER-TAM^, BJ-hTert-HRASV12^ER-TAM^-PPM1D-T1 and -T2 cells were treated or not with 4OHT for 5 days. Whole cell lysates were probed with indicated antibodies by immunoblotting. **F** Cells treated as in (**E**) were analysed by flow cytometry. Where indicated, cells were incubated in the presence of Z-VAD-FMK. Plotted is the fraction of cleaved caspase 3 positive cells. Bars indicate SD, n = 3. Statistical significance was calculated by Student’s *t* test. **G** Parental BJ-hTert-HRASV12^ER-TAM^, parental BJ-hTert-HRASV12^ER-TAM^-PPM1D-T2, and BJ-hTert-HRASV12^ER-TAM^-PPM1D-T2-60 cells that survived 2-month continuous induction with 4OHT were cultured in semisolid media for 10 weeks.

A long-term outcome of expression of the active RAS oncogene is senescence caused by accumulation of DNA damage during replication [8]. OIS depends on activation of p53 pathway and p16^INKa^/p16 (CDKN2A) inhibitor of the cyclin dependent kinases [15]. As expected, treatment of BJ-hTert-HRASV12^ER-TAM^ cells with tamoxifen for 20 days massively increased the fraction of β-galactosidase positive cells and induced the expression of p16, which is consistent with induction of senescence (Fig. 5A, B). In contrast, induction of β-galactosidase and p16 was strongly reduced in BJ-hTert-HRASV12^ER-TAM^-PPM1D-T1 and -T2 cells indicating that they are more resistant to senescence (Fig. 5A, B). Similarly, we observed that parental BJ-hTert-HRASV12^ER-TAM^ cells contained higher levels of histone H3K9me3 and HP1, and contained enlarged nuclei confirming that they are more prone to senescence compared to BJ-hTert-HRASV12^ER-TAM^-PPM1D-T2 cells (Supplementary Fig. S10A–D) [61]. In addition, we observed a gradual decrease of EdU incorporation in BJ-hTert-HRASV12^ER-TAM^ cells after treatment for 5–20 days indicating that they eventually exited the cell cycle (Fig. 5C). On the other hand, significantly more BJ-hTert-HRASV12^ER-TAM^-PPM1D-T1 and -T2 cells incorporated EdU after 20 days of 4OHT induction, suggesting that they continued cell proliferation (Fig. 5C). Inhibition of PPM1D prevented EdU incorporation in BJ-hTert-HRASV12^ER-TAM^-PPM1D-T1 and -T2 cells indicating that PPM1D was needed for overriding the OIS (Fig. 5C). Whereas expression of RAS induced the levels of p21 after 5 days and of p16 after 20 days in control cells, we noted lower levels of p21 and p16 in BJ-hTert-HRASV12^ER-TAM^-PPM1D-T1 and -T2 cells, which is consistent with impaired induction of the checkpoint and senescence (Fig. 5D). Interestingly, when we knocked out p53 in BJ-hTert-HRASV12^ER-TAM^ cells or in BJ-hTert-HRASV12^ER-TAM^-PPM1D-T cells, they incorporated EdU even after extended treatment with 4OHT and they also became resistant to PPM1D inhibition (Supplementary Fig. S11A). We conclude that PPM1D promotes override of OIS by supressing p53 pathway. Of note, increased PPM1D activity in BJ-hTert-HRASV12^ER-TAM^-PPM1D-T1 and -T2 cells is not as efficient in promoting cell proliferation as complete loss of p53 suggesting that PPM1D can only partially supress p53 function. In addition to OIS, we observed that substantial proportion of control BJ-hTert-HRASV12^ER-TAM^ cells died upon induction of RAS expression (Supplementary Fig. S11B). These findings are in line with a variable level of cell death reported after expression of RAS oncogene in various cellular systems [62–64]. Importantly, we observed that the truncated PPM1D protected BJ-hTert-HRASV12^ER-TAM^-PPM1D-T1 and -T2 cells from the RAS-induced cell death (Supplementary Fig. S11B). Treatment of BJ-hTert-HRASV12^ER-TAM^ cells with 4OHT, induced the cleavage of caspase 3 and this effect was blocked by a caspase inhibitor Z-VAD-FMK confirming that induction of active RAS induced apoptosis (Fig. 5E, F). In contrast, the levels of cleaved caspase 3 were signifinactly reduced in BJ-hTert-HRASV12^ER-TAM^-PPM1D-T1 and -T2 cells suggesting that PPM1D activity protect cells from apoptosis (Fig. 5E, F). Whereas expression of two pro-apoptotic p53 targets *NOXA* and *BAX* was strongly induced in 4OHT- treated BJ-hTert-HRASV12^ER-TAM^ cells, expression was significantly lower in BJ-hTert-HRASV12^ER-TAM^-PPM1D-T2 cells supporting the model in which PPM1D activity protects form cell death by inhibiting p53 pathway (Supplementary Fig. S11C).

Interestingly, BJ-hTert-HRASV12^ER-TAM^-PPM1D-T1 and -T2 cells survived induction with 4OHT and after initial slowdown in proliferation, they grow rapidly after 60 days (Supplementary Fig. S11D). These cells showed low levels of senescence markers, including p16, histone H3K9me, HP1 and had normal nuclear size (Supplementary Fig. S10A–D). In addition, these cells retained the ability to activate p53 pathway, judged from p21 induction upon exposure to ionising radiation (Supplementary Fig. S11E). Finally, we noted that BJ-hTert-HRASV12^ER-TAM^-PPM1D-T2 cells treated for more than 2 months with 4OHT managed to grow in semisolid media, while the non-induced hTert-HRASV12^ER-TAM^-PPM1D-T2 died (Fig. 5G). In the summary, we conclude that increased PPM1D activity promotes accumulation of genomic changes during replication stress by overriding the OIS and eventually leading to cell transformation.

## Discussion

Based on the commonly observed amplification of the *PPM1D* locus in human cancers as well as the phenotypes of *PPM1D*^*-/-*^ mice that are resistant to tumour development, PPM1D has been proposed to act as an oncogene. On the other hand, mice that overexpress PPM1D or contain gain-of-function truncating mutations in exon 6 of *PPM1D* show only mild reduction of overall survival, suggesting that increased PPM1D activity may not be sufficient to transform cells efficiently [29, 33, 65]. It is thus possible that the oncogenic potential of the truncating *PPM1D* mutations becomes physiologically relevant upon genotoxic stress. Patients suffering from therapy-induced malignancies (mainly t-AML and myelodysplastic syndrome) show increased frequency of truncating *PPM1D* mutations and we have recently reported similar phenotype in transgenic mice [32, 33, 66]. Nevertheless, the driving force underlying the transformation of the PPM1D expressing cells has remained unclear. In this study, we show that cells carrying the truncated PPM1D proliferate in the presence of low dose of DNA damage and, as result, they accumulate genomic rearrangements. As we observed frequent chromosome bridges in cells carrying the truncated PPM1D after exposure to ionising radiation, we favour the possibility that genome rearrangements occurred by repeated breakage-fusion-bridge cycles (BFB) originally described by McClintock [67]. Surprisingly, the RPE-PPM1D-T2-SA clones transformed without reaching the extensive level of genome rearrangements characteristic for chromothripsis [68]. Sequencing of the transformed clones revealed increased expression of several oncogenes (including *CCNE2*, *CDC25A* and *CIP2A*) and decreased expression of tumour suppressors providing a rationale for the observed cellular transformation. Interestingly, all six transformed clones retained the wild type p53 confirming previous observations that PPM1D activation and loss of p53 in tumours tend to be mutually exclusive [69].

Early after induction of DNA damage, we observed increased formation of the micronuclei in RPE-PPM1D-T cells. In our hands, the expression level of cGAS was below detection and the IR-induced transformation of RPE-PPM1D-T cells happened in the absence of detectable cGAS/STING pathway activation. On the other hand, we clearly observed induction of cGAS/STING target genes when we reintroduced cGAS into RPE-PPM1D-T2 cells indicating that the micronuclei formed in these cells have the potential to activate the pathway. We conclude that cGAS/STING is not required for transformation of cells in vitro. However, activation of cGAS/STING pathway may become relevant in context of the tissue microenvironment where inflammation may positively or negatively regulate the tumorigenesis. This possibility remains to be addressed by future research.

Although genotoxic stress is relevant for some specific cancer types, most tumours develop without any apparent external source of DNA damage. In that regard, cellular responses to oncogenes are likely physiologically more relevant for tumorigenesis. Interestingly, we observed dramatic differences in the cell fate of control BJ-hTert-HRASV12^ER-TAM^ and BJ-hTert-HRASV12^ER-TAM^-PPM1D-T cells after induction of active RAS oncogene. Whereas control cells remain permanently arrested in oncogene-induced senescence, cells carrying truncated PPM1D override this barrier and continue to proliferate. Similarly to exposure to ionising irradiation, induction of RAS was associated with formation of the micronuclei suggesting that continuous proliferation of BJ-hTert-HRASV12^ER-TAM^-PPM1D-T cells promotes genome instability. Finally, we find that BJ-hTert-HRASV12^ER-TAM^-PPM1D-T2 cells expressing RAS were able to grow in soft agar confirming the transforming capacity of PPM1D. Although, BJ-hTert-HRASV12^ER-TAM^-PPM1D-T2 homozygotes showed stronger inactivation of p53 function, also BJ-hTert-HRASV12^ER-TAM^-PPM1D-T1 heterozygotes escaped the proliferation arrest. Overall, our results support the oncogenic role of PPM1D not only after exposure to genotoxic stress but also in context of replication stress caused by active RAS oncogene.

## Materials and methods

### Antibodies and reagents

The following antibodies were used: 53BP1 (sc-22760), HP1 (sc-515341), p21 (sc-6246), p53 (sc-126), PPM1D (sc-376257), 14-3-3 (sc-133233), TFIIH (sc-293), Cyclin E (sc-247), RB (sc-102), caspase 3 (sc-7272) from Santa Cruz Biotechnology (Dallas, TX); p16 (#18769S), cGAS (#15102), STING (#13647), IRF3 (#11904), IRF3-pSer386 (#37829), p53-pSer15 (#82530), RB-pSer807/811 (#8516), AKT (#9272), AKT-pThr308 (#4056S), AKT-pSer473 (#4058), CIP2A (#14805), PTEN (#9188), SSX1 (#23855), cleaved caspase 3 (Asp175, #9664S) from Cell Signalling Technology (Danvers, MA); HRAS (GTX116041) and CDC25A (#GTX102308) from GeneTex (Irvine, CA); BrdU/Idu (#347580) from BD Biosciences (Franklin Lakes, NJ); BrdU/CldU (ab6326) from Abcam (Cambridge, UK); Histone H3-trimethyl(Lys9) (#07-442) from Millipore; PIK3IP1 (#16826-1-AP) from Proteintech (Planegg-Martinsried, Germany); secondary antibodies conjugated with Alexa were from Thermo Fisher Scientific (Waltham, MA), Anti-rat Cy3 (712-166-1530) from Immuno Research (Mendota Heights, MN). Custom-made mouse monoclonal antibody to PPM1D was generated by immunising Balb/c mice with purified full-length human His-PPM1D followed by fusion of splenocytes with Sp2/0 myeloma cells following standard procedures. Tissue culture supernatants from hybridomas that showed reactivity in ELISA assay were subsequently tested for the ability to recognise human and mouse PPM1D by flow cytometry and immunoblotting. Epitope of the PPM1D antibody (clone 5) was mapped to a region between amino acids 385–399 of human PPM1D using synthetic Pepspots peptides immobilised on nitrocellulose membrane (JPT Peptide Technologies, Berlin, Germany). GSK2830371 (referred to as PPM1D inhibitor; final concentration 2 μM, [70, 71]) and MDM2 antagonist nutlin-3 (final concentration 9 μM, [72]) and caspase inhibitor Z-VAD-FMK (final concentration 10 μM, [73]) were from MedchemExpress (Monmouth Junction, NJ) and were dissolved in DMSO.

### Cells

Human immortalised retinal pigment epithelia cells hTERT RPE-1 (CRL-4000, hereafter referred to as RPE) were from ATCC and their derivatives RPE-PPM1D-T1 and RPE-PPM1D-T2 each carrying a truncating mutation in exon 6 of *PPM1D* were described previously and were formerly referred to as RPE-PPM1D cr1.1 and cr2.3, respectively [29]. Cells were grown in high glucose DMEM supplemented with 10% FBS, Penicillin (100 U/ml) and Streptomycin (0.1 mg/ml). All cell lines were regularly checked for mycoplasma contamination (Lonza) and were confirmed as negative. BJ-hTert HRASV12^ER-TAM^ cells were described previously and expression of HRAS was induced for indicated times by 4-OH tamoxifen final concentration of 350 nM (4OHT, Sigma Aldrich, St. Louis, MO) [12, 57]. Truncating mutations in exon 6 of PPM1D were introduced in BJ-hTert HRASV12^ER-TAM^ cells by transfecting them with pSpCas9(BB)-2A-Puro (PX459) plasmid carrying sgRNA sequence ATAGCTCGAGAGAATGTCCA followed by selection with puromycin and clonal expansion. Sequencing of the genomic DNA confirmed presence of a frameshifting mutation in exon 6 of the *PPM1D* in clone T1 (heterozygote) and clone T2 (homozygote). Alternatively, knock-out of p53 in BJ-hTert HRASV12^ER-TAM^ cells was generated by co-transfection of CAUUGCUUGGGACGGCAAGG sgRNA (70 nM, Sigma) targeting the exon 4 of *TP53* with purified TrueCut protein Cas9 v2 using CRISPRMAX (both Thermo Fisher Scientific) and cultivating cells in the presence of nutlin-3 (9 μM) for 30 days. Cells surviving in the presence of nutlin-3 were clonally expanded and clones staining negative for p53 were selected.

### Cell division assay

Cells grown in 6-well plates were washed 2 times with PBS and labelled with 5-(6)-Carboxyfluorescein Diacetate Succinimidyl Ester (CFSE, 2 μM, Thermo Scientific) in 0.1% FBS in PBS for 8 min at 37 °C. Labelling was then stopped by addition of FBS directly in each well, after which the plates were further incubated at 37 °C for 5 min for efflux. Finally, the solution was removed and cells were washed 2 times with 2% FBS in PBS. A zero time-point cells were collected for analysing the initial labelling and the rest of the cells were further incubated for 48 h. Then, they were collected and fixed in 4% PFA for 15 min, washed 2 times with PBS and stained with DAPI for 10 min. Finally, the cells were analysed by flow cytometry using excitation/emission filters for Alexa Fluor 488.

### Colony formation assay

Cells (1000/well) were seeded in triplicates on 6-well plates in presence or absence of GSK2830371. On the following day, the plates were irradiated or not with 3 Gy IR. After 10 days, cells were fixed with crystal violet solution for 10 min at room temperature and washed with distilled water until clear colonies were visible. Colonies were counted semi-automatically using the Multi-point tool in ImageJ [74]. Plating efficiency (PE) was determined as a number of colonies formed divided by the number of cells seeded for each well. The proliferating fraction (PF) was calculated as the ratio between the PE of the irradiated cells and PE of the non-treated controls of the same genotype and was multiplied by 100 to obtain percentage. Statistical significance was determined from three biological replicates using Student’s t-test.

### Soft agar assay

Cell culture in semisolid media was performed as previously described [75]. Parental RPE and RPE-PPM1D-T2 cells were exposed to ionising radiation (dose 3 Gy) by X-RAD 225XL instrument (Precision; Cu filter 0.5 mm). After 10 days, cells were collected and seeded on 12-well plates (10,000 cells/well) filled with semi-solid media containing 0.5% and 0.3% agar in bottom and upper layers, respectively. Plates were incubated for 8 weeks. The liquid media was changed 2 times per week for the duration of the experiment. Spheroid clones were picked under microscopic control, seeded in 96-well plate and expanded (referred to as RPE-PPM1D-T1-SA clones 1 to 6). Alternatively, BJ-hTert-HRASV12^ER-TAM^, BJ-hTert-HRASV12^ER-TAM^-PPM1D-T2, and BJ-hTert-HRASV12^ER-TAM^-PPM1D-T2-60 cells that survived 2-months continuous induction with 4OHT were cultured in semisolid media for 10 weeks.

### Flow cytometry

For determination the fraction of proliferating cells, BJ-hTert-HRASV12ER-TAM, BJ-hTert-HRASV12ER-TAM-PPM1D-T1 and BJ-hTert-HRASV12ER-TAM-PPM1D-T2 cells were treated with 4OHT for 5–20 days and were incubated with EdU 24 h before harvesting by trypsinization and fixation in 4% PFA. Cells were permeabilized with 0.5% Triton-X100 in PBS, washed and CLICK-IT reaction was performed using Alexa Azide 488. To determine the fraction of dead cells, cells were labelled by propidium iodide and DAPI and fraction of double positive cells was determined by flow cytometry.

### Cytogenetic analysis

RPE-wt, RPE-PPM1D-T1 cells and RPE-PPM1D-T1-SA clones were synchronised in mitosis by overnight incubation with colcemid (0.1 μg/ml). Multicolour fluorescence in situ hybridisation (mFISH) was performed using 24XCyte human multicolour FISH probe (MetaSystems) following a standard protocol. Karyotypes were analyzed using *IKAROS*/*ISIS* software (MetaSystems) and described according to ISCN 2020 nomenclature [76].

### Xenograft model

Mice (NOD.Cg-Prkdc^scid^ Il2rg^tm1Wjl^/SzJ aka NOD *scid* gamma) were maintained in the animal facility of the Institute of Molecular Genetics of the CAS. All animal experiments were approved by local ethical committee (project AVCR 2142-2022 SOV II). Suspension of RPE-wt, RPE-PPM1D-T1 and RPE-PPM1D-T1-SA clones (1.5 × 10^6^ cells) was injected subcutaneously in three mice under anaesthesia. Mice were sacrificed 4 weeks post-injection.

### DNA, RNA sequencing and analysis

DNA and RNA was isolated from asynchronously growing nontransformed RPE-PPM1D-T2 cells and six transformed RPE-PPM1D-T2-SA clones, which were all matched in terms of the days in cell culture, using Quick-gDNA Miniprep kit (Zymo Research) and RNeasy Mini Kit (Qiagen), respectively, following the manufacturer’s protocols. WES sequencing libraries (KAPA HyperExome Probes; Roche) were prepared as described previously using KAPA EvoPlus Kit (Roche) for DNA samples and KAPA RNA HyperPrep Kit (Roche) for RNA samples [77]. The final libraries were sequenced on the NovaSeq 6000 system using NovaSeq S1 Reagent Kit v1.5, 200 cycles (Illumina) with mean coverage >35 and >120 for DNA and RNA samples respectively. Bioinformatical analysis was performed as described previously [77]. Briefly, DNA fastq files were mapped to the hg19 reference using Novoalign (novoalign_2.08.03). PCR duplicates were removed from the BAM files using Picard Tools (picard-tools 1.129), and variant calling was performed using GATK HaplotypeCaller (3.8). Copy number variations (CNV) were analysed using CNVkit version 0.7.4. Areas with median coverage >20 were included in the analysis. RNA fastq files were mapped to the hg19 reference using STAR (STAR-2.5.2b). The PCR duplicates were removed using Picard Tools (picard-tools 1.129). All parts of RNAseq data analysis were conducted in R, version 4.3.2. [78], and RNAseq read counts were normalised using R package DESeq2 [79]. Fold change (FC) and log_2_FC were calculated from normalised reads, nontransformed RPE-PPM1D-T2 cells were considered a reference. Significance of differential expression for each gene was evaluated by Fisher’s t-test with simulated p values and Holm’s p value correction for multiple comparisons. Clustered heatmaps were plotted using R package pheatmap (https://CRAN.R-project.org/package=pheatmap). Given the nature of RNASeq data, Ward D2 was used for clustering with Manhattan distance function. Volcano plots were generated using in-house pipeline in R. In addition, gene set enrichment analysis (GSEA; including Molecular Signatures Database (MSigDB) for all Human Collections) using fgsea package in R was used to evaluate differences in gene expression using non-transformed RPE-PPM1D-T1 cells as reference [80]. Subsequently, we implemented the rrvgo (R-package reduce and visualise lists of GO; https://bioconductor.org/packages/release/bioc/html/rrvgo.html) to reduce complexity of the gene ontology (GO) terms derived from the largest collection of significant GO terms in C5 GO:BP (biological process ontology gene set) differentially enriched in individual clones [81].

### DNA fibre assay

Replication fork progression was determined as described previously [48]. Briefly, BJ-hTert-HRASV12^ER-TAM^ cells and their derivatives with truncated PPM1D were seeded in 6 well plates and incubated in presence or absence of GSK2830371 and 4-OHT for 5 days. Cells were pulsed with 5-chloro-2’-deoxyuridine (CldU, 30 μM, C6891, Merck) for 20 min, washed 3 times with PBS and then labelled with 5-iodo-2’-deoxyuridine (IdU, 250 μM, I7125, Merck) for 20 min. Cells were then trypsinized, collected in 750ul of media and centrifuged, 1200 rpm for for 5 min at 4 °C. Drop containing approx. 2500 cells was placed on a glass slide and lysis buffer (200 mM TrisHCl pH 7.4, 50 mM EDTA, 0,5% SDS) was applied for 9 min. The slides were then tilted at ~45° to allow the DNA from the lysed cells to spread. The slides were air-dried for 20 min and then fixed overnight in freshly prepared solution of methanol/acetic acid 3:1 at 4 °C. The DNA was then denatured in 2.5 M HCl for 1 h at room temperature. Slides were subsequently washed 3x in PBS and blocked in blocking solution (2% BSA, 0.1% Tween 20 in PBS, 0.22 μm filtered) for 40 min and then incubated with rat anti-CldU and mouse anti-IdU primary antibodies diluted in blocking solution for 2 h at room temperature. The slides were then washed 5x in 0.2% PBST and then dipped down 3x in blocking solution. After that, they were incubated with secondary antibodies diluted in blocking solution for 1 h at room temperature. The slides were then washed 5x in 0.2% PBST and then two times in PBS. Finally, the slides were air-dried at room temperature and a cover glass was mounted using Fluoromount-G mounting media. Measurement of the labelled DNA tracks was done in Image J.

### Microscopy

BJ-hTert HRASV12^ER-TAM^ cells and their derivatives were induced or not with 4OHT and/or PPM1D inhibitor for 5 to 20 days. Cells were then fixed in 4% PFA for 15 min, washed with PBS and stained for p16 and p-IRF3. For staining of heterochromatin markers (HP1, H3K9me3, 53BP1) cells were pre-extracted by incubation in 25 mM Hepes pH 7.7, 0.5% Triton X-100, 50 mM NaCl, 1 mM EDTA, 3 mM MgCl2, 300 mM sucrose for 5 min on ice prior to fixation. The images were acquired on Olympus ScanR high-throughput microscope equipped with a UPLFLN 60x/1.4 OIL objective and a motorised stage. Quantification was performed via the Olympus ScanR software. Alternatively, RPE cells were fixed 48 h after exposure to IR, stained with DAPI and imaged on Leica DM6000. Micronuclei were quantified using ImageJ by counting the total number of micronuclei and dividing them to the total number of cells of each condition. Images of cells growing in semi-solid media were acquired on Leica DMI8 using HC PL APO CS 10x objective. Quantification was done using ImageJ to measure the cell size. Statistical significance for all microscopy experiments was calculated by Student’s t-test. Senescence β-galactosidase assay was performed as described [82]. In brief, cells were fixed in 4% PFA, washed once with PBS and then stained with a β-gal staining solution (0.1% X-gal, 5 mM potassium ferrocyanide, 5 mM potassium ferricyanide, 150 mM sodium chloride, and 2 mM magnesium chloride in 40 mM citric acid/sodium phosphate solution, pH 6.0) overnight at 37 °C. After two washes with distilled water, cells were stained with DAPI, overlaid with 25% glycerol and imaged on Leica DM6000 microscope using HC PLAN APO 20x/0.70 DRY PH2 objective. Fraction of the β-gal positive cells were counted semi-automatically using the point tool in ImageJ.

### qPCR

cDNA was generated from RNA using random hexamer primers and RevertAid H Minus Reverse Transcriptase (Thermo Scientific) following the manufacturer’s protocol. The following primers were used for ISG54 (ACTGTGAGGAAGGGTGGACACGGT and AGCATGGAGGCTGGCAAGAATGGA), ISG56 (AGGCAGGCTGTCCGCTTAAATCCA and AGACGAACCCAAGGAGGCTCAAGC), ISG60 (CACTTGGGGAAAC-TACGCCTGGGT and GGCTGCACTGCGGAGGACATCTG), CDKN1A (GGCGGCAGACCAGCATGACA and CCTCGCGCTTCCAGGACTGC), NOXA (GCTGGGGAGAAACAGTTCAG and AATGTGCTGAGTTGGCACTG) and BAX (GCTGGACATTGGACTTCCTC and GTCTTGGATCCAGCCCAAC). qRT-PCR was performed using FastStart DNA Master SYBR Green I and LightCycler 480 II (Roche). The analysis was done using the ΔΔCT method and normalising to the GAPDH expression level.

### Statistical analysis

Unless stated otherwise, experiments were done in three biological replicates. Statistical significance was calculated by Student’s *t* test (*p ≤ 0.05, **p ≤ 0.01, ***p ≤ 0.001, ****p ≤ 0.0001) using GraphPad Prism v5 software.

## Supplementary information

Supplementary Figures S1-S11

Suppl. Table 1

Suppl. Table 2

Suppl. Table 3

## Data Availability

The complete data set from RNAseq analysis and sequencing of genomic DNA was deposited in ArrayExpress database (https://www.ebi.ac.uk/biostudies/arrayexpress) under accession numbers E-MTAB-13933 and E-MTAB-13923.
