# Supplementary material for: PPM1D activity promotes cellular transformation by preventing senescence and cell death
Source: Oncogene. 2024 Sep 5;43(42):3081–93. doi: 10.1038/s41388-024-03149-3 (PMC11473410; doi:10.1038/s41388-024-03149-3)
Supplement: Supplementary file 1 — Supplementary Figures S1-S11 [file 41388_2024_3149_MOESM1_ESM.pdf]

PPM1D activity promotes cellular transformation by preventing senescence and cell death

Miroslav Stoyanov, Andra S. Martinikova, Katerina Matejkova, Klara Horackova, Petra Zemankova, Kamila Burdova, Zuzana Zemanova, Petra Kleiblova, Zdenek Kleibl, Libor Macurek

Supplementary Figures S1-S11

Suppl. Fig. S1. Validation of a mouse monoclonal antibody to PPM1D

Suppl. Fig. S2. Activation of cGAS pathway in cells with truncated PPM1D after exposure to IR

Suppl. Fig. S3. Karyotyping of the transformed RPE-PPM1D-T2-SA cells

Suppl. Fig. S4. Volcano plots from transcriptomic analysis of the transformed RPE-PPM1D-T2 cells

Suppl. Fig. S5. Clustering analysis of the parental and transformed RPE-PPM1D-T2 cells

Suppl. Fig. S6. Transformed RPE-PPM1D-T2-SA clones remain p53 proficient

Suppl. Fig. S7. Similarity metrics heat maps for the transformed RPE-PPM1D-T2-SA clones

Suppl. Fig. S8. Treemap plots for the transformed RPE-PPM1D-T2-SA clones

Suppl. Fig. S9. Significantly enriched parental GO:BP terms in individual RPE-PPM1D-T2-SA clones

Suppl. Fig. S10. Truncated PPM1D promotes override of OIS in cells expressing active RAS

Suppl. Fig. S11. Senescence induced by RAS is p53 dependent

Legend to Supplementary Tables 1-3

Suppl. Table 1. Expression profiling of the transformed RPE-PPM1D-T2-SA clones

Suppl. Table 2. Genotypes of the transformed RPE-PPM1D-T2-SA clones

Suppl. Table 3. GSEA analysis comparing individual RPE-PPM1D-T2-SA clones with parental cells

Suppl. Fig. S1. Validation of a mouse monoclonal antibody to PPM1D

A) Whole cell lysates from parental RPE, RPE-PPM1D-T1 and RPE-PPM1D-T2 cells were probed with the custom-made mouse monoclonal PPM1D (clone 5) antibody. **B)** Parental U2OS and U2OS-PPM1D-KO cells lacking PPM1D were probed with PPM1D (clone 5) antibody. Note that U2OS cells contain the wild type and mutant allele of *PPM1D* leading to expression of the full-length and C-terminally truncated PPM1D. **C)** Parental U2OS and U2OS-PPM1D-KO cells were fixed by PFA, stained with PPM1D (clone 5) antibody (2 μg/mL) and imaged by confocal microscopy. **D)** MCF7 cells treated or not with PPM1Di were fixed by PFA, stained with PPM1D (clone 5) antibody and imaged by confocal microscopy. Note that treatment with PPM1Di leads to strong reduction of PPM1D levels in cells, confirming the specificity of the observed signal. **E)** HEK293 cells were transfected with plasmid expressing the mouse PPM1D and reactivity of the commercial PPM1D (Santa Cruz) and custom made PPM1D (clone 5) antibodies was compared by immunoblotting. **F)** Epitope mapping of the PPM1D (clone 5) antibody using PepSpot peptides immobilized on nitrocellulose membrane. The epitope was mapped to aa 385-399 (FTNEDELYLNLTDSP) of human PPM1D. **G)** U2OS-PPM1D-KO cells were transfected with full length EGFP-PPM1D or mutant lacking AA380-400 and reactivity of PPM1D (clone 5) antibody was tested using immunoblotting.

Supplementary Fig. 1

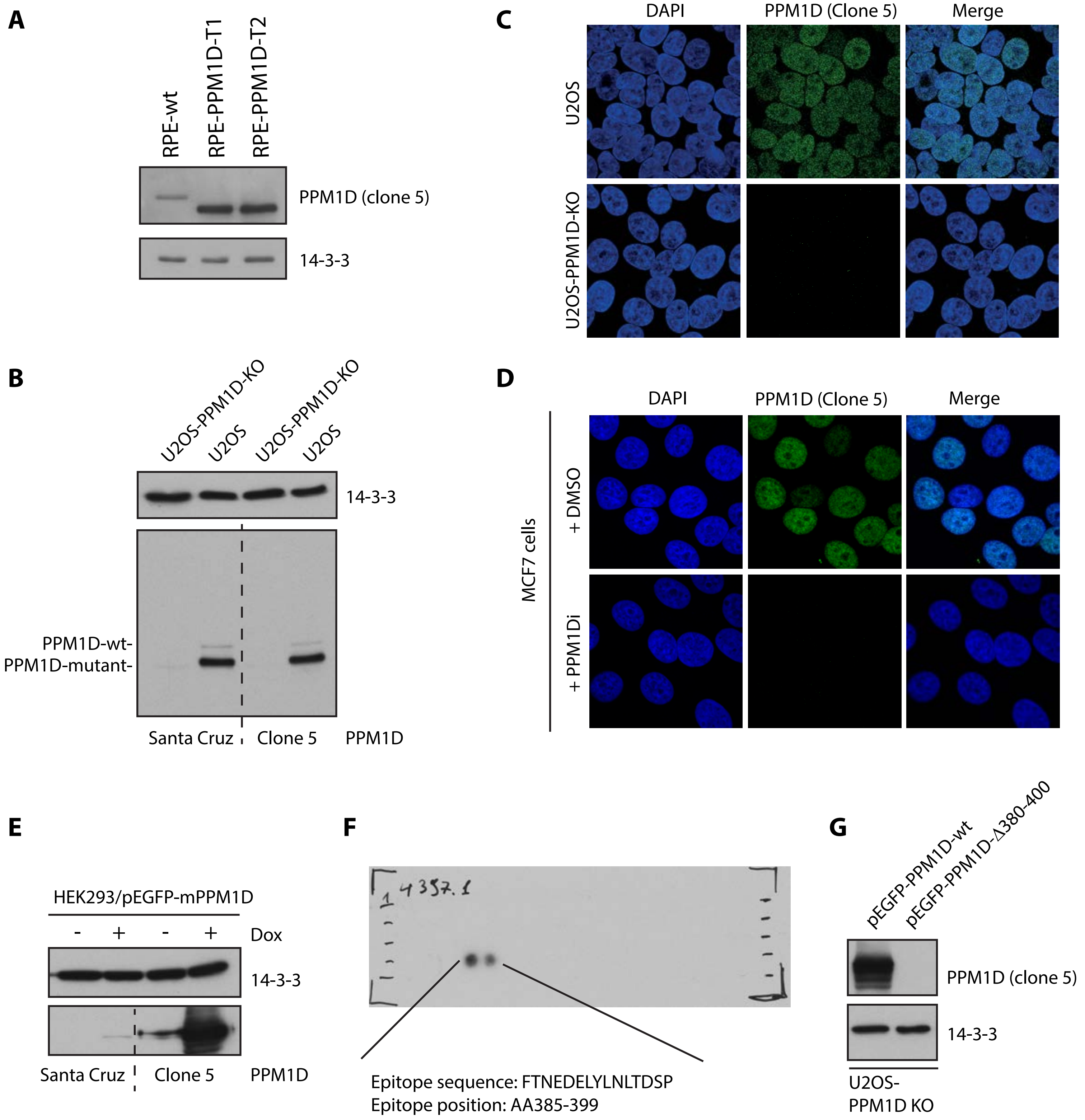

Suppl. Fig. S2. Activation of cGAS pathway in cells with truncated PPM1D after exposure to IR

A) Parental RPE and RPE-PPM1D-T2 stably transfected with RFP-cGAS were mock treated or irradiated (3 Gy) in the presence or absence of PPM1Di, fixed after 48 h and probed for IRF3-pS386 by ScanR microscopy. Representative images are shown. **B)** Quantification of IRF3-pS386 signal from A. Each dot represents a single cell. Red bar indicates median. Statistical significance was evaluated by Student's t-test (*** $p \leq 0.001$).

Supplementary Fig. 2

A

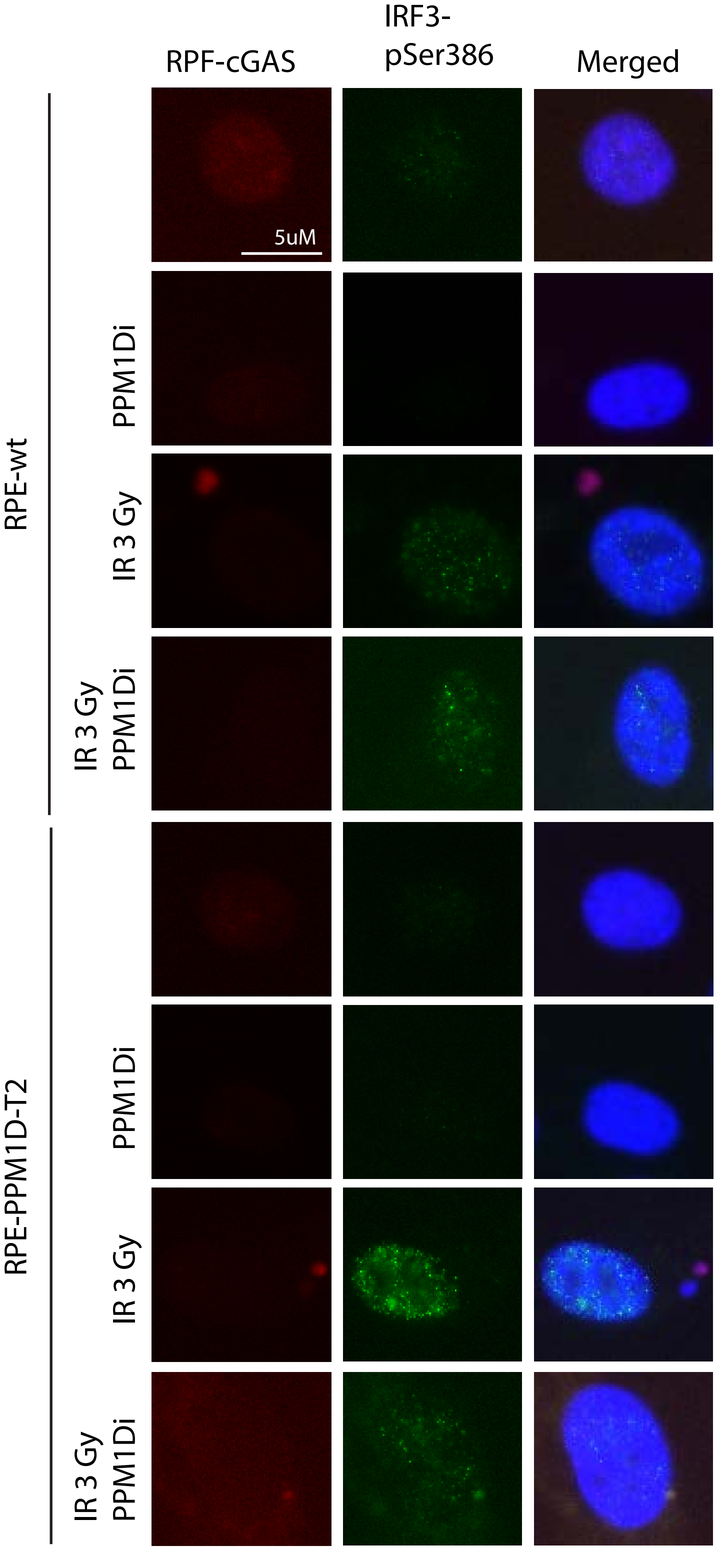

B

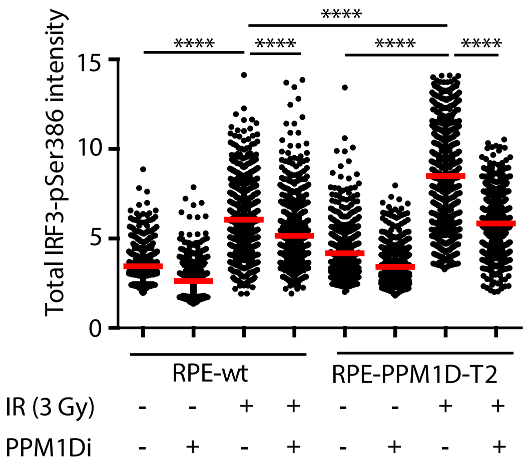

Suppl. Fig. S3. Karyotyping of the transformed RPE-PPM1D-T2-SA cells

A) Parental RPE-PPM1D-T2 cells and transformed RPE-PPM1D-T2-SA (clones 2 to 5) were arrested in mitosis, fixed and probed by M-FISH. **B)** Karyotyping of the parental RPE-PPM1D-T2 and all transformed RPE-PPM1D-T2-SA clones.

Suppl. Fig. S3

A

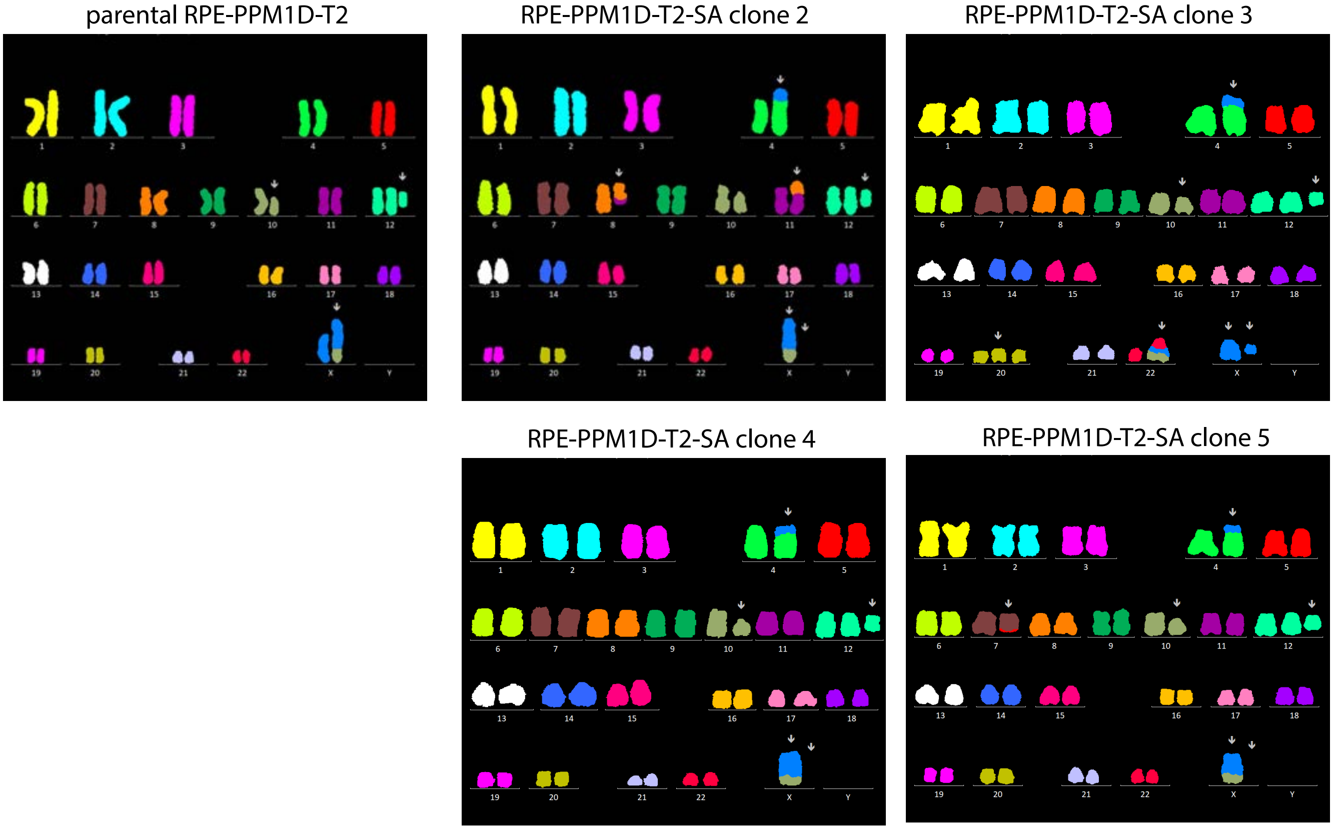

B

Cells	Karyotype
RPE-PPM1D-T2-SA clone 1	40~46,-X,der(X)t(X;7)(q13;q?),der(4)t(X;4)(?;p15),der(7)t(X;7)(q13;q11.23)t(X;10)(q28;q21.2),del(10)(p11.1),+i(12)(p10)[cp14]
RPE-PPM1D-T2-SA clone 2	40~46,-X,der(X)t(X;10)(q28;q21.2),der(4)t(X;4)(?;p15),t(8;11)(q13;p12),del(10)(p11.1),+i(12)(p10)[cp9]
RPE-PPM1D-T2-SA clone 3	42~48,del(X)(p11.1),r(X)(p11.1q13),der(4)t(X;4)(?;p15),del(10)(p11.1),+i(12)(p10),+20,der(22)t(X;22)(q13;q13)t(X;10)(q28;q21.2)[cp15]
RPE-PPM1D-T2-SA clone 4	40~46,-X,der(X)t(X;10)(q28;q21.2),der(4)t(X;4)(?;p15),del(10)(p11.1),+i(12)(p10)[cp24]
RPE-PPM1D-T2-SA clone 5	40~46,-X,der(X)t(X;10)(q28;q21.2),der(4)t(X;4)(?;p15),der(7)t(5;7)(?;q11.23),del(10)(p11.1),+i(12)(p10)[cp12]
RPE-PPM1D-T2-SA clone 6	41~46,-X,der(X)t(X;10)(q28;p11.2),t(2;11)(p13;p15),t(2;5)(p12;p11),der(4)t(X;4)(?;p15),ins(10;X)(p11.1;?),+i(12)(p10)[cp12]
parental RPE-PPM1D-T2 cells	33~47,X,der(X)t(X;10)(q28;q21.2),del(10)(p11.1),+i(12)(p10)[cp24]

Suppl. Fig. S4. Volcano plots from transcriptomic analysis of the transformed RPE-PPM1D-T2 cells

Volcano plots showing the differential expression of transcripts in the transformed RPE-PPM1D-T2-SA clones 1-6 compared to the parental RPE-PPM1D-T2 cells. Cut-off indicated by dashed lines was set to $p_{adj} < 0.000001$ and $FC > 3$ or $FC < 0.3$ for upregulated and downregulated genes, respectively. Significantly upregulated genes are shown in red, downregulated in blue.

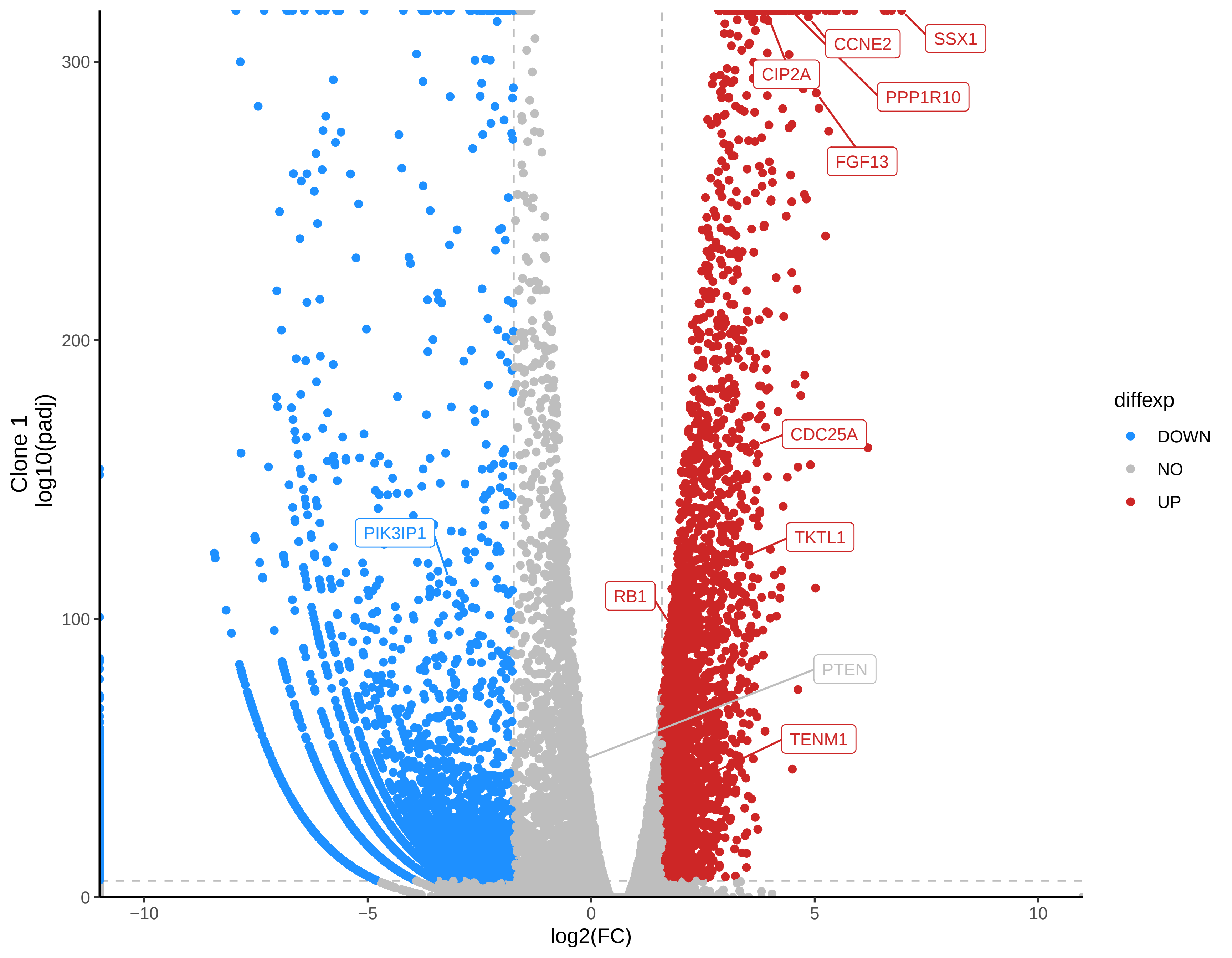
SSX1
CCNE2
CIP2A
PPP1R10
FGF13
CDC25A
TKTL1
RB1
PTEN
TENM1
PIK3IP1
300
200
100
0
Clone 1
log10(padj)
−10
−5
0
5
10
log2(FC)
diffexp
DOWN
NO
UP

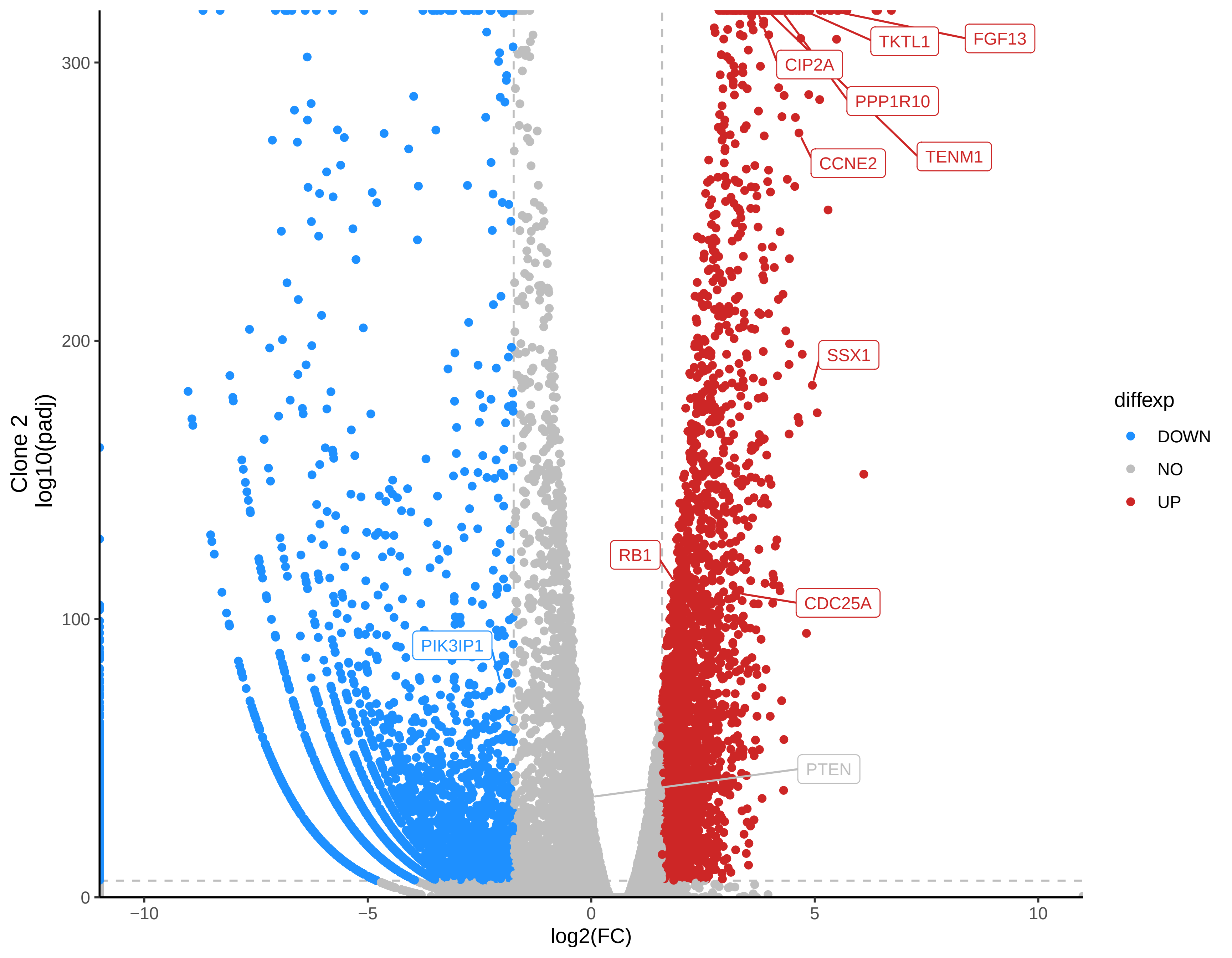

Clone 2
log10(padj)
300
200
100
0
−10
−5
0
5
10
log2(FC)
TKTL1
FGF13
CIP2A
PPP1R10
TENM1
CCNE2
SSX1
RB1
CDC25A
PIK3IP1
PTEN
diffexp
DOWN
NO
UP

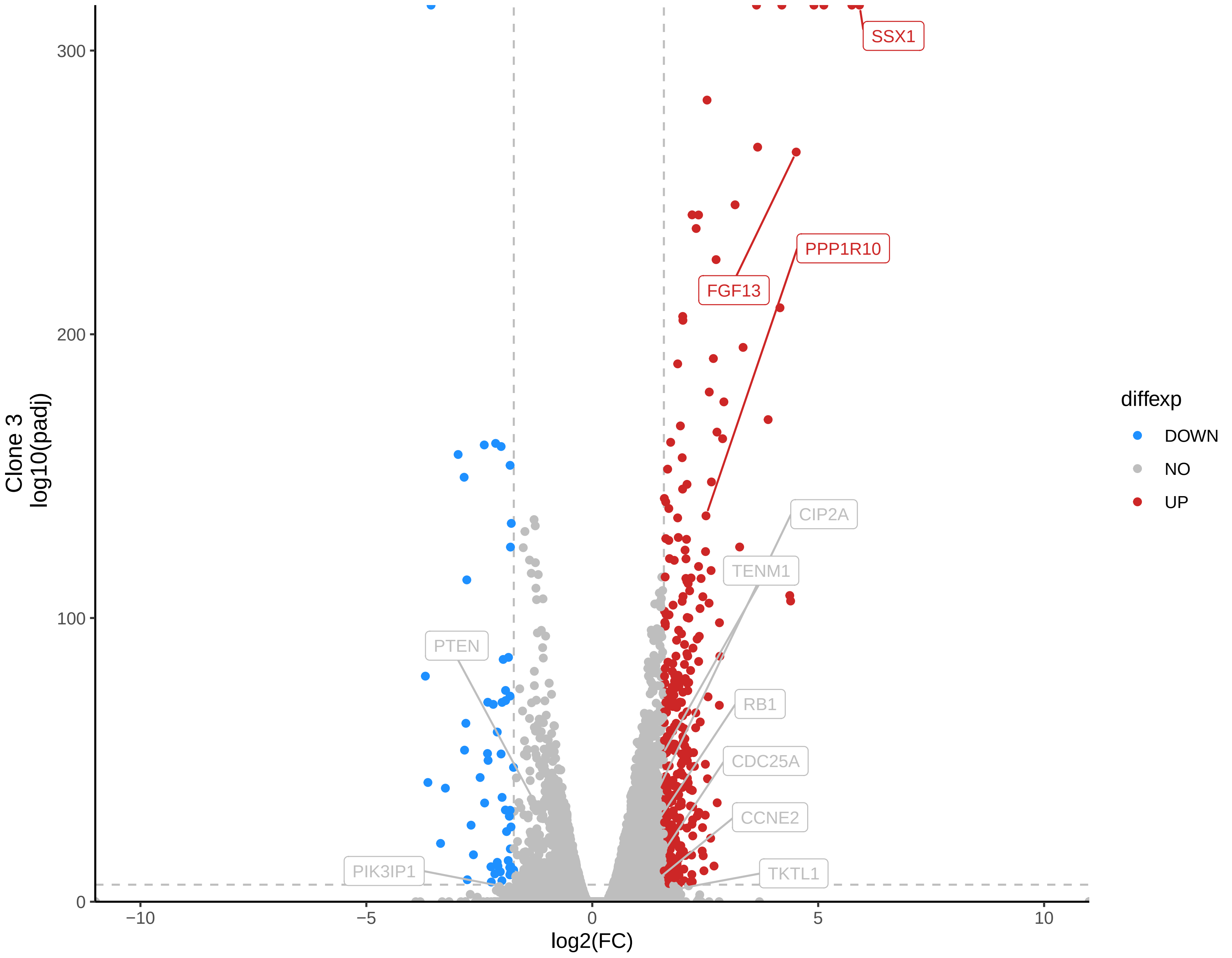

Clone 3
log10(padj)
log2(FC)
diffexp
DOWN
NO
UP
SSX1
PPP1R10
FGF13
CIP2A
TENM1
PTEN
RB1
CDC25A
CCNE2
PIK3IP1
TKTL1
300
200
100
0
−10
−5
0
5
10

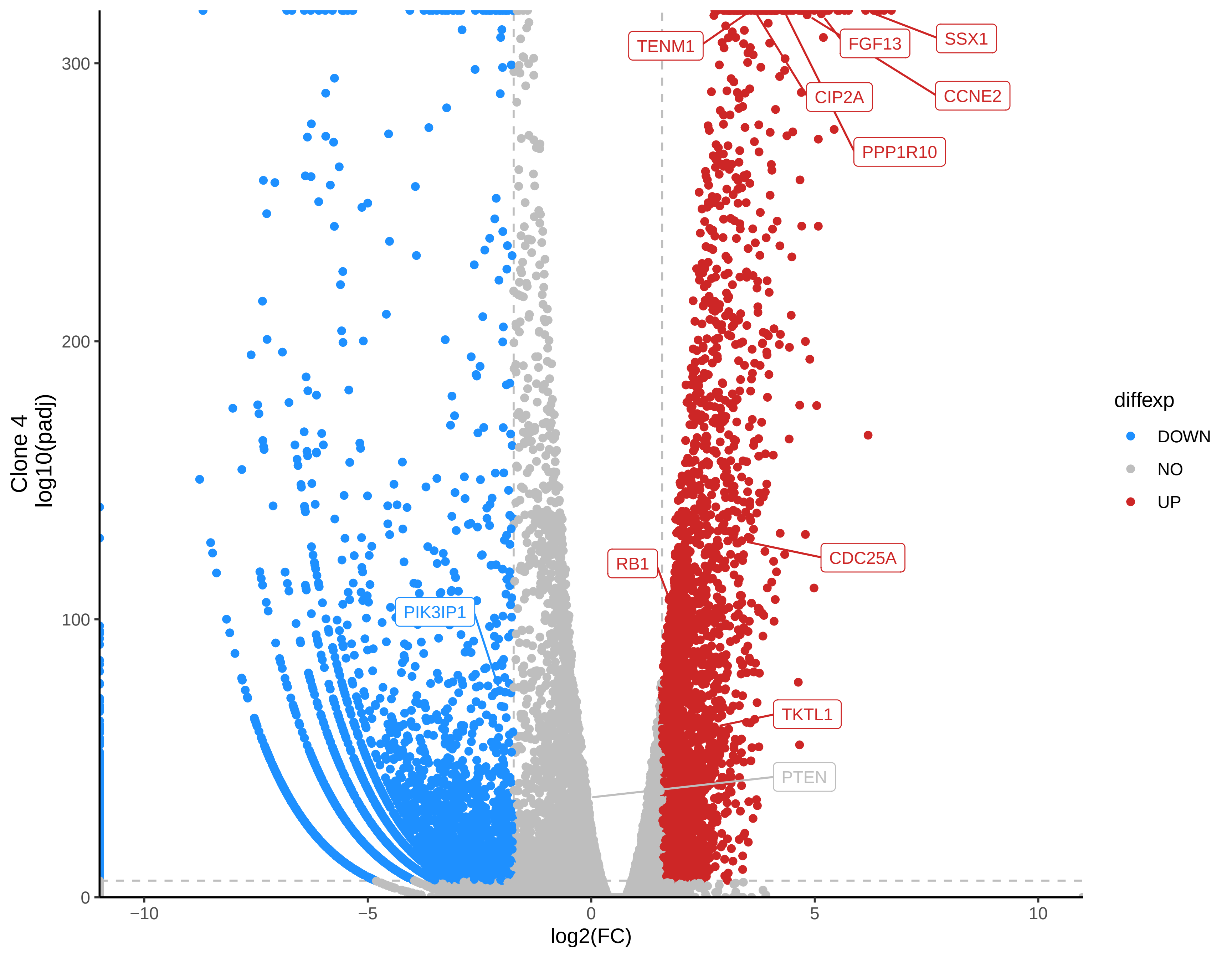
TENM1
FGF13
SSX1
CIP2A
CCNE2
PPP1R10
RB1
CDC25A
PIK3IP1
TKTL1
PTEN
300
200
100
0
−10
−5
0
5
10
Clone 4
log10(padj)
log2(FC)
diffexp
DOWN
NO
UP

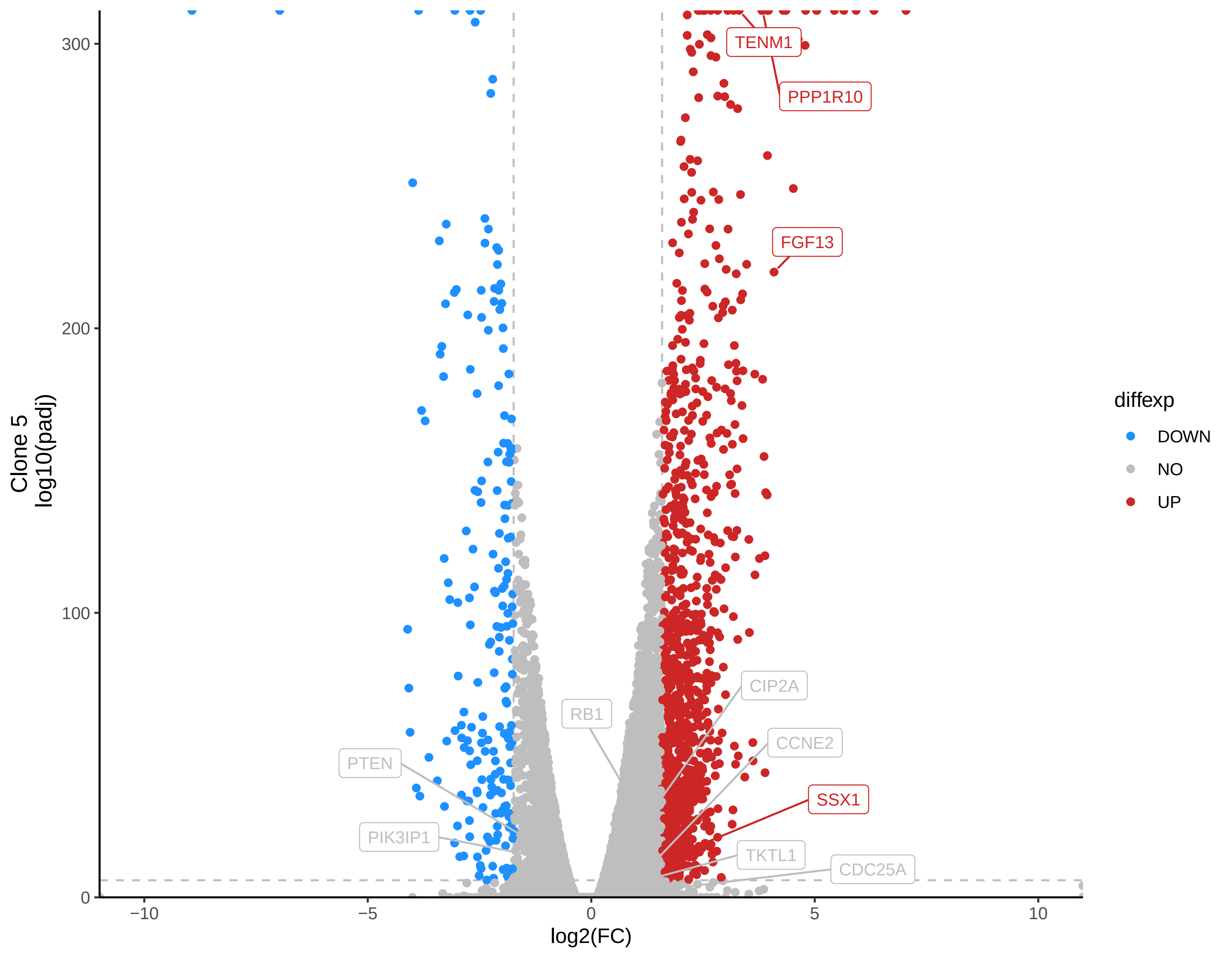

Clone 5
log10(padj)
log2(FC)
300
200
100
0
−10
−5
0
5
10
TENM1
PPP1R10
FGF13
CIP2A
RB1
CCNE2
PTEN
SSX1
PIK3IP1
TKTL1
CDC25A
diffexp
DOWN
NO
UP

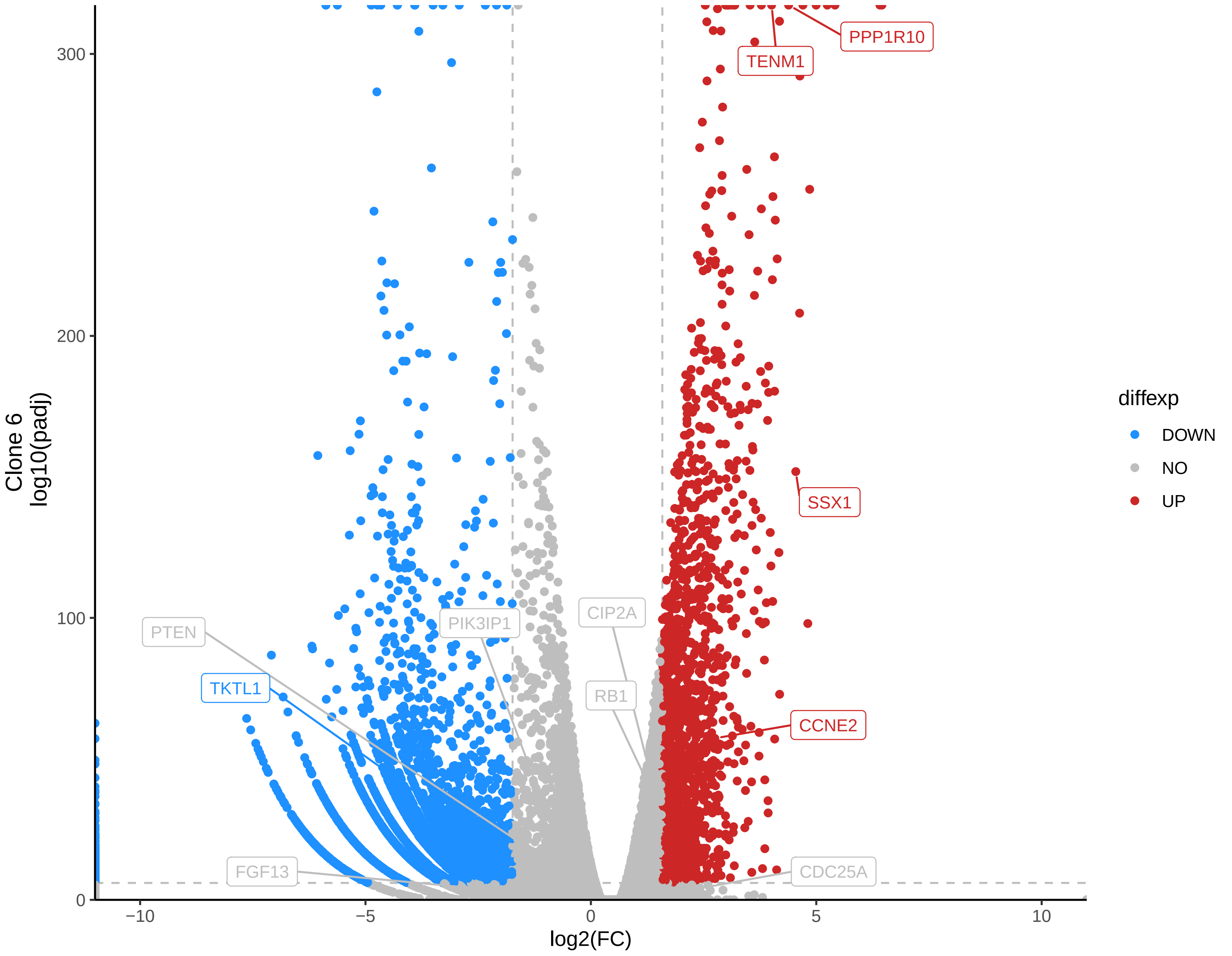

Clone 6
log10(padj)
log2(FC)
300
200
100
0
−10
−5
0
5
10
PPP1R10
TENM1
SSX1
CCNE2
CDC25A
CIP2A
RB1
PIK3IP1
PTEN
TKTL1
FGF13
diffexp
DOWN
NO
UP

Suppl. Fig. S5. Clustering analysis of the parental and transformed RPE-PPM1D-T2 cells

Results of clustering analysis are depicted in heatmap with low expressed genes coloured blue and highly expressed genes coloured red. Logarithmized counts normalized with DESeq were used for the calculations. Ward.D2 algorithm was used as clustering method and Manhattan algorithm was used to measure distance. Transcription profiles of clones 1, 4 and 6 of the transformed RPE-PPM1D-T2 cells create one cluster, whereas clones 3 and 5 create another cluster with their transcription profiles more closely resembling the transcription profile of the parental RPE-PPM1D-T2 cells (depicted as clone 0).

Supplementary Fig. S5.

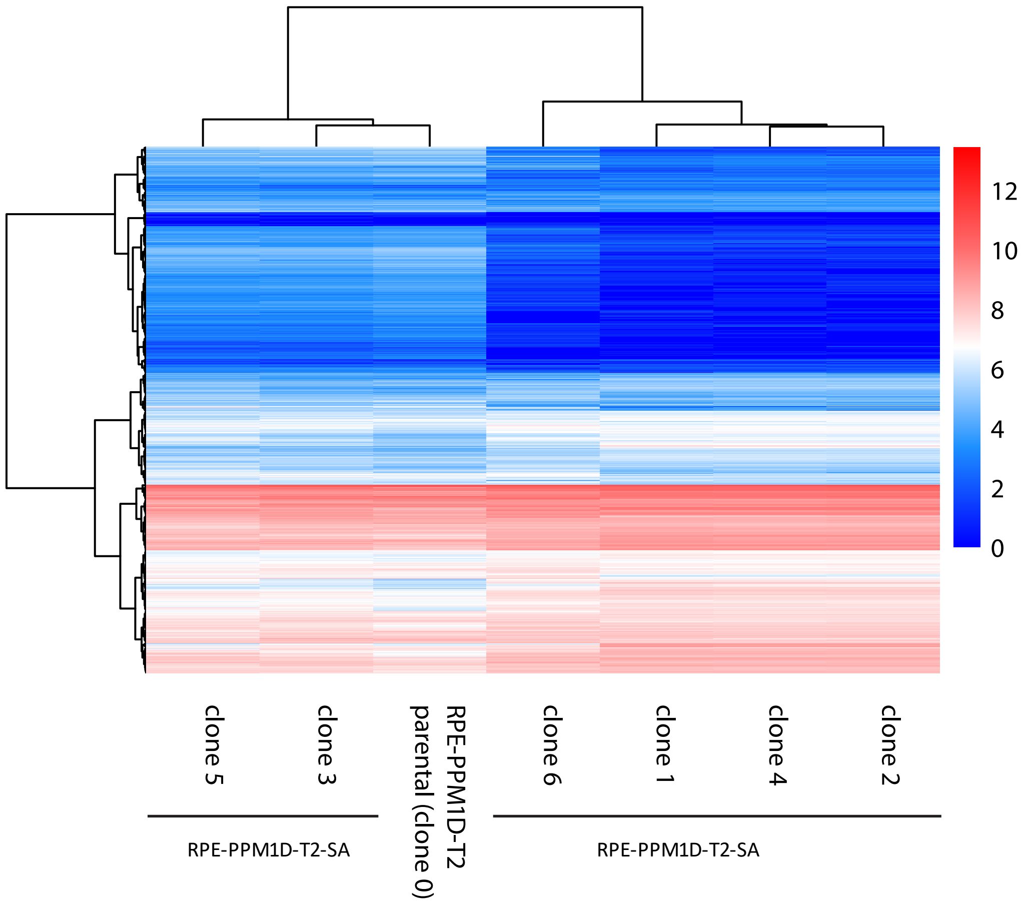

Suppl. Fig. S6. Transformed RPE-PPM1D-T2-SA clones remain p53 proficient

Parental RPE, RPE-PPM1D-T2 and transformed RPE-PPM1D-T2-SA cells were exposed or not to ionising radiation. Expression of indicated p53 target genes was evaluated by qPCR. Statistical significance was determined by t-test, error bars indicate SD, n=3.

Supplementary Fig. 6

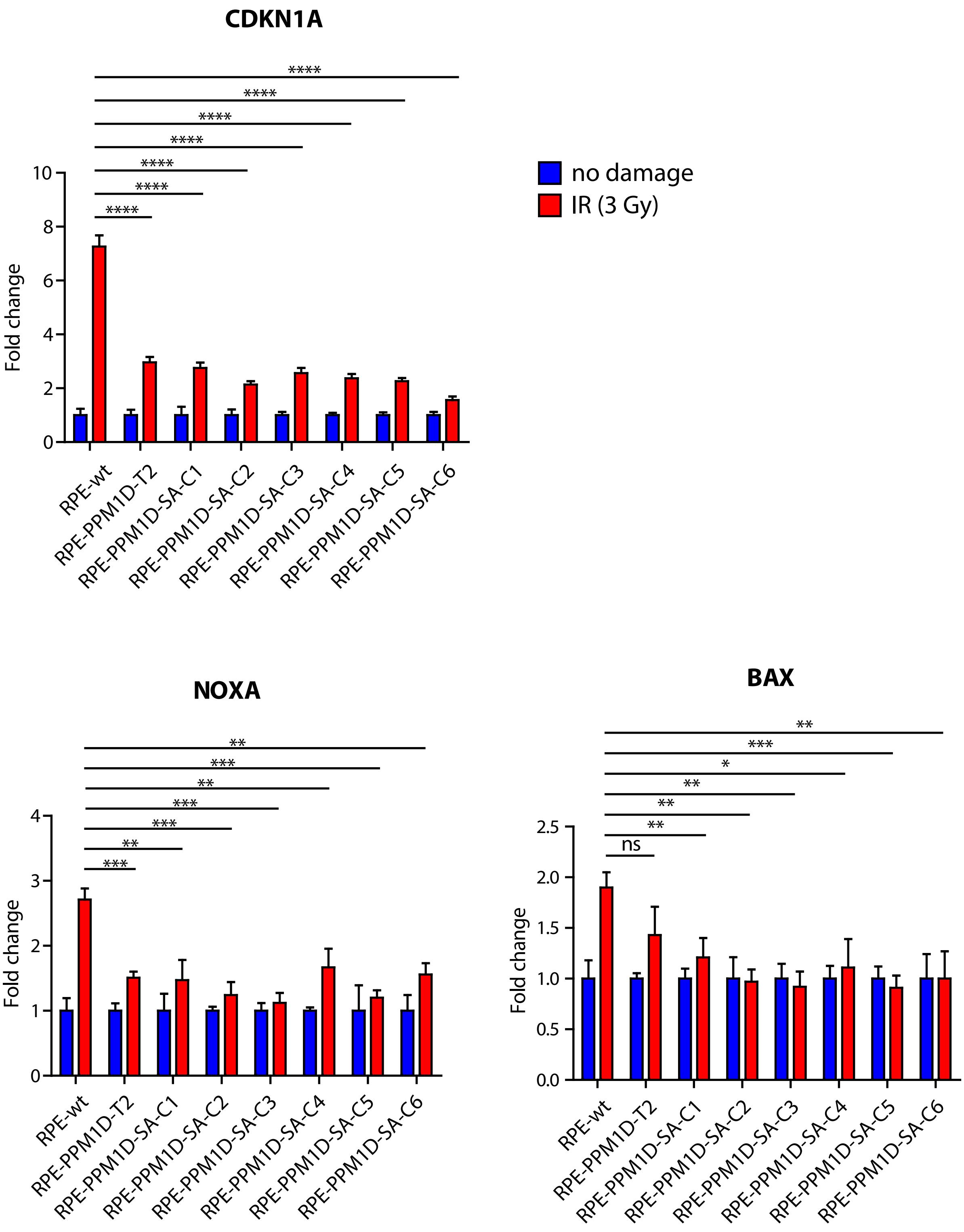

Suppl. Fig. S7. Similarity metrics heat maps for the transformed RPE-PPM1D-T2-SA clones

Similarity metrics heat maps generated by the rrvgo package aggregate related GO:BP (gene ontology: biological functions) terms enriched significantly from the GSEA analysis (padj<0.05; Suppl Table 3). Clone #6 is not shown as it showed no significant GO:BP enrichment.

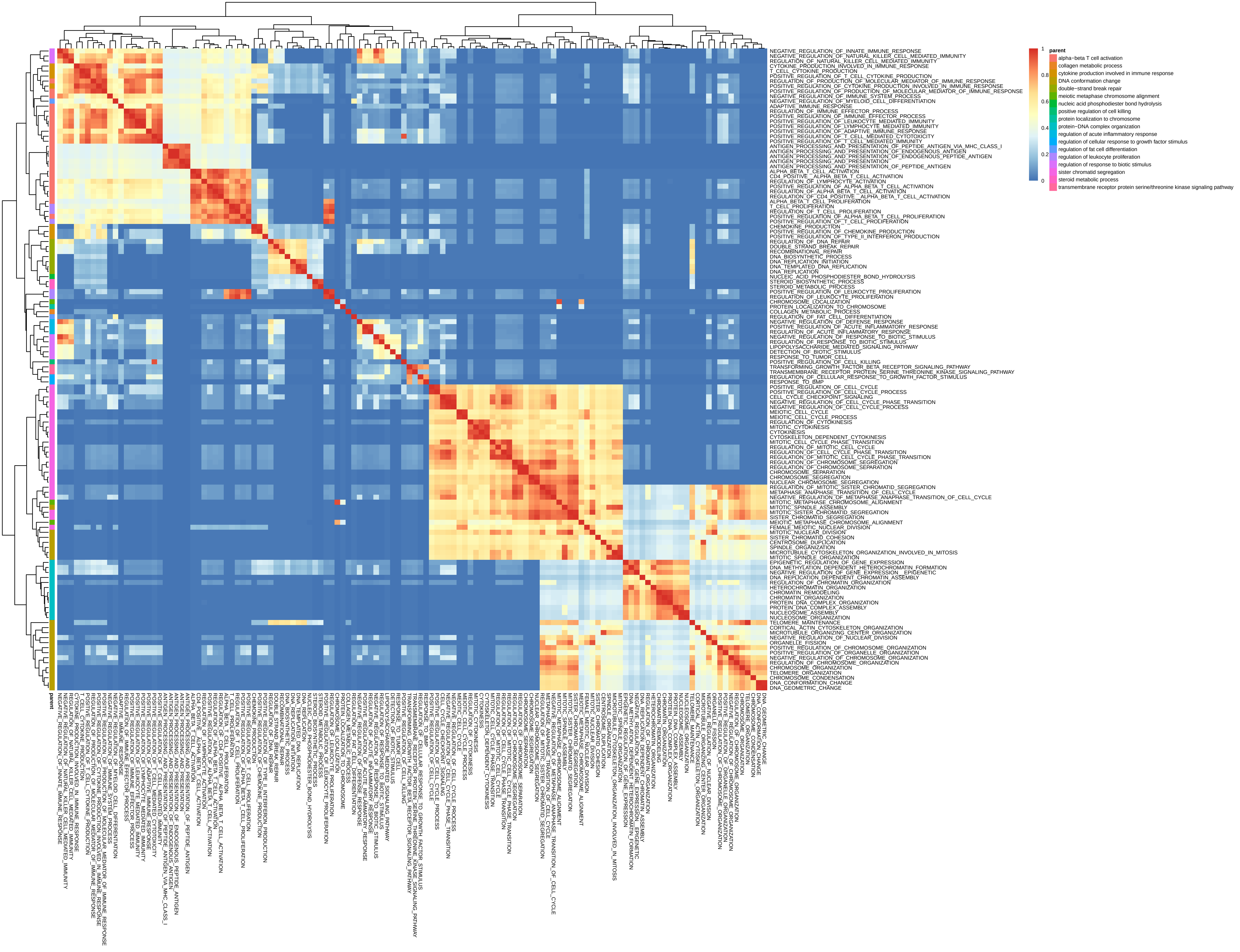
NEGATIVE_REGULATION_OF_INNATE_IMMUNE_RESPONSE
NEGATIVE_REGULATION_OF_NATURAL_KILLER_CELL_MEDIATED_IMMUNITY
REGULATION_OF_NATURAL_KILLER_CELL_MEDIATED_IMMUNITY
CYTOKINE_PRODUCTION_INVOLVED_IN_IMMUNE_RESPONSE
T_CELL_CYTOKINE_PRODUCTION
POSITIVE_REGULATION_OF_T_CELL_CYTOKINE_PRODUCTION
REGULATION_OF_PRODUCTION_OF_MOLECULAR_MEDIATOR_OF_IMMUNE_RESPONSE
POSITIVE_REGULATION_OF_CYTOKINE_PRODUCTION_INVOLVED_IN_IMMUNE_RESPONSE
POSITIVE_REGULATION_OF_PRODUCTION_OF_MOLECULAR_MEDIATOR_OF_IMMUNE_RESPONSE
NEGATIVE_REGULATION_OF_IMMUNE_SYSTEM_PROCESS
NEGATIVE_REGULATION_OF_MYELOID_CELL_DIFFERENTIATION
ADAPTIVE_IMMUNE_RESPONSE
REGULATION_OF_IMMUNE_EFFECTOR_PROCESS
POSITIVE_REGULATION_OF_IMMUNE_EFFECTOR_PROCESS
POSITIVE_REGULATION_OF_LEUKOCYTE_MEDIATED_IMMUNITY
POSITIVE_REGULATION_OF_LYMPHOCYTE_MEDIATED_IMMUNITY
POSITIVE_REGULATION_OF_ADAPTIVE_IMMUNE_RESPONSE
POSITIVE_REGULATION_OF_T_CELL_MEDIATED_CYTOTOXICITY
POSITIVE_REGULATION_OF_T_CELL_MEDIATED_IMMUNITY
ANTIGEN_PROCESSING_AND_PRESENTATION_OF_PEPTIDE_ANTIGEN_VIA_MHC_CLASS_I
ANTIGEN_PROCESSING_AND_PRESENTATION_OF_ENDOGENOUS_ANTIGEN
ANTIGEN_PROCESSING_AND_PRESENTATION_OF_ENDOGENOUS_PEPTIDE_ANTIGEN
ANTIGEN_PROCESSING_AND_PRESENTATION
ANTIGEN_PROCESSING_AND_PRESENTATION_OF_PEPTIDE_ANTIGEN
ALPHA_BETA_T_CELL_ACTIVATION
CD4_POSITIVE__ALPHA_BETA_T_CELL_ACTIVATION
REGULATION_OF_LYMPHOCYTE_ACTIVATION
POSITIVE_REGULATION_OF_ALPHA_BETA_T_CELL_ACTIVATION
REGULATION_OF_ALPHA_BETA_T_CELL_ACTIVATION
REGULATION_OF_CD4_POSITIVE__ALPHA_BETA_T_CELL_ACTIVATION
ALPHA_BETA_T_CELL_PROLIFERATION
T_CELL_PROLIFERATION
REGULATION_OF_T_CELL_PROLIFERATION
POSITIVE_REGULATION_OF_ALPHA_BETA_T_CELL_PROLIFERATION
POSITIVE_REGULATION_OF_T_CELL_PROLIFERATION
CHEMOKINE_PRODUCTION
POSITIVE_REGULATION_OF_CHEMOKINE_PRODUCTION
POSITIVE_REGULATION_OF_TYPE_II_INTERFERON_PRODUCTION
REGULATION_OF_DNA_REPAIR
DOUBLE_STRAND_BREAK_REPAIR
RECOMBINATIONAL_REPAIR
DNA_BIOSYNTHETIC_PROCESS
DNA_REPLICATION_INITIATION
DNA_TEMPLATED_DNA_REPLICATION
DNA_REPLICATION
NUCLEIC_ACID_PHOSPHODIESTER_BOND_HYDROLYSIS
STEROID_BIOSYNTHETIC_PROCESS
STEROID_METABOLIC_PROCESS
POSITIVE_REGULATION_OF_LEUKOCYTE_PROLIFERATION
REGULATION_OF_LEUKOCYTE_PROLIFERATION
CHROMOSOME_LOCALIZATION
PROTEIN_LOCALIZATION_TO_CHROMOSOME
COLLAGEN_METABOLIC_PROCESS
REGULATION_OF_FAT_CELL_DIFFERENTIATION
NEGATIVE_REGULATION_OF_DEFENSE_RESPONSE
POSITIVE_REGULATION_OF_ACUTE_INFLAMMATORY_RESPONSE
REGULATION_OF_ACUTE_INFLAMMATORY_RESPONSE
NEGATIVE_REGULATION_OF_RESPONSE_TO_BIOTIC_STIMULUS
REGULATION_OF_RESPONSE_TO_BIOTIC_STIMULUS
LIPOPOLYSACCHARIDE_MEDIATED_SIGNALING_PATHWAY
DETECTION_OF_BIOTIC_STIMULUS
RESPONSE_TO_TUMOR_CELL
POSITIVE_REGULATION_OF_CELL_KILLING
TRANSFORMING_GROWTH_FACTOR_BETA_RECEPTOR_SIGNALING_PATHWAY
TRANSMEMBRANE_RECEPTOR_PROTEIN_SERINE_THREONINE_KINASE_SIGNALING_PATHWAY
REGULATION_OF_CELLULAR_RESPONSE_TO_GROWTH_FACTOR_STIMULUS
RESPONSE_TO_BMP
POSITIVE_REGULATION_OF_CELL_CYCLE
POSITIVE_REGULATION_OF_CELL_CYCLE_PROCESS
CELL_CYCLE_CHECKPOINT_SIGNALING
NEGATIVE_REGULATION_OF_CELL_CYCLE_PHASE_TRANSITION
NEGATIVE_REGULATION_OF_CELL_CYCLE_PROCESS
MEIOTIC_CELL_CYCLE
MEIOTIC_CELL_CYCLE_PROCESS
REGULATION_OF_CYTOKINESIS
MITOTIC_CYTOKINESIS
CYTOKINESIS
CYTOSKELETON_DEPENDENT_CYTOKINESIS
MITOTIC_CELL_CYCLE_PHASE_TRANSITION
REGULATION_OF_MITOTIC_CELL_CYCLE
REGULATION_OF_CELL_CYCLE_PHASE_TRANSITION
REGULATION_OF_MITOTIC_CELL_CYCLE_PHASE_TRANSITION
REGULATION_OF_CHROMOSOME_SEGREGATION
REGULATION_OF_CHROMOSOME_SEPARATION
CHROMOSOME_SEPARATION
CHROMOSOME_SEGREGATION
NUCLEAR_CHROMOSOME_SEGREGATION
REGULATION_OF_MITOTIC_SISTER_CHROMATID_SEGREGATION
METAPHASE_ANAPHASE_TRANSITION_OF_CELL_CYCLE
NEGATIVE_REGULATION_OF_METAPHASE_ANAPHASE_TRANSITION_OF_CELL_CYCLE
MITOTIC_METAPHASE_CHROMOSOME_ALIGNMENT
MITOTIC_SPINDLE_ASSEMBLY
MITOTIC_SISTER_CHROMATID_SEGREGATION
SISTER_CHROMATID_SEGREGATION
MEIOTIC_METAPHASE_CHROMOSOME_ALIGNMENT
FEMALE_MEIOTIC_NUCLEAR_DIVISION
MITOTIC_NUCLEAR_DIVISION
SISTER_CHROMATID_COHESION
CENTROSOME_DUPLICATION
SPINDLE_ORGANIZATION
MICROTUBULE_CYTOSKELETON_ORGANIZATION_INVOLVED_IN_MITOSIS
MITOTIC_SPINDLE_ORGANIZATION
EPIGENETIC_REGULATION_OF_GENE_EXPRESSION
DNA_METHYLATION_DEPENDENT_HETEROCHROMATIN_FORMATION
NEGATIVE_REGULATION_OF_GENE_EXPRESSION__EPIGENETIC
DNA_REPLICATION_DEPENDENT_CHROMATIN_ASSEMBLY
REGULATION_OF_CHROMATIN_ORGANIZATION
HETEROCHROMATIN_ORGANIZATION
CHROMATIN_REMODELING
CHROMATIN_ORGANIZATION
PROTEIN_DNA_COMPLEX_ORGANIZATION
PROTEIN_DNA_COMPLEX_ASSEMBLY
NUCLEOSOME_ASSEMBLY
NUCLEOSOME_ORGANIZATION
TELOMERE_MAINTENANCE
CORTICAL_ACTIN_CYTOSKELETON_ORGANIZATION
MICROTUBULE_ORGANIZING_CENTER_ORGANIZATION
NEGATIVE_REGULATION_OF_NUCLEAR_DIVISION
ORGANELLE_FISSION
POSITIVE_REGULATION_OF_CHROMOSOME_ORGANIZATION
POSITIVE_REGULATION_OF_ORGANELLE_ORGANIZATION
NEGATIVE_REGULATION_OF_CHROMOSOME_ORGANIZATION
REGULATION_OF_CHROMOSOME_ORGANIZATION
CHROMOSOME_ORGANIZATION
TELOMERE_ORGANIZATION
CHROMOSOME_CONDENSATION
DNA_CONFORMATION_CHANGE
DNA_GEOMETRIC_CHANGE
parent
1
0.8
0.6
0.4
0.2
0
parent
alpha−beta T cell activation
collagen metabolic process
cytokine production involved in immune response
DNA conformation change
double−strand break repair
meiotic metaphase chromosome alignment
nucleic acid phosphodiester bond hydrolysis
positive regulation of cell killing
protein localization to chromosome
protein−DNA complex organization
regulation of acute inflammatory response
regulation of cellular response to growth factor stimulus
regulation of fat cell differentiation
regulation of leukocyte proliferation
regulation of response to biotic stimulus
sister chromatid segregation
steroid metabolic process
transmembrane receptor protein serine/threonine kinase signaling pathway

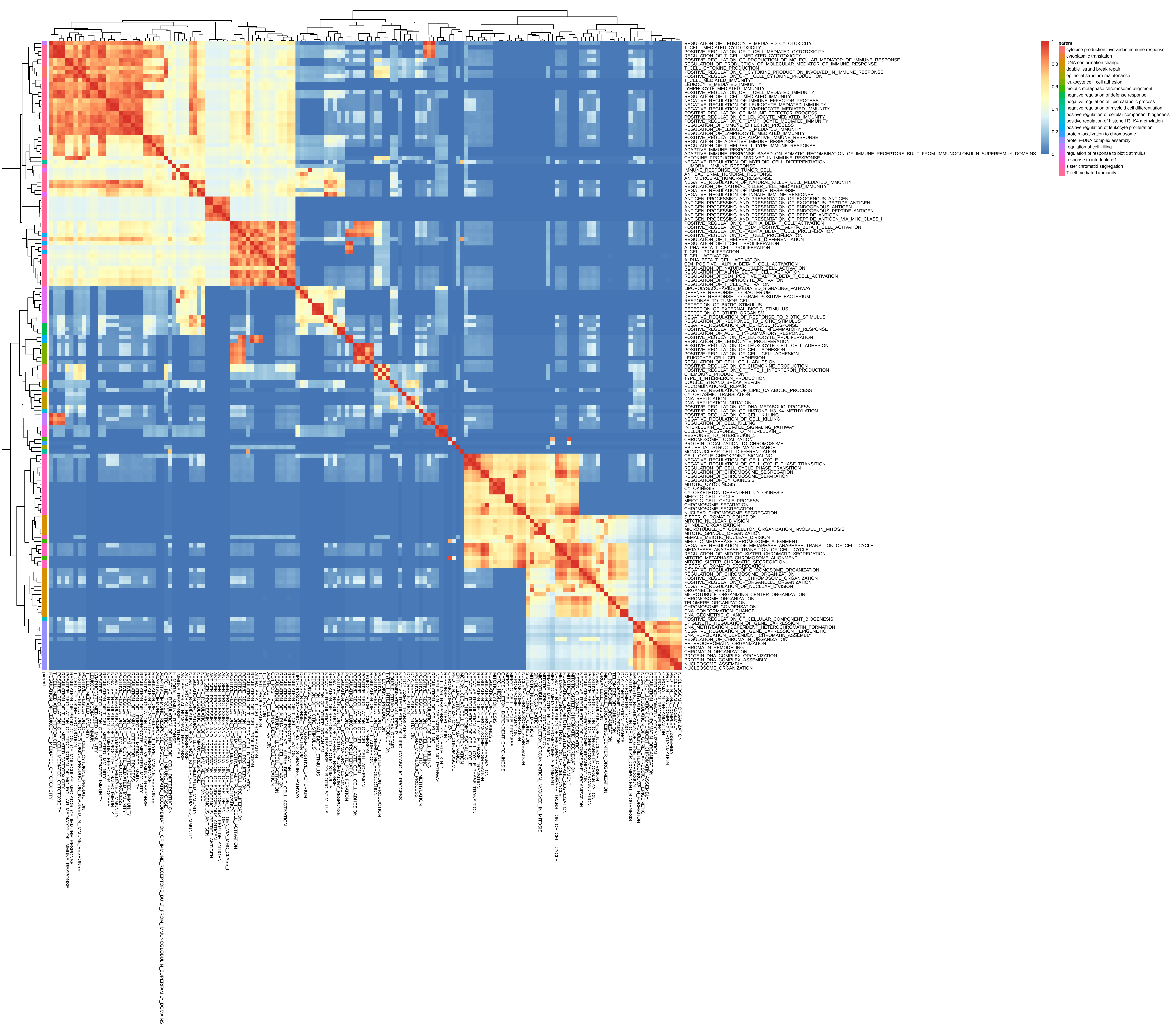

parent
cytokine production involved in immune response
cytoplasmic translation
DNA conformation change
double−strand break repair
epithelial structure maintenance
leukocyte cell−cell adhesion
meiotic metaphase chromosome alignment
negative regulation of defense response
negative regulation of lipid catabolic process
negative regulation of myeloid cell differentiation
positive regulation of cellular component biogenesis
positive regulation of histone H3−K4 methylation
positive regulation of leukocyte proliferation
protein localization to chromosome
protein−DNA complex assembly
regulation of cell killing
regulation of response to biotic stimulus
response to interleukin−1
sister chromatid segregation
T cell mediated immunity
1
0.8
0.6
0.4
0.2
0
REGULATION_OF_LEUKOCYTE_MEDIATED_CYTOTOXICITY
T_CELL_MEDIATED_CYTOTOXICITY
POSITIVE_REGULATION_OF_T_CELL_MEDIATED_CYTOTOXICITY
REGULATION_OF_T_CELL_MEDIATED_CYTOTOXICITY
POSITIVE_REGULATION_OF_PRODUCTION_OF_MOLECULAR_MEDIATOR_OF_IMMUNE_RESPONSE
REGULATION_OF_PRODUCTION_OF_MOLECULAR_MEDIATOR_OF_IMMUNE_RESPONSE
T_CELL_CYTOKINE_PRODUCTION
POSITIVE_REGULATION_OF_CYTOKINE_PRODUCTION_INVOLVED_IN_IMMUNE_RESPONSE
POSITIVE_REGULATION_OF_T_CELL_CYTOKINE_PRODUCTION
T_CELL_MEDIATED_IMMUNITY
LEUKOCYTE_MEDIATED_IMMUNITY
LYMPHOCYTE_MEDIATED_IMMUNITY
POSITIVE_REGULATION_OF_T_CELL_MEDIATED_IMMUNITY
REGULATION_OF_T_CELL_MEDIATED_IMMUNITY
NEGATIVE_REGULATION_OF_IMMUNE_EFFECTOR_PROCESS
NEGATIVE_REGULATION_OF_LEUKOCYTE_MEDIATED_IMMUNITY
NEGATIVE_REGULATION_OF_LYMPHOCYTE_MEDIATED_IMMUNITY
POSITIVE_REGULATION_OF_IMMUNE_EFFECTOR_PROCESS
POSITIVE_REGULATION_OF_LEUKOCYTE_MEDIATED_IMMUNITY
POSITIVE_REGULATION_OF_LYMPHOCYTE_MEDIATED_IMMUNITY
REGULATION_OF_IMMUNE_EFFECTOR_PROCESS
REGULATION_OF_LEUKOCYTE_MEDIATED_IMMUNITY
REGULATION_OF_LYMPHOCYTE_MEDIATED_IMMUNITY
POSITIVE_REGULATION_OF_ADAPTIVE_IMMUNE_RESPONSE
REGULATION_OF_ADAPTIVE_IMMUNE_RESPONSE
REGULATION_OF_T_HELPER_1_TYPE_IMMUNE_RESPONSE
ADAPTIVE_IMMUNE_RESPONSE
ADAPTIVE_IMMUNE_RESPONSE_BASED_ON_SOMATIC_RECOMBINATION_OF_IMMUNE_RECEPTORS_BUILT_FROM_IMMUNOGLOBULIN_SUPERFAMILY_DOMAINS
CYTOKINE_PRODUCTION_INVOLVED_IN_IMMUNE_RESPONSE
NEGATIVE_REGULATION_OF_MYELOID_CELL_DIFFERENTIATION
HUMORAL_IMMUNE_RESPONSE
IMMUNE_RESPONSE_TO_TUMOR_CELL
ANTIBACTERIAL_HUMORAL_RESPONSE
ANTIMICROBIAL_HUMORAL_RESPONSE
NEGATIVE_REGULATION_OF_NATURAL_KILLER_CELL_MEDIATED_IMMUNITY
REGULATION_OF_NATURAL_KILLER_CELL_MEDIATED_IMMUNITY
NEGATIVE_REGULATION_OF_IMMUNE_RESPONSE
NEGATIVE_REGULATION_OF_INNATE_IMMUNE_RESPONSE
ANTIGEN_PROCESSING_AND_PRESENTATION_OF_EXOGENOUS_ANTIGEN
ANTIGEN_PROCESSING_AND_PRESENTATION_OF_EXOGENOUS_PEPTIDE_ANTIGEN
ANTIGEN_PROCESSING_AND_PRESENTATION_OF_ENDOGENOUS_ANTIGEN
ANTIGEN_PROCESSING_AND_PRESENTATION_OF_ENDOGENOUS_PEPTIDE_ANTIGEN
ANTIGEN_PROCESSING_AND_PRESENTATION_OF_PEPTIDE_ANTIGEN
ANTIGEN_PROCESSING_AND_PRESENTATION_OF_PEPTIDE_ANTIGEN_VIA_MHC_CLASS_I
POSITIVE_REGULATION_OF_ALPHA_BETA_T_CELL_ACTIVATION
POSITIVE_REGULATION_OF_CD4_POSITIVE_ALPHA_BETA_T_CELL_ACTIVATION
POSITIVE_REGULATION_OF_ALPHA_BETA_T_CELL_PROLIFERATION
POSITIVE_REGULATION_OF_T_CELL_PROLIFERATION
REGULATION_OF_T_HELPER_CELL_DIFFERENTIATION
REGULATION_OF_T_CELL_PROLIFERATION
ALPHA_BETA_T_CELL_PROLIFERATION
T_CELL_PROLIFERATION
T_CELL_ACTIVATION
ALPHA_BETA_T_CELL_ACTIVATION
CD4_POSITIVE_ALPHA_BETA_T_CELL_ACTIVATION
REGULATION_OF_NATURAL_KILLER_CELL_ACTIVATION
REGULATION_OF_ALPHA_BETA_T_CELL_ACTIVATION
REGULATION_OF_CD4_POSITIVE_ALPHA_BETA_T_CELL_ACTIVATION
REGULATION_OF_LYMPHOCYTE_ACTIVATION
REGULATION_OF_T_CELL_ACTIVATION
LIPOPOLYSACCHARIDE_MEDIATED_SIGNALING_PATHWAY
DEFENSE_RESPONSE_TO_BACTERIUM
DEFENSE_RESPONSE_TO_GRAM_POSITIVE_BACTERIUM
RESPONSE_TO_TUMOR_CELL
DETECTION_OF_BIOTIC_STIMULUS
DETECTION_OF_EXTERNAL_BIOTIC_STIMULUS
DETECTION_OF_OTHER_ORGANISM
NEGATIVE_REGULATION_OF_RESPONSE_TO_BIOTIC_STIMULUS
REGULATION_OF_RESPONSE_TO_BIOTIC_STIMULUS
NEGATIVE_REGULATION_OF_DEFENSE_RESPONSE
POSITIVE_REGULATION_OF_ACUTE_INFLAMMATORY_RESPONSE
REGULATION_OF_ACUTE_INFLAMMATORY_RESPONSE
POSITIVE_REGULATION_OF_LEUKOCYTE_PROLIFERATION
REGULATION_OF_LEUKOCYTE_PROLIFERATION
POSITIVE_REGULATION_OF_LEUKOCYTE_CELL_CELL_ADHESION
POSITIVE_REGULATION_OF_CELL_ADHESION
POSITIVE_REGULATION_OF_CELL_CELL_ADHESION
LEUKOCYTE_CELL_CELL_ADHESION
REGULATION_OF_CELL_CELL_ADHESION
POSITIVE_REGULATION_OF_CHEMOKINE_PRODUCTION
POSITIVE_REGULATION_OF_TYPE_II_INTERFERON_PRODUCTION
CHEMOKINE_PRODUCTION
TYPE_II_INTERFERON_PRODUCTION
DOUBLE_STRAND_BREAK_REPAIR
RECOMBINATIONAL_REPAIR
NEGATIVE_REGULATION_OF_LIPID_CATABOLIC_PROCESS
CYTOPLASMIC_TRANSLATION
DNA_REPLICATION
DNA_REPLICATION_INITIATION
POSITIVE_REGULATION_OF_DNA_METABOLIC_PROCESS
POSITIVE_REGULATION_OF_HISTONE_H3_K4_METHYLATION
POSITIVE_REGULATION_OF_CELL_KILLING
NEGATIVE_REGULATION_OF_CELL_KILLING
REGULATION_OF_CELL_KILLING
INTERLEUKIN_1_MEDIATED_SIGNALING_PATHWAY
CELLULAR_RESPONSE_TO_INTERLEUKIN_1
RESPONSE_TO_INTERLEUKIN_1
CHROMOSOME_LOCALIZATION
PROTEIN_LOCALIZATION_TO_CHROMOSOME
EPITHELIAL_STRUCTURE_MAINTENANCE
MONONUCLEAR_CELL_DIFFERENTIATION
CELL_CYCLE_CHECKPOINT_SIGNALING
NEGATIVE_REGULATION_OF_CELL_CYCLE
NEGATIVE_REGULATION_OF_CELL_CYCLE_PHASE_TRANSITION
REGULATION_OF_CELL_CYCLE_PHASE_TRANSITION
REGULATION_OF_CHROMOSOME_SEGREGATION
REGULATION_OF_CHROMOSOME_SEPARATION
REGULATION_OF_CYTOKINESIS
MITOTIC_CYTOKINESIS
CYTOKINESIS
CYTOSKELETON_DEPENDENT_CYTOKINESIS
MEIOTIC_CELL_CYCLE
MEIOTIC_CELL_CYCLE_PROCESS
CHROMOSOME_SEPARATION
CHROMOSOME_SEGREGATION
NUCLEAR_CHROMOSOME_SEGREGATION
SISTER_CHROMATID_COHESION
MITOTIC_NUCLEAR_DIVISION
SPINDLE_ORGANIZATION
MICROTUBULE_CYTOSKELETON_ORGANIZATION_INVOLVED_IN_MITOSIS
MITOTIC_SPINDLE_ORGANIZATION
FEMALE_MEIOTIC_NUCLEAR_DIVISION
MEIOTIC_METAPHASE_CHROMOSOME_ALIGNMENT
NEGATIVE_REGULATION_OF_METAPHASE_ANAPHASE_TRANSITION_OF_CELL_CYCLE
METAPHASE_ANAPHASE_TRANSITION_OF_CELL_CYCLE
REGULATION_OF_MITOTIC_SISTER_CHROMATID_SEGREGATION
MITOTIC_METAPHASE_CHROMOSOME_ALIGNMENT
MITOTIC_SISTER_CHROMATID_SEGREGATION
SISTER_CHROMATID_SEGREGATION
NEGATIVE_REGULATION_OF_CHROMOSOME_ORGANIZATION
REGULATION_OF_CHROMOSOME_ORGANIZATION
POSITIVE_REGULATION_OF_CHROMOSOME_ORGANIZATION
POSITIVE_REGULATION_OF_ORGANELLE_ORGANIZATION
NEGATIVE_REGULATION_OF_NUCLEAR_DIVISION
ORGANELLE_FISSION
MICROTUBULE_ORGANIZING_CENTER_ORGANIZATION
CHROMOSOME_ORGANIZATION
TELOMERE_ORGANIZATION
CHROMOSOME_CONDENSATION
DNA_CONFORMATION_CHANGE
DNA_GEOMETRIC_CHANGE
POSITIVE_REGULATION_OF_CELLULAR_COMPONENT_BIOGENESIS
EPIGENETIC_REGULATION_OF_GENE_EXPRESSION
DNA_METHYLATION_DEPENDENT_HETEROCHROMATIN_FORMATION
NEGATIVE_REGULATION_OF_GENE_EXPRESSION_EPIGENETIC
DNA_REPLICATION_DEPENDENT_CHROMATIN_ASSEMBLY
REGULATION_OF_CHROMATIN_ORGANIZATION
HETEROCHROMATIN_ORGANIZATION
CHROMATIN_REMODELING
CHROMATIN_ORGANIZATION
PROTEIN_DNA_COMPLEX_ORGANIZATION
PROTEIN_DNA_COMPLEX_ASSEMBLY
NUCLEOSOME_ASSEMBLY
NUCLEOSOME_ORGANIZATION

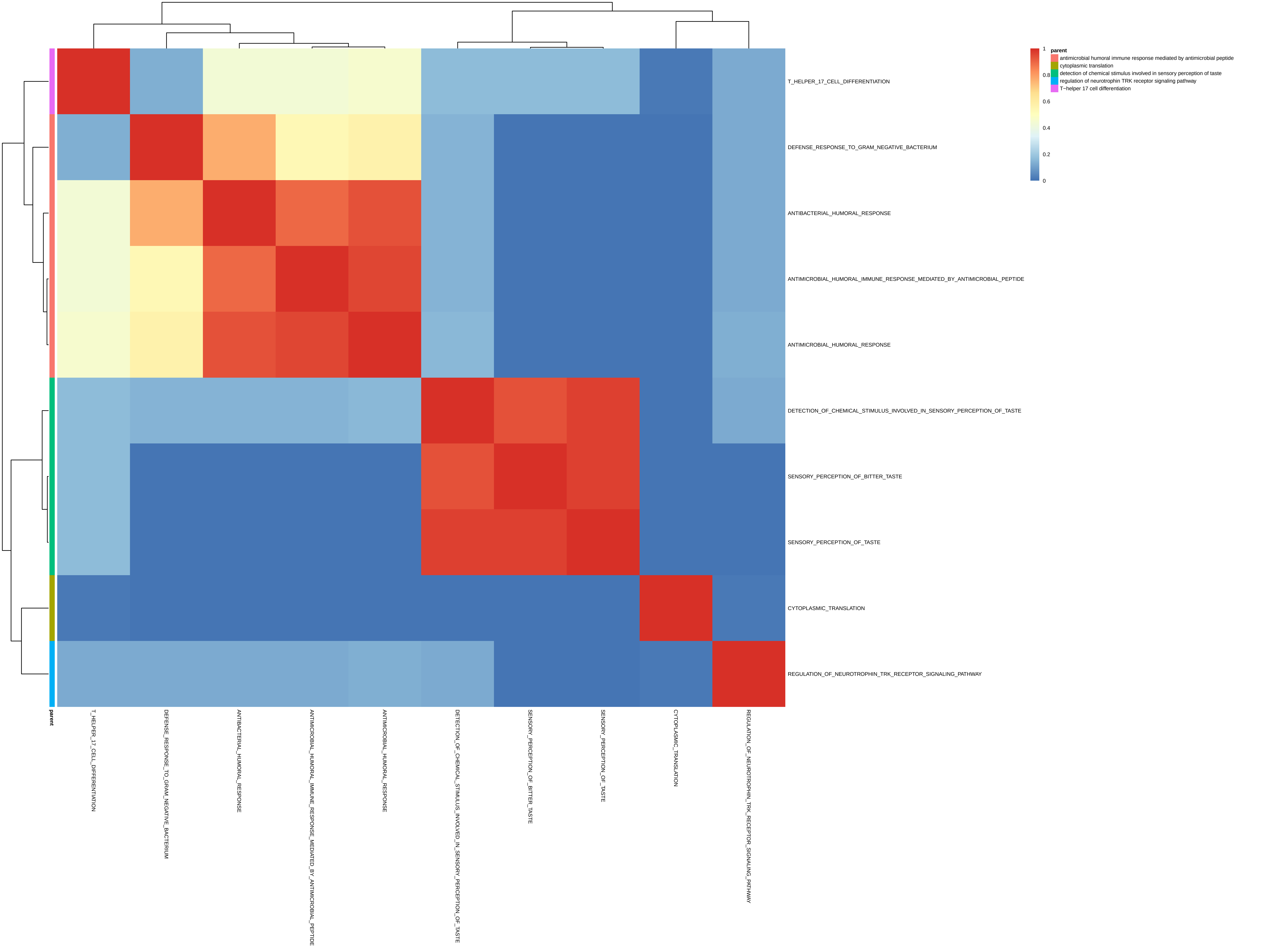

parent
antimicrobial humoral immune response mediated by antimicrobial peptide
cytoplasmic translation
detection of chemical stimulus involved in sensory perception of taste
regulation of neurotrophin TRK receptor signaling pathway
T−helper 17 cell differentiation
1
0.8
0.6
0.4
0.2
0
T_HELPER_17_CELL_DIFFERENTIATION
DEFENSE_RESPONSE_TO_GRAM_NEGATIVE_BACTERIUM
ANTIBACTERIAL_HUMORAL_RESPONSE
ANTIMICROBIAL_HUMORAL_IMMUNE_RESPONSE_MEDIATED_BY_ANTIMICROBIAL_PEPTIDE
ANTIMICROBIAL_HUMORAL_RESPONSE
DETECTION_OF_CHEMICAL_STIMULUS_INVOLVED_IN_SENSORY_PERCEPTION_OF_TASTE
SENSORY_PERCEPTION_OF_BITTER_TASTE
SENSORY_PERCEPTION_OF_TASTE
CYTOPLASMIC_TRANSLATION
REGULATION_OF_NEUROTROPHIN_TRK_RECEPTOR_SIGNALING_PATHWAY

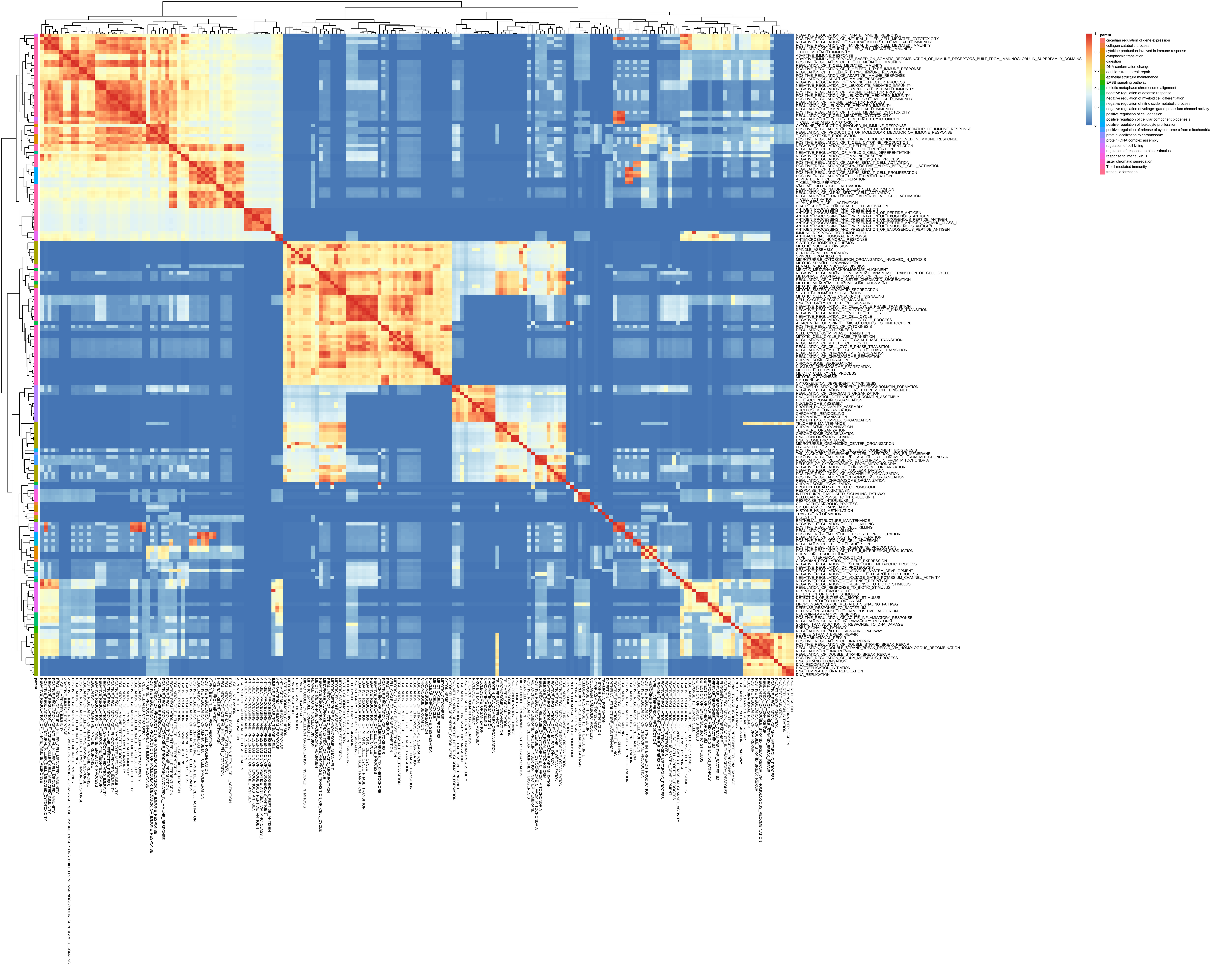
1
0.8
0.6
0.4
0.2
0
parent
circadian regulation of gene expression
collagen catabolic process
cytokine production involved in immune response
cytoplasmic translation
digestion
DNA conformation change
double−strand break repair
epithelial structure maintenance
ERBB signaling pathway
meiotic metaphase chromosome alignment
negative regulation of defense response
negative regulation of myeloid cell differentiation
negative regulation of nitric oxide metabolic process
negative regulation of voltage−gated potassium channel activity
positive regulation of cell adhesion
positive regulation of cellular component biogenesis
positive regulation of leukocyte proliferation
positive regulation of release of cytochrome c from mitochondria
protein localization to chromosome
protein−DNA complex assembly
regulation of cell killing
regulation of response to biotic stimulus
response to interleukin−1
sister chromatid segregation
T cell mediated immunity
trabecula formation
NEGATIVE_REGULATION_OF_INNATE_IMMUNE_RESPONSE
POSITIVE_REGULATION_OF_NATURAL_KILLER_CELL_MEDIATED_CYTOTOXICITY
NEGATIVE_REGULATION_OF_NATURAL_KILLER_CELL_MEDIATED_IMMUNITY
POSITIVE_REGULATION_OF_NATURAL_KILLER_CELL_MEDIATED_IMMUNITY
REGULATION_OF_NATURAL_KILLER_CELL_MEDIATED_IMMUNITY
T_CELL_MEDIATED_IMMUNITY
ADAPTIVE_IMMUNE_RESPONSE
ADAPTIVE_IMMUNE_RESPONSE_BASED_ON_SOMATIC_RECOMBINATION_OF_IMMUNE_RECEPTORS_BUILT_FROM_IMMUNOGLOBULIN_SUPERFAMILY_DOMAINS
POSITIVE_REGULATION_OF_T_CELL_MEDIATED_IMMUNITY
REGULATION_OF_T_CELL_MEDIATED_IMMUNITY
POSITIVE_REGULATION_OF_T_HELPER_1_TYPE_IMMUNE_RESPONSE
REGULATION_OF_T_HELPER_1_TYPE_IMMUNE_RESPONSE
POSITIVE_REGULATION_OF_ADAPTIVE_IMMUNE_RESPONSE
REGULATION_OF_ADAPTIVE_IMMUNE_RESPONSE
NEGATIVE_REGULATION_OF_IMMUNE_EFFECTOR_PROCESS
NEGATIVE_REGULATION_OF_LEUKOCYTE_MEDIATED_IMMUNITY
NEGATIVE_REGULATION_OF_LYMPHOCYTE_MEDIATED_IMMUNITY
POSITIVE_REGULATION_OF_IMMUNE_EFFECTOR_PROCESS
POSITIVE_REGULATION_OF_LEUKOCYTE_MEDIATED_IMMUNITY
POSITIVE_REGULATION_OF_LYMPHOCYTE_MEDIATED_IMMUNITY
REGULATION_OF_IMMUNE_EFFECTOR_PROCESS
REGULATION_OF_LEUKOCYTE_MEDIATED_IMMUNITY
REGULATION_OF_LYMPHOCYTE_MEDIATED_IMMUNITY
POSITIVE_REGULATION_OF_T_CELL_MEDIATED_CYTOTOXICITY
REGULATION_OF_T_CELL_MEDIATED_CYTOTOXICITY
POSITIVE_REGULATION_OF_LEUKOCYTE_MEDIATED_CYTOTOXICITY
T_CELL_MEDIATED_CYTOTOXICITY
CYTOKINE_PRODUCTION_INVOLVED_IN_IMMUNE_RESPONSE
POSITIVE_REGULATION_OF_PRODUCTION_OF_MOLECULAR_MEDIATOR_OF_IMMUNE_RESPONSE
REGULATION_OF_PRODUCTION_OF_MOLECULAR_MEDIATOR_OF_IMMUNE_RESPONSE
T_CELL_CYTOKINE_PRODUCTION
POSITIVE_REGULATION_OF_CYTOKINE_PRODUCTION_INVOLVED_IN_IMMUNE_RESPONSE
REGULATION_OF_T_CELL_CYTOKINE_PRODUCTION
NEGATIVE_REGULATION_OF_T_HELPER_CELL_DIFFERENTIATION
REGULATION_OF_T_HELPER_CELL_DIFFERENTIATION
NEGATIVE_REGULATION_OF_MYELOID_CELL_DIFFERENTIATION
NEGATIVE_REGULATION_OF_IMMUNE_RESPONSE
NEGATIVE_REGULATION_OF_IMMUNE_SYSTEM_PROCESS
POSITIVE_REGULATION_OF_ALPHA_BETA_T_CELL_ACTIVATION
POSITIVE_REGULATION_OF_CD4_POSITIVE_ALPHA_BETA_T_CELL_ACTIVATION
REGULATION_OF_T_CELL_PROLIFERATION
POSITIVE_REGULATION_OF_ALPHA_BETA_T_CELL_PROLIFERATION
POSITIVE_REGULATION_OF_T_CELL_PROLIFERATION
ALPHA_BETA_T_CELL_PROLIFERATION
T_CELL_PROLIFERATION
NATURAL_KILLER_CELL_ACTIVATION
REGULATION_OF_NATURAL_KILLER_CELL_ACTIVATION
REGULATION_OF_ALPHA_BETA_T_CELL_ACTIVATION
REGULATION_OF_CD4_POSITIVE_ALPHA_BETA_T_CELL_ACTIVATION
T_CELL_ACTIVATION
ALPHA_BETA_T_CELL_ACTIVATION
CD4_POSITIVE_ALPHA_BETA_T_CELL_ACTIVATION
ANTIGEN_PROCESSING_AND_PRESENTATION
ANTIGEN_PROCESSING_AND_PRESENTATION_OF_PEPTIDE_ANTIGEN
ANTIGEN_PROCESSING_AND_PRESENTATION_OF_EXOGENOUS_ANTIGEN
ANTIGEN_PROCESSING_AND_PRESENTATION_OF_EXOGENOUS_PEPTIDE_ANTIGEN
ANTIGEN_PROCESSING_AND_PRESENTATION_OF_PEPTIDE_ANTIGEN_VIA_MHC_CLASS_I
ANTIGEN_PROCESSING_AND_PRESENTATION_OF_ENDOGENOUS_ANTIGEN
ANTIGEN_PROCESSING_AND_PRESENTATION_OF_ENDOGENOUS_PEPTIDE_ANTIGEN
IMMUNE_RESPONSE_TO_TUMOR_CELL
ANTIBACTERIAL_HUMORAL_RESPONSE
ANTIMICROBIAL_HUMORAL_RESPONSE
SISTER_CHROMATID_COHESION
MITOTIC_NUCLEAR_DIVISION
SPINDLE_ASSEMBLY
CENTROSOME_DUPLICATION
SPINDLE_ORGANIZATION
MICROTUBULE_CYTOSKELETON_ORGANIZATION_INVOLVED_IN_MITOSIS
MITOTIC_SPINDLE_ORGANIZATION
FEMALE_MEIOTIC_NUCLEAR_DIVISION
MEIOTIC_METAPHASE_CHROMOSOME_ALIGNMENT
NEGATIVE_REGULATION_OF_METAPHASE_ANAPHASE_TRANSITION_OF_CELL_CYCLE
METAPHASE_ANAPHASE_TRANSITION_OF_CELL_CYCLE
REGULATION_OF_MITOTIC_SISTER_CHROMATID_SEGREGATION
MITOTIC_METAPHASE_CHROMOSOME_ALIGNMENT
MITOTIC_SPINDLE_ASSEMBLY
MITOTIC_SISTER_CHROMATID_SEGREGATION
SISTER_CHROMATID_SEGREGATION
MITOTIC_CELL_CYCLE_CHECKPOINT_SIGNALING
CELL_CYCLE_CHECKPOINT_SIGNALING
DNA_INTEGRITY_CHECKPOINT_SIGNALING
NEGATIVE_REGULATION_OF_CELL_CYCLE_PHASE_TRANSITION
NEGATIVE_REGULATION_OF_MITOTIC_CELL_CYCLE_PHASE_TRANSITION
NEGATIVE_REGULATION_OF_MITOTIC_CELL_CYCLE
NEGATIVE_REGULATION_OF_CELL_CYCLE
NEGATIVE_REGULATION_OF_CELL_CYCLE_PROCESS
ATTACHMENT_OF_SPINDLE_MICROTUBULES_TO_KINETOCHORE
POSITIVE_REGULATION_OF_CYTOKINESIS
REGULATION_OF_CYTOKINESIS
CELL_CYCLE_G2_M_PHASE_TRANSITION
MITOTIC_CELL_CYCLE_PHASE_TRANSITION
REGULATION_OF_CELL_CYCLE_G2_M_PHASE_TRANSITION
REGULATION_OF_MITOTIC_CELL_CYCLE
REGULATION_OF_CELL_CYCLE_PHASE_TRANSITION
REGULATION_OF_MITOTIC_CELL_CYCLE_PHASE_TRANSITION
REGULATION_OF_CHROMOSOME_SEGREGATION
REGULATION_OF_CHROMOSOME_SEPARATION
CHROMOSOME_SEPARATION
CHROMOSOME_SEGREGATION
NUCLEAR_CHROMOSOME_SEGREGATION
MEIOTIC_CELL_CYCLE
MEIOTIC_CELL_CYCLE_PROCESS
MITOTIC_CYTOKINESIS
CYTOKINESIS
CYTOSKELETON_DEPENDENT_CYTOKINESIS
DNA_METHYLATION_DEPENDENT_HETEROCHROMATIN_FORMATION
NEGATIVE_REGULATION_OF_GENE_EXPRESSION_EPIGENETIC
REGULATION_OF_CHROMATIN_ORGANIZATION
DNA_REPLICATION_DEPENDENT_CHROMATIN_ASSEMBLY
HETEROCHROMATIN_ORGANIZATION
NUCLEOSOME_ASSEMBLY
PROTEIN_DNA_COMPLEX_ASSEMBLY
NUCLEOSOME_ORGANIZATION
CHROMATIN_REMODELING
CHROMATIN_ORGANIZATION
PROTEIN_DNA_COMPLEX_ORGANIZATION
TELOMERE_MAINTENANCE
CHROMOSOME_ORGANIZATION
TELOMERE_ORGANIZATION
CHROMOSOME_CONDENSATION
DNA_CONFORMATION_CHANGE
DNA_GEOMETRIC_CHANGE
MICROTUBULE_ORGANIZING_CENTER_ORGANIZATION
ORGANELLE_FISSION
POSITIVE_REGULATION_OF_CELLULAR_COMPONENT_BIOGENESIS
TAIL_ANCHORED_MEMBRANE_PROTEIN_INSERTION_INTO_ER_MEMBRANE
POSITIVE_REGULATION_OF_RELEASE_OF_CYTOCHROME_C_FROM_MITOCHONDRIA
REGULATION_OF_RELEASE_OF_CYTOCHROME_C_FROM_MITOCHONDRIA
RELEASE_OF_CYTOCHROME_C_FROM_MITOCHONDRIA
NEGATIVE_REGULATION_OF_CHROMOSOME_ORGANIZATION
NEGATIVE_REGULATION_OF_NUCLEAR_DIVISION
POSITIVE_REGULATION_OF_ORGANELLE_ORGANIZATION
POSITIVE_REGULATION_OF_CHROMOSOME_ORGANIZATION
REGULATION_OF_CHROMOSOME_ORGANIZATION
CHROMOSOME_LOCALIZATION
PROTEIN_LOCALIZATION_TO_CHROMOSOME
RESPONSE_TO_ANGIOTENSIN
INTERLEUKIN_1_MEDIATED_SIGNALING_PATHWAY
CELLULAR_RESPONSE_TO_INTERLEUKIN_1
RESPONSE_TO_INTERLEUKIN_1
COLLAGEN_CATABOLIC_PROCESS
CYTOPLASMIC_TRANSLATION
HISTONE_H3_K4_METHYLATION
TRABECULA_FORMATION
DIGESTION
EPITHELIAL_STRUCTURE_MAINTENANCE
NEGATIVE_REGULATION_OF_CELL_KILLING
POSITIVE_REGULATION_OF_CELL_KILLING
REGULATION_OF_CELL_KILLING
POSITIVE_REGULATION_OF_LEUKOCYTE_PROLIFERATION
REGULATION_OF_LEUKOCYTE_PROLIFERATION
POSITIVE_REGULATION_OF_CELL_ADHESION
REGULATION_OF_CELL_CELL_ADHESION
POSITIVE_REGULATION_OF_CHEMOKINE_PRODUCTION
POSITIVE_REGULATION_OF_TYPE_II_INTERFERON_PRODUCTION
CHEMOKINE_PRODUCTION
TYPE_II_INTERFERON_PRODUCTION
CIRCADIAN_REGULATION_OF_GENE_EXPRESSION
NEGATIVE_REGULATION_OF_NITRIC_OXIDE_METABOLIC_PROCESS
NEGATIVE_REGULATION_OF_PROTEOLYSIS
NEGATIVE_REGULATION_OF_NERVOUS_SYSTEM_DEVELOPMENT
NEGATIVE_REGULATION_OF_MUSCLE_CELL_APOPTOTIC_PROCESS
NEGATIVE_REGULATION_OF_VOLTAGE_GATED_POTASSIUM_CHANNEL_ACTIVITY
NEGATIVE_REGULATION_OF_DEFENSE_RESPONSE
NEGATIVE_REGULATION_OF_RESPONSE_TO_BIOTIC_STIMULUS
REGULATION_OF_RESPONSE_TO_BIOTIC_STIMULUS
RESPONSE_TO_TUMOR_CELL
DETECTION_OF_BIOTIC_STIMULUS
DETECTION_OF_EXTERNAL_BIOTIC_STIMULUS
DETECTION_OF_OTHER_ORGANISM
LIPOPOLYSACCHARIDE_MEDIATED_SIGNALING_PATHWAY
DEFENSE_RESPONSE_TO_BACTERIUM
DEFENSE_RESPONSE_TO_GRAM_POSITIVE_BACTERIUM
NEUROINFLAMMATORY_RESPONSE
POSITIVE_REGULATION_OF_ACUTE_INFLAMMATORY_RESPONSE
REGULATION_OF_ACUTE_INFLAMMATORY_RESPONSE
SIGNAL_TRANSDUCTION_IN_RESPONSE_TO_DNA_DAMAGE
ERBB_SIGNALING_PATHWAY
REGULATION_OF_NOTCH_SIGNALING_PATHWAY
DOUBLE_STRAND_BREAK_REPAIR
RECOMBINATIONAL_REPAIR
POSITIVE_REGULATION_OF_DNA_REPAIR
POSITIVE_REGULATION_OF_DOUBLE_STRAND_BREAK_REPAIR
REGULATION_OF_DOUBLE_STRAND_BREAK_REPAIR_VIA_HOMOLOGOUS_RECOMBINATION
REGULATION_OF_DNA_REPAIR
REGULATION_OF_DOUBLE_STRAND_BREAK_REPAIR
POSITIVE_REGULATION_OF_DNA_METABOLIC_PROCESS
DNA_STRAND_ELONGATION
DNA_RECOMBINATION
DNA_REPLICATION_INITIATION
DNA_TEMPLATED_DNA_REPLICATION
DNA_REPLICATION

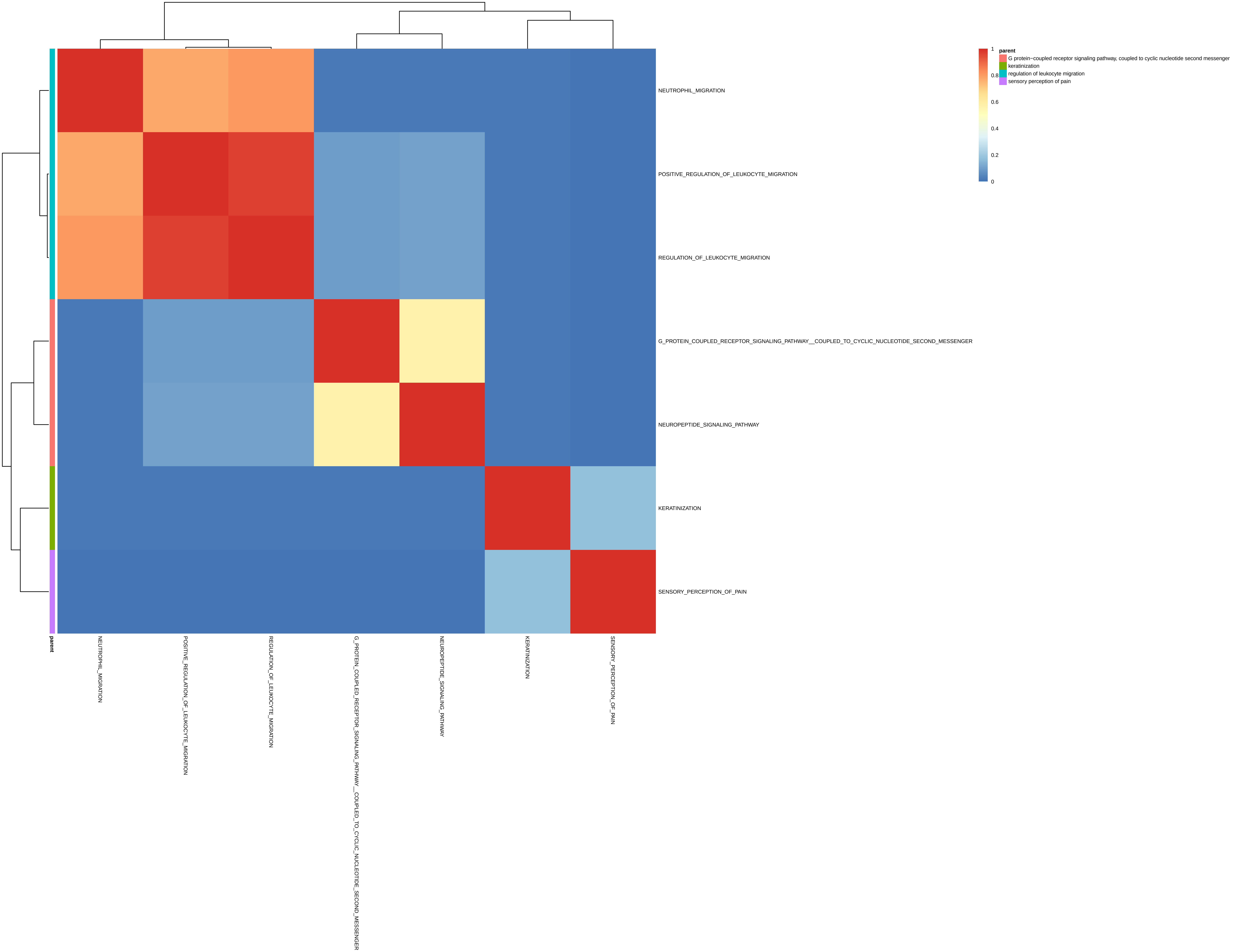
NEUTROPHIL_MIGRATION
POSITIVE_REGULATION_OF_LEUKOCYTE_MIGRATION
REGULATION_OF_LEUKOCYTE_MIGRATION
G_PROTEIN_COUPLED_RECEPTOR_SIGNALING_PATHWAY__COUPLED_TO_CYCLIC_NUCLEOTIDE_SECOND_MESSENGER
NEUROPEPTIDE_SIGNALING_PATHWAY
KERATINIZATION
SENSORY_PERCEPTION_OF_PAIN
parent
1
0.8
0.6
0.4
0.2
0
parent
G protein−coupled receptor signaling pathway, coupled to cyclic nucleotide second messenger
keratinization
regulation of leukocyte migration
sensory perception of pain

Suppl. Fig. S8. Treemap plots for the transformed RPE-PPM1D-T2-SA clones. Treemap plots generated by the rrvgo package express parent GO terms aggregating related GO:BP (gene ontology: biological functions) terms enriched significantly from the GSEA analysis (padj<0.05; Suppl Table 3). Clone #6 is not shown as it showed no significant GO:BP enrichment.

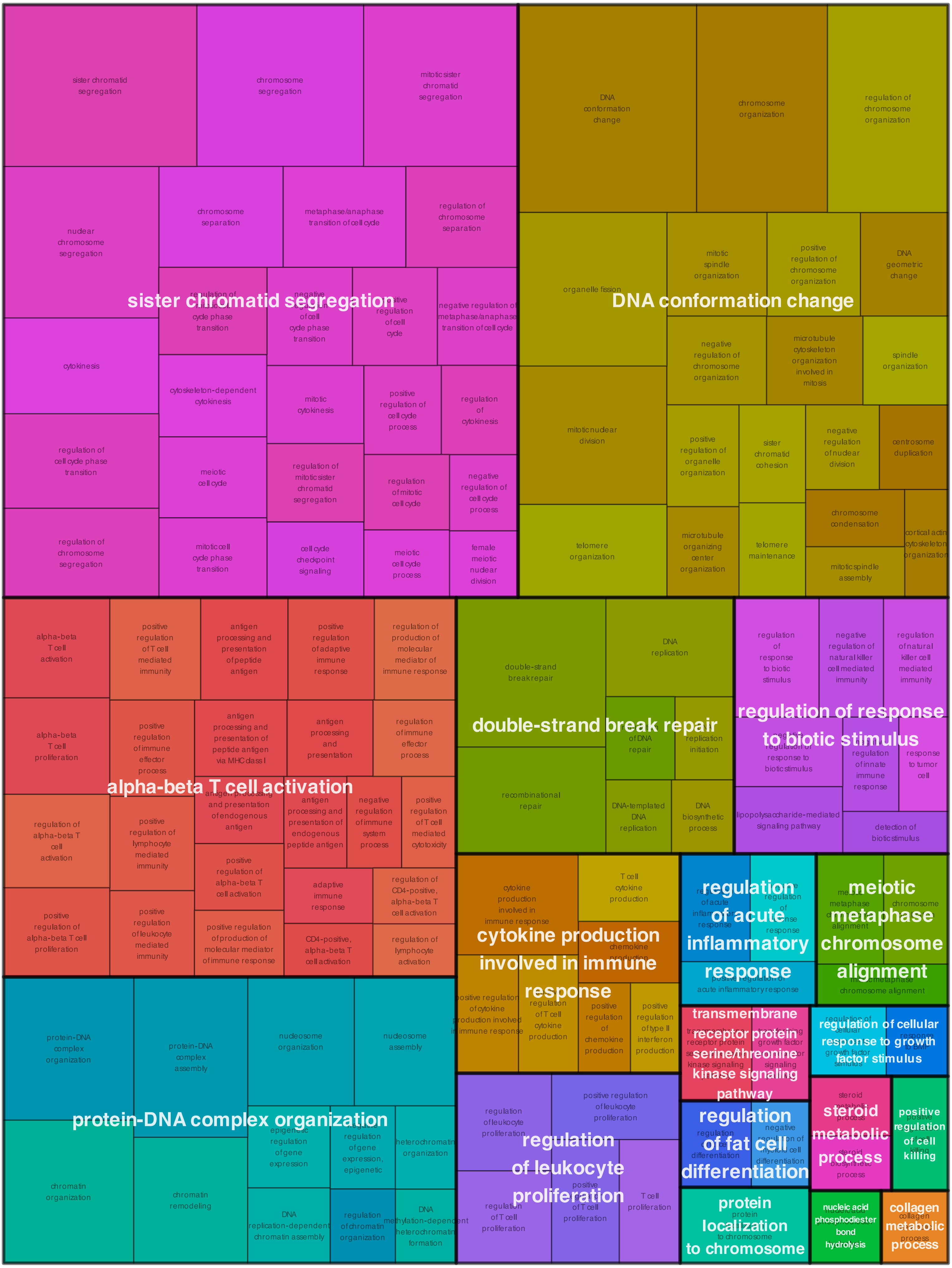
sister chromatid segregation
chromosome segregation
mitotic sister chromatid segregation
nuclear chromosome segregation
chromosome separation
metaphase/anaphase transition of cell cycle
regulation of chromosome separation
negative regulation of metaphase/anaphase transition of cell cycle
cytokinesis
cytoskeleton-dependent cytokinesis
mitotic cytokinesis
positive regulation of cell cycle process
regulation of cytokinesis
regulation of cell cycle phase transition
meiotic cell cycle
regulation of mitotic sister chromatid segregation
regulation of mitotic cell cycle
negative regulation of cell cycle process
regulation of chromosome segregation
mitotic cell cycle phase transition
cell cycle checkpoint signaling
meiotic cell cycle process
female meiotic nuclear division
DNA conformation change
chromosome organization
regulation of chromosome organization
organelle fission
mitotic spindle organization
positive regulation of chromosome organization
DNA geometric change
negative regulation of chromosome organization
microtubule cytoskeleton organization involved in mitosis
spindle organization
mitotic nuclear division
positive regulation of organelle organization
sister chromatid cohesion
negative regulation of nuclear division
centrosome duplication
chromosome condensation
cortical actin cytoskeleton organization
telomere organization
microtubule organizing center organization
telomere maintenance
mitotic spindle assembly
alpha-beta T cell activation
positive regulation of T cell mediated immunity
antigen processing and presentation of peptide antigen
positive regulation of adaptive immune response
regulation of production of molecular mediator of immune response
alpha-beta T cell proliferation
positive regulation of immune effector process
antigen processing and presentation of peptide antigen via MHC class I
antigen processing and presentation
regulation of immune effector process
regulation of alpha-beta T cell activation
positive regulation of lymphocyte mediated immunity
antigen processing and presentation of endogenous antigen
antigen processing and presentation of endogenous peptide antigen
negative regulation of immune system process
positive regulation of T cell mediated cytotoxicity
positive regulation of alpha-beta T cell activation
adaptive immune response
regulation of CD4-positive, alpha-beta T cell activation
positive regulation of alpha-beta T cell proliferation
positive regulation of leukocyte mediated immunity
positive regulation of production of molecular mediator of immune response
CD4-positive, alpha-beta T cell activation
regulation of lymphocyte activation
double-strand break repair
DNA replication
double-strand break repair
replication initiation
recombinational repair
DNA-templated DNA replication
DNA biosynthetic process
regulation of response to biotic stimulus
regulation of response to biotic stimulus
negative regulation of natural killer cell mediated immunity
regulation of natural killer cell mediated immunity
response to tumor cell
lipopolysaccharide-mediated signaling pathway
detection of biotic stimulus
cytokine production involved in immune response
T cell cytokine production
positive regulation of cytokine production involved in immune response
regulation of T cell cytokine production
positive regulation of chemokine production
positive regulation of type II interferon production
regulation of acute inflammatory response
acute inflammatory response
meiotic metaphase chromosome alignment
protein-DNA complex organization
protein-DNA complex assembly
nucleosome organization
nucleosome assembly
heterochromatin organization
chromatin organization
chromatin remodeling
DNA replication-dependent chromatin assembly
regulation of chromatin organization
DNA methylation-dependent heterochromatin formation
transmembrane receptor protein serine/threonine kinase signaling pathway
regulation of cellular response to growth factor stimulus
regulation of leukocyte proliferation
positive regulation of leukocyte proliferation
regulation of T cell proliferation
positive regulation of T cell proliferation
T cell proliferation
regulation of fat cell differentiation
steroid metabolic process
positive regulation of cell killing
protein localization to chromosome
nucleic acid phosphodiester bond hydrolysis
collagen metabolic process

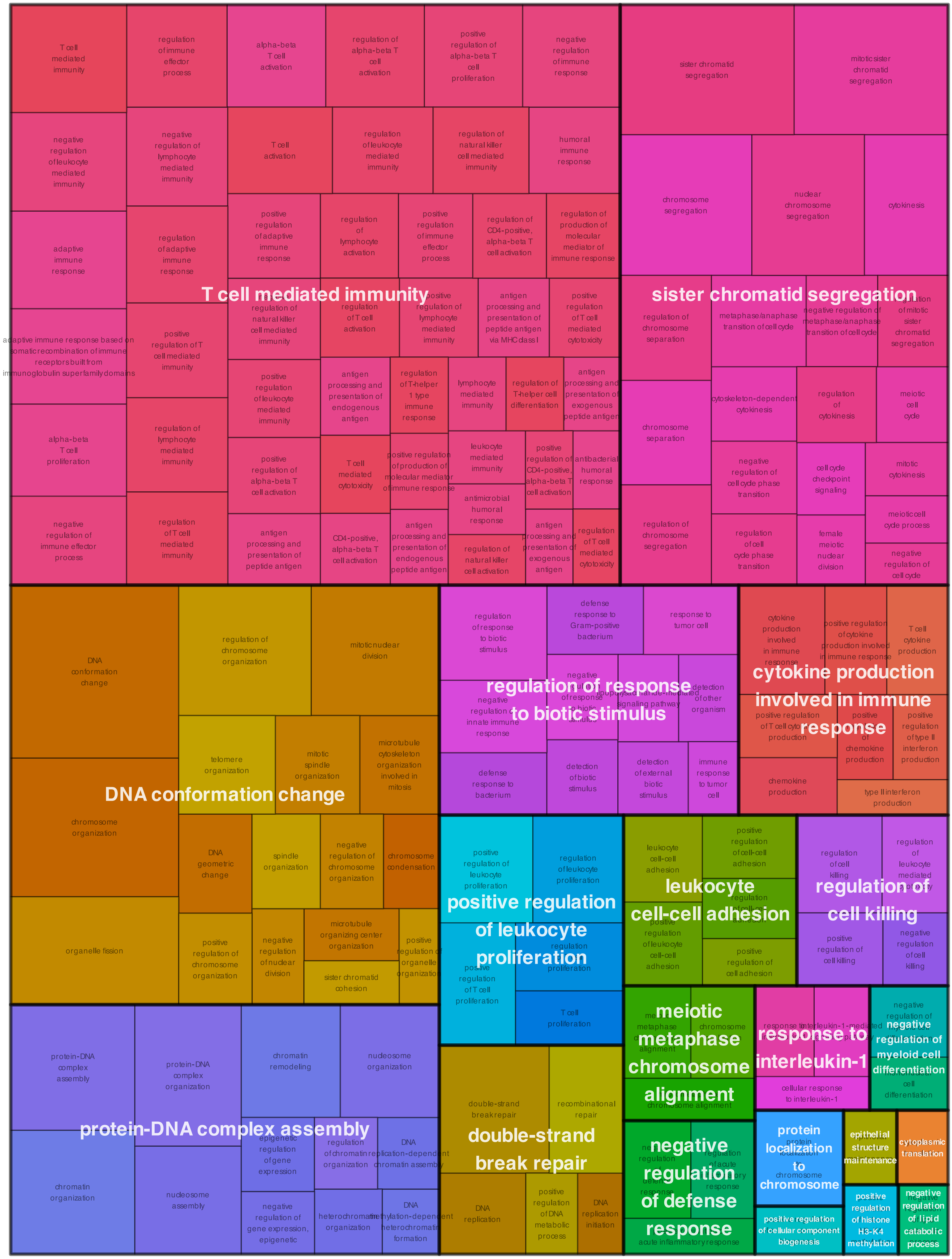

T cell mediated immunity
T cell mediated immunity
regulation of immune effector process
alpha-beta T cell activation
regulation of alpha-beta T cell activation
positive regulation of alpha-beta T cell proliferation
negative regulation of immune response
negative regulation of leukocyte mediated immunity
negative regulation of lymphocyte mediated immunity
T cell activation
regulation of leukocyte mediated immunity
regulation of natural killer cell mediated immunity
humoral immune response
adaptive immune response
regulation of adaptive immune response
positive regulation of adaptive immune response
regulation of lymphocyte activation
positive regulation of immune effector process
regulation of CD4-positive, alpha-beta T cell activation
regulation of production of molecular mediator of immune response
adaptive immune response based on somatic recombination of immune receptors built from immunoglobulin superfamily domains
positive regulation of T cell mediated immunity
regulation of natural killer cell mediated immunity
regulation of T cell activation
positive regulation of lymphocyte mediated immunity
antigen processing and presentation of peptide antigen via MHC class I
positive regulation of T cell mediated cytotoxicity
positive regulation of leukocyte mediated immunity
antigen processing and presentation of endogenous antigen
regulation of T-helper 1 type immune response
lymphocyte mediated immunity
regulation of T-helper cell differentiation
antigen processing and presentation of exogenous peptide antigen
alpha-beta T cell proliferation
regulation of lymphocyte mediated immunity
positive regulation of alpha-beta T cell activation
T cell mediated cytotoxicity
positive regulation of production of molecular mediator of immune response
leukocyte mediated immunity
positive regulation of CD4-positive, alpha-beta T cell activation
antibacterial humoral response
antimicrobial humoral response
negative regulation of immune effector process
regulation of T cell mediated immunity
antigen processing and presentation of peptide antigen
CD4-positive, alpha-beta T cell activation
antigen processing and presentation of endogenous peptide antigen
regulation of natural killer cell activation
antigen processing and presentation of exogenous antigen
regulation of T cell mediated cytotoxicity
sister chromatid segregation
sister chromatid segregation
mitotic sister chromatid segregation
chromosome segregation
nuclear chromosome segregation
cytokinesis
regulation of chromosome separation
metaphase/anaphase transition of cell cycle
negative regulation of metaphase/anaphase transition of cell cycle
regulation of mitotic sister chromatid segregation
cytoskeleton-dependent cytokinesis
regulation of cytokinesis
meiotic cell cycle
chromosome separation
negative regulation of cell cycle phase transition
cell cycle checkpoint signaling
mitotic cytokinesis
meiotic cell cycle process
regulation of chromosome segregation
regulation of cell cycle phase transition
female meiotic nuclear division
negative regulation of cell cycle
DNA conformation change
DNA conformation change
regulation of chromosome organization
mitotic nuclear division
telomere organization
mitotic spindle organization
microtubule cytoskeleton organization involved in mitosis
chromosome organization
DNA geometric change
spindle organization
negative regulation of chromosome organization
chromosome condensation
organelle fission
positive regulation of chromosome organization
negative regulation of nuclear division
microtubule organizing center organization
positive regulation of organelle organization
sister chromatid cohesion
regulation of response to biotic stimulus
regulation of response to biotic stimulus
defense response to Gram-positive bacterium
response to tumor cell
negative regulation of innate immune response
detection of other organism
defense response to bacterium
detection of biotic stimulus
detection of external biotic stimulus
immune response to tumor cell
cytokine production involved in immune response
cytokine production involved in immune response
positive regulation of cytokine production involved in immune response
T cell cytokine production
positive regulation of T cell cytokine production
positive regulation of chemokine production
positive regulation of type II interferon production
chemokine production
type II interferon production
positive regulation of leukocyte proliferation
positive regulation of leukocyte proliferation
regulation of leukocyte proliferation
positive regulation of T cell proliferation
T cell proliferation
leukocyte cell-cell adhesion
leukocyte cell-cell adhesion
positive regulation of cell-cell adhesion
positive regulation of leukocyte cell-cell adhesion
positive regulation of cell adhesion
regulation of cell killing
regulation of cell killing
regulation of leukocyte mediated cytotoxicity
positive regulation of cell killing
negative regulation of cell killing
protein-DNA complex assembly
protein-DNA complex assembly
protein-DNA complex organization
chromatin remodeling
nucleosome organization
epigenetic regulation of gene expression
regulation of chromatin organization
DNA replication-dependent chromatin assembly
chromatin organization
nucleosome assembly
negative regulation of gene expression, epigenetic
heterochromatin organization
DNA replication-dependent heterochromatin formation
double-strand break repair
double-strand break repair
recombinational repair
DNA replication
positive regulation of DNA metabolic process
DNA replication initiation
meiotic metaphase chromosome alignment
meiotic metaphase chromosome alignment
chromosome alignment
negative regulation of defense response
negative regulation of defense response
acute inflammatory response
response to interleukin-1
response to interleukin-1
cellular response to interleukin-1
negative regulation of myeloid cell differentiation
protein localization to chromosome
protein localization to chromosome
epithelial structure maintenance
cytoplasmic translation
positive regulation of cellular component biogenesis
positive regulation of histone H3-K4 methylation
negative regulation of lipid catabolic process

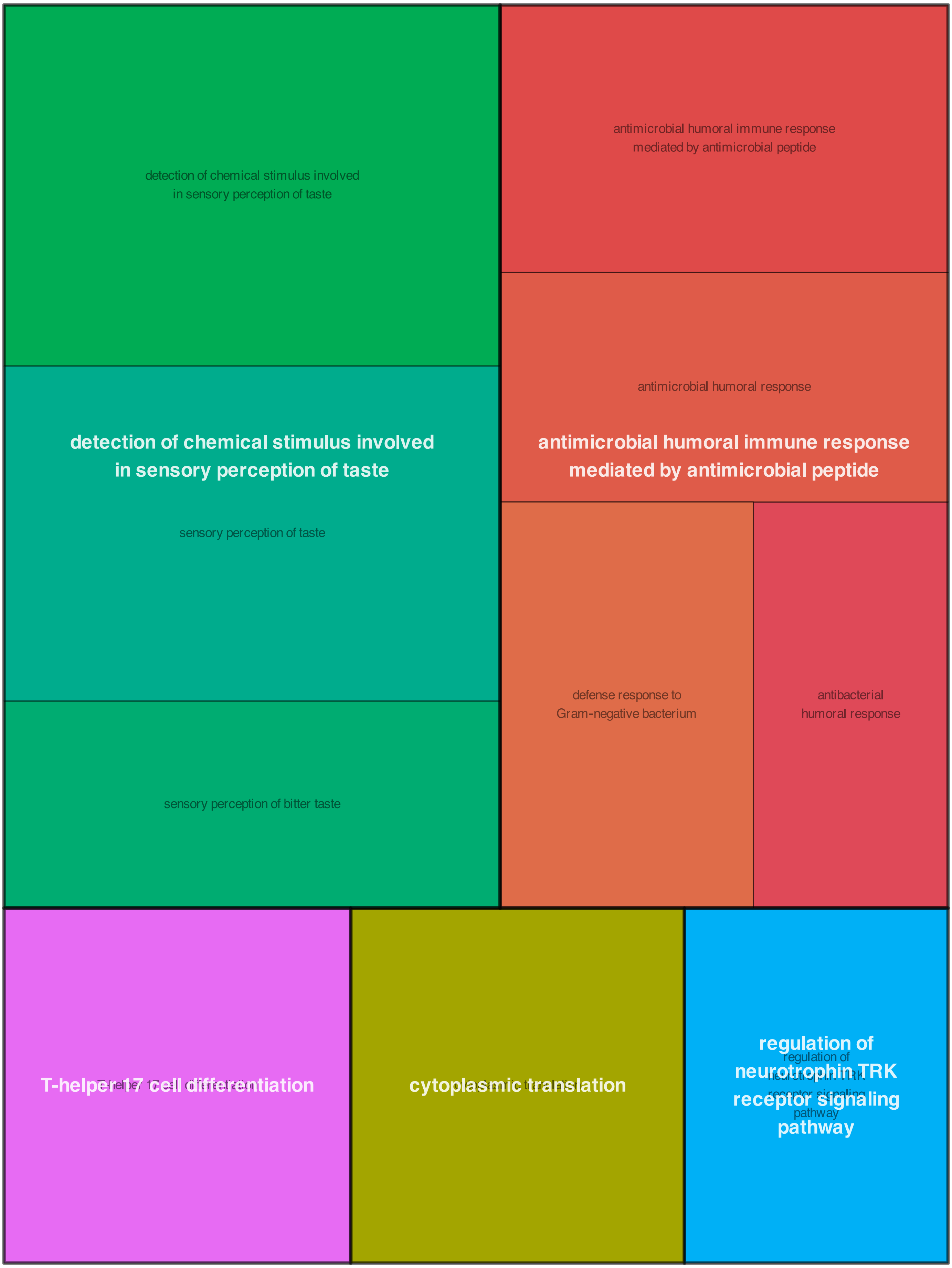
detection of chemical stimulus involved in sensory perception of taste
antimicrobial humoral immune response mediated by antimicrobial peptide
antimicrobial humoral response
detection of chemical stimulus involved in sensory perception of taste
antimicrobial humoral immune response mediated by antimicrobial peptide
sensory perception of taste
defense response to Gram-negative bacterium
antibacterial humoral response
sensory perception of bitter taste
regulation of neurotrophin TRK receptor signaling pathway
T-helper 17 cell differentiation
cytoplasmic translation

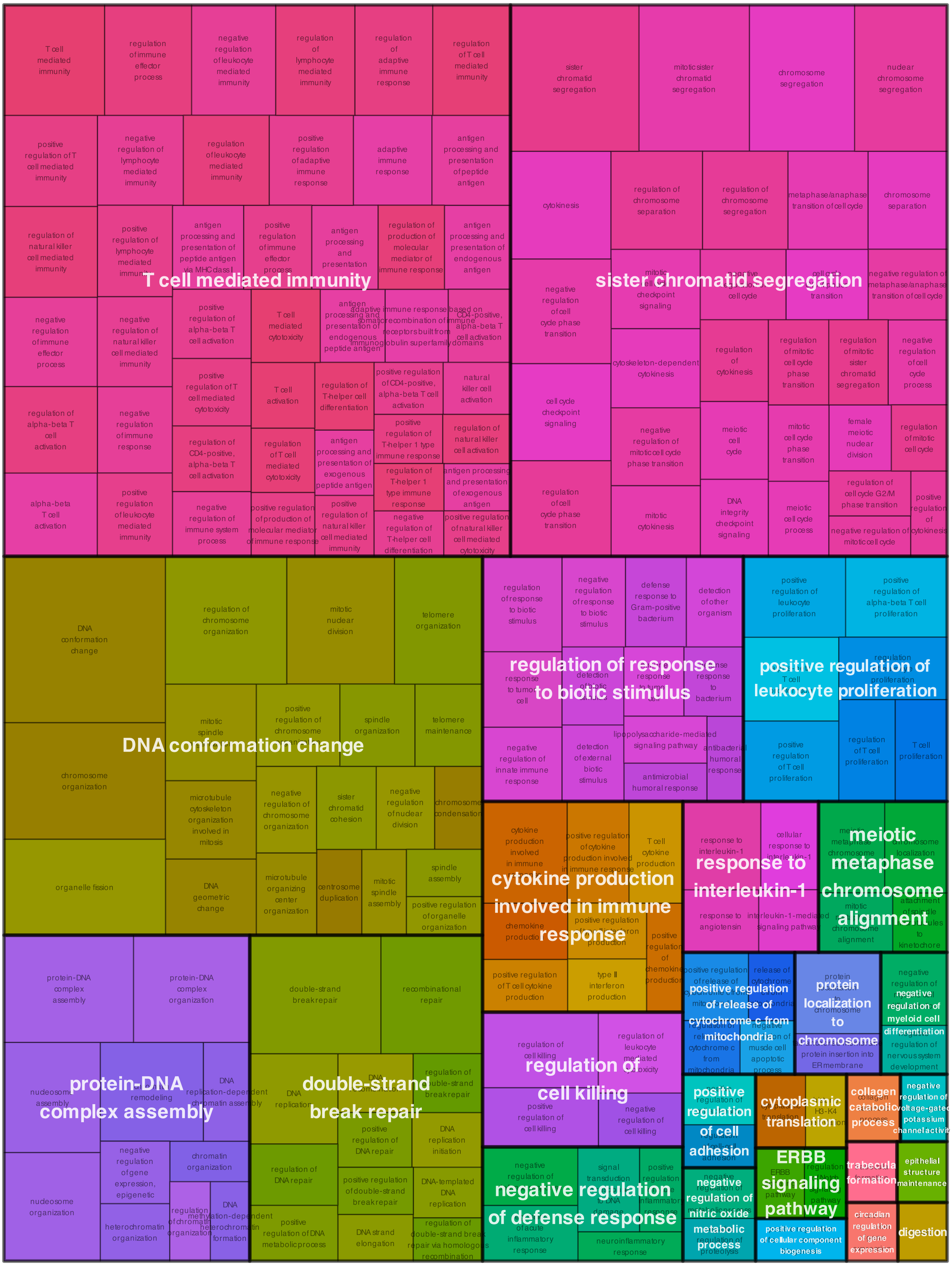
T cell mediated immunity
sister chromatid segregation
DNA conformation change
regulation of response to biotic stimulus
positive regulation of leukocyte proliferation
cytokine production involved in immune response
response to interleukin-1
meiotic metaphase chromosome alignment
protein-DNA complex assembly
double-strand break repair
regulation of cell killing
positive regulation of release of cytochrome c from mitochondria
protein localization to chromosome
negative regulation of myeloid cell differentiation
positive regulation of cell adhesion
cytoplasmic translation
collagen catabolic process
negative regulation of voltage-gated potassium channel activity
negative regulation of defense response
negative regulation of nitric oxide metabolic process
ERBB signaling pathway
positive regulation of cellular component biogenesis
trabecula formation
epithelial structure maintenance
circadian regulation of gene expression
digestion

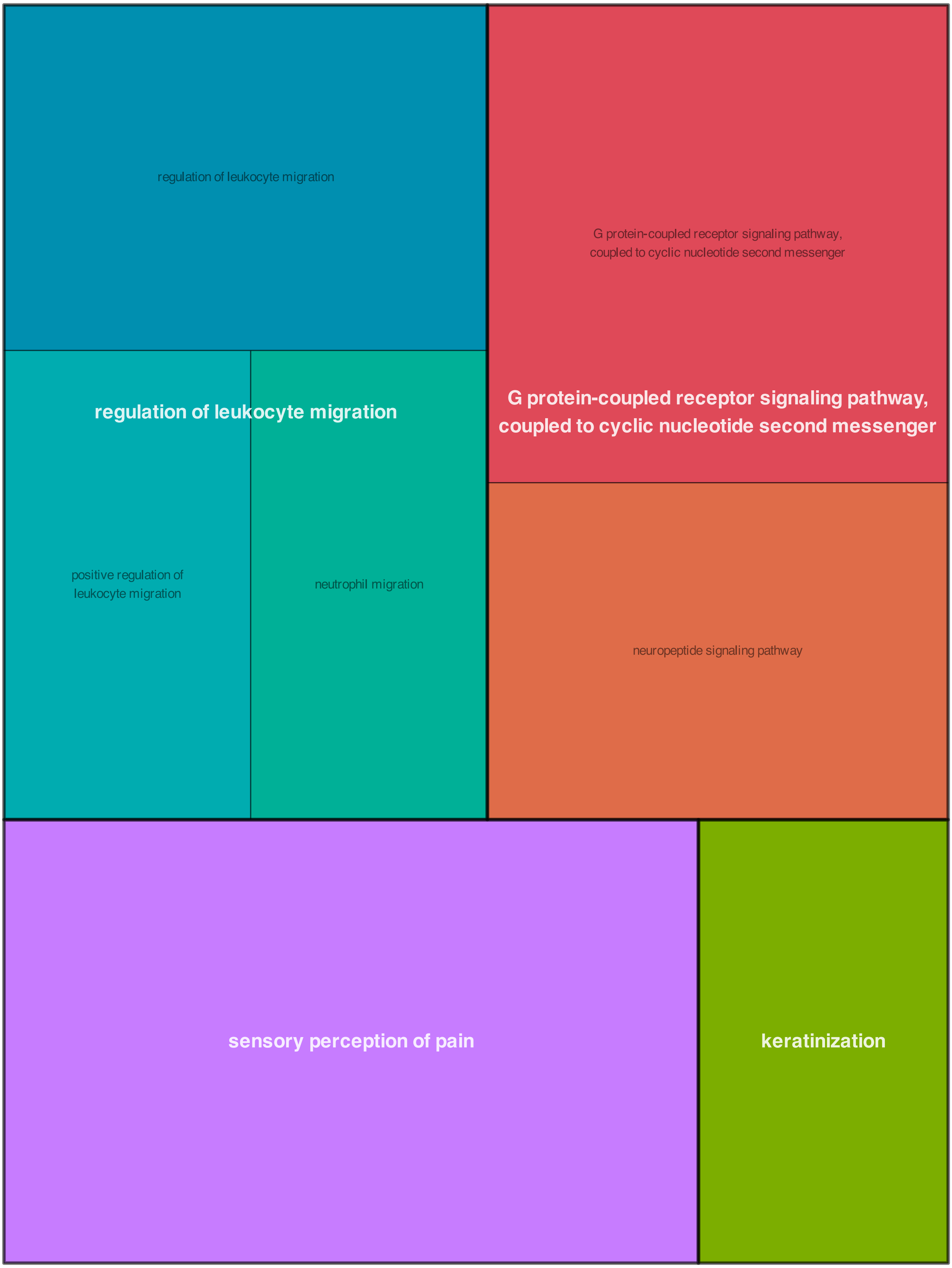
regulation of leukocyte migration
G protein-coupled receptor signaling pathway, coupled to cyclic nucleotide second messenger
regulation of leukocyte migration
G protein-coupled receptor signaling pathway, coupled to cyclic nucleotide second messenger
positive regulation of leukocyte migration
neutrophil migration
neuropeptide signaling pathway
sensory perception of pain
keratinization

Suppl. Fig. S9. Significantly enriched parental GO:BP terms in individual RPE-PPM1D-T2-SA clones. Dots represent parental terms from GO:BP enriched in individual clones significantly (p_{adj}<0.05; Suppl Table 3). The two largest collections of pathways including the DNA repair/replication processes and immune responses are highlighted in red and blue respectively.

Parent GO term	RPE-PPM1D-T2-SA Clone #1	Clone #2	Clone #4	Clone #3	Clone #5	Clone #6
Cytokine production involved in immune response	●	●	●			
Cytoplasmic translation		●	●	●		
DNA conformation change	●	●	●			
Double−strand break repair	●	●	●			
Meiotic metaphase chromosome alignment	●	●	●			
Protein localization to chromosome	●	●	●			
Regulation of response to biotic stimulus	●	●	●			
Sister chromatid segregation	●	●	●			
Epithelial structure maintenance		●	●			
Negative regulation of defense response		●	●			
Negative regulation of myeloid cell differentiation		●	●			
Positive regulation of leukocyte proliferation		●	●			
Protein localization to chromosome		●	●			
Protein−DNA complex assembly		●	●			
Regulation of cell killing		●	●			
Response to interleukin−1		●	●			
T cell mediated immunity		●	●			
Alpha−beta T cell activation	●					
Antimicrobial humoral immune response mediated by antimicrobial peptide				●		
Circadian regulation of gene expression			●			
G protein−coupled receptor signaling pathway					●	
Collagen catabolic process			●			
Collagen metabolic process	●					
Digestion			●			
Detection of chemical stimulus involved in sensory perception of taste				●		
ERBB signaling pathway			●			
Keratinization					●	
Leukocyte cell−cell adhesion		●				
Nucleic acid phosphodiester bond hydrolysis	●					
Negative regulation of lipid catabolic process		●				
Negative regulation of nitric oxide metabolic process			●			
Negative regulation of voltage−gated potassium channel activity			●			
Positive regulation of cell killing	●					
Positive regulation of cell adhesion			●			
Positive regulation of histone H3−K4 methylation		●				
Positive regulation of release of cytochrome c from mitochondria			●			
Protein−DNA complex organization	●					
Regulation of acute inflammatory response	●					
Regulation of cellular response to growth factor stimulus	●					
Regulation of fat cell differentiation	●					
Regulation of leukocyte migration					●	
Regulation of leukocyte proliferation	●					
Regulation of neurotrophin TRK receptor signaling pathway				●		
Sensory perception of pain					●	
Steroid metabolic process	●					
Transmembrane receptor protein serine/threonine kinase signaling pathway	●					
T−helper 17 cell differentiation				●		
Trabecula formation			●			

Suppl. Fig. S10. Truncated PPM1D promotes override of OIS in cells expressing active RAS

A) Parental BJ-hTert-HRASV12^{ER-TAM}, BJ-hTert-HRASV12^{ER-TAM}-PPM1D-T2, and BJ-hTert-HRASV12^{ER-TAM}-PPM1D-T2-60 cells that survived 2-months of HRASV12 expression were treated with combinations of 4OHT and PPM1D inhibitor for 20 days as indicated, fixed and stained for HP1, Histone H3K9me3 and DAPI and analyzed by ScanR microscopy. Representative images from three independent repeats are shown. Bar indicates 5 m. **B)** Quantification of the mean nuclear intensity of HP1 in BJ cells from A. **C)** Quantification of the mean nuclear intensity of Histone H3K9me3 in BJ cells from A. **D)** Quantification of the average nuclear size of BJ cells from A.

Supplementary Fig. S10

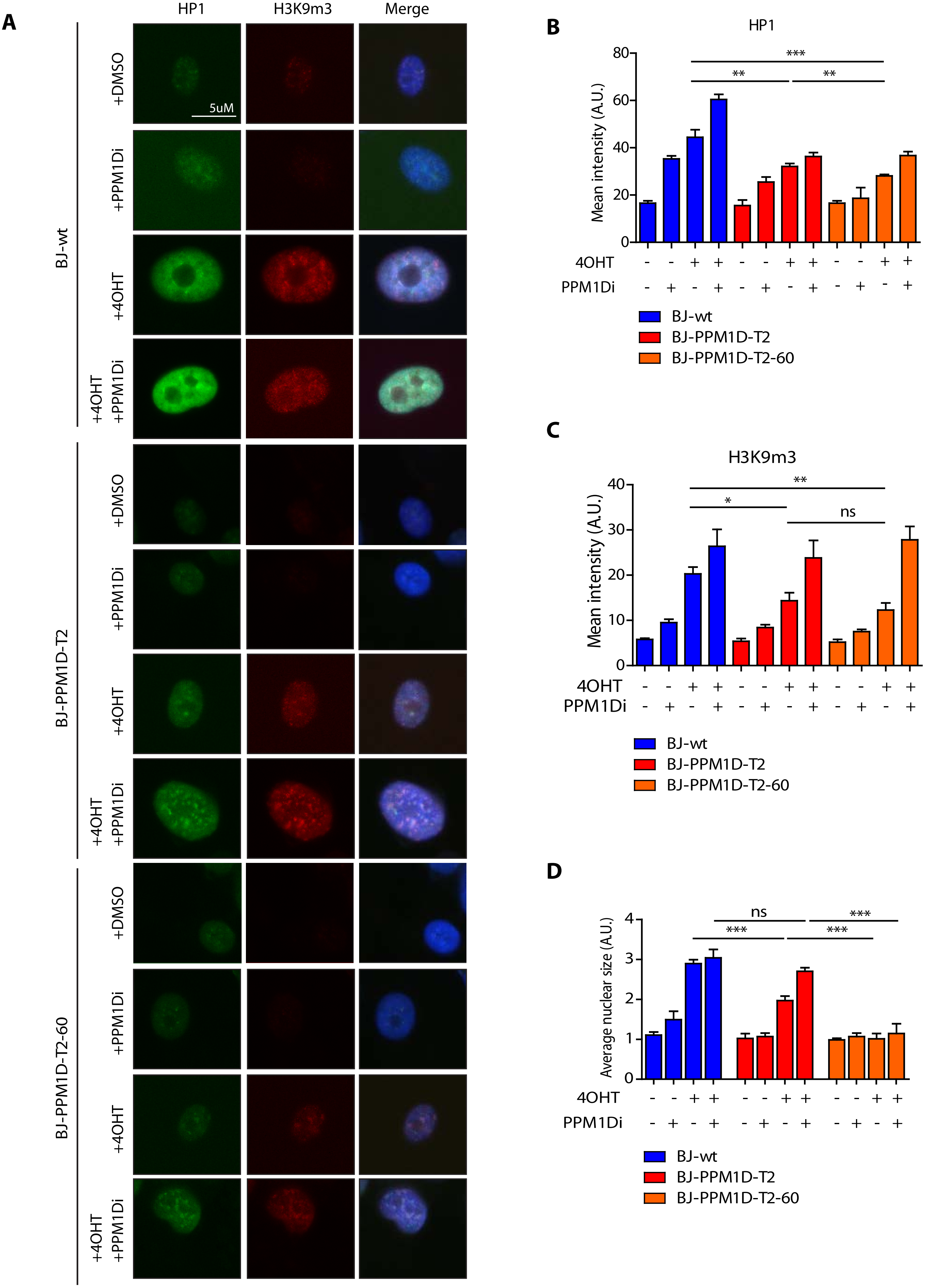

Suppl. Fig. S11. Senescence induced by RAS is p53 dependent

A) BJ-hTert-HRASV12$^{ER\text{-}TAM}$-TP53KO and BJ-hTert-HRASV12$^{ER\text{-}TAM}$-PPM1D-T1-TP53KO cells were cultivated in the presence of 4OHT and/or PPM1Di as indicated for 20 d. Cells were incubated with EdU 24 h prior fixation at indicated time intervals and EdU was labelled by CLICK-IT reaction. Fraction of EdU positive cells was analysed using FACS. The right panel shows immunoblotting analysis of p53 levels in BJ-hTert-HRASV12$^{ER\text{-}TAM}$, BJ-hTert-HRASV12$^{ER\text{-}TAM}$-TP53KO and BJ-hTert-HRASV12$^{ER\text{-}TAM}$-PPM1D-T1-TP53KO cells. **B)** BJ-hTert-HRASV12$^{ER\text{-}TAM}$, BJ-hTert-HRASV12$^{ER\text{-}TAM}$-PPM1D-T1 and -T2 cells were induced or not with 4OHT for 5 days. Cells were labelled by propidium iodide and DAPI and fraction of dead cells was determined by flow cytometry. **C)** mRNA was extracted from cells treated as in B and expression of *CDKN1A*, *NOXA* and *BAX* was determined by qPCR. Statistical significance was determined by t-test, error bars indicate SD, n=3. **D)** Fraction of cells incorporating EdU was determined after 60 days of 4OHT treatment of BJ-RAS-PPM1D-T1 and BJ-RAS-PPM1D-T2 cells by flow cytometry. **E)** Parental BJ-hTert-HRASV12$^{ER\text{-}TAM}$, BJ-hTert-HRASV12$^{ER\text{-}TAM}$-PPM1D-T2, BJ-hTert-HRASV12$^{ER\text{-}TAM}$-PPM1D-T1-60 and BJ-hTert-HRASV12$^{ER\text{-}TAM}$-PPM1D-T2-60 cells that managed to proliferate for 2 months of continuous 4OHT induction were collected 6 h after exposure to a high dose of IR (5 Gy). The ability to induce p21 expression was evaluated by immunoblotting.

Supplementary Fig. 11

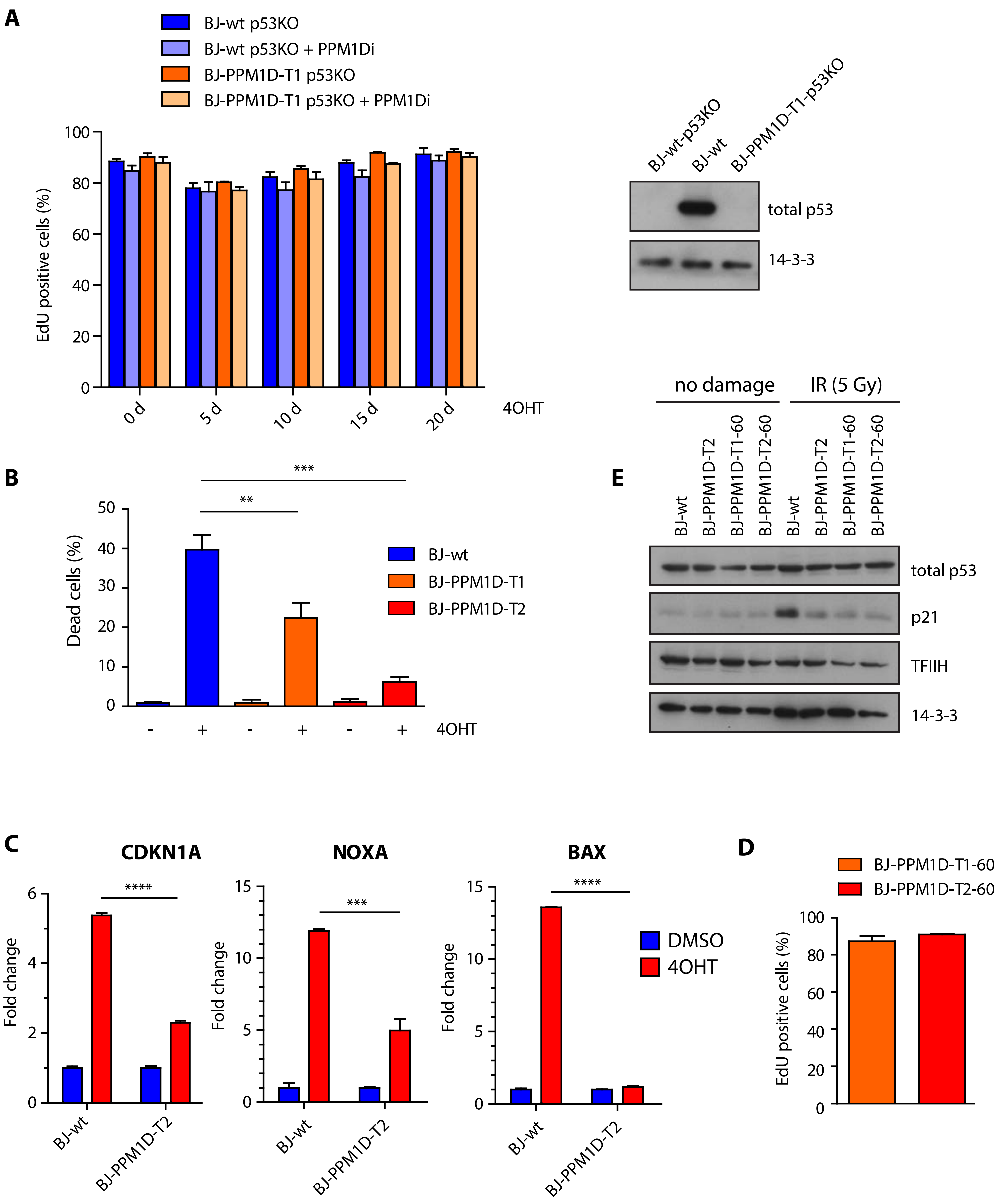

Suppl. Table 1. Expression profiling of the transformed RPE-PPM1D-T2-SA clones

RNA was isolated from nontransformed RPE-PPM1D-T2 cells and six transformed RPE-PPM1D-T2-SA clones. Sequencing libraries were prepared using RNA HyperPrep Kit and were sequenced on the NovaSeq 6000 system with mean coverage >120. All parts of RNAseq data analysis were conducted in R and RNAseq read counts were normalized using R package DESeq2. Fold change (FC) and log_2FC were calculated from normalized reads, nontransformed RPE-PPM1D-T2 cells were considered a reference. Significance of differential expression for each gene was evaluated by Fisher's t-test with simulated p-values and Holm's p-value correction for multiple comparisons.

Suppl. Table 2. Genotypes of the transformed RPE-PPM1D-T2-SA clones

Genomic DNA variants (rows) from whole exome sequencing detected in the parental RPE-PPM1D-T2 (clone 0) and transformed RPE-PPM1D-T2-SA clones 1-6. Description of columns: A. Chromosome localization. B-C. Coordinate start/end (HG19). C-D. Nucleotide reference (ref)/alternative (alt). F. Gene. G. Reference transcript (NCBI). H. Length of canonical protein (amino acids). I. Codon change. H. Affected exon (if exonic variant). L-S. Variant characteristics. T-AG. Variant found in parental (0) and subsequent (1-6) clones. First column indicates the zygosity (1=heterozygote; 2=homozygote; NA=variant is not present=wild-type), second column describe allelic fraction of an alternative variant (minor allele frequency). AH. Maximal alternative allele frequency (regardless of population-specific cohort in the gnomAD database). AI. Variant listed in OMIM database (0=no; 1=yes). A0 – characteristics of the variant in the ClinVar database (assessed in 2023-12-12).

Suppl. Table 3. GSEA analysis comparing individual RPE-PPM1D-T2-SA clones (#1-6) with RPE-PPM1D-T2 parental cells. The GSEA analysis included all datasets from the Human MSigDB Collections (https://www.gsea-msigdb.org/gsea/msigdb/human/collections.jsp) available in the org.Hs.eg.db R-

package (accessed 2024-06-24). The sheet “all” includes data from the analysis for all clones. The sheet “summary GSEA statistics” provides overview of analysed and significantly changed gene subsets.
